# A review of the use of propensity score diagnostics in papers published in high-ranking medical journals

**DOI:** 10.1186/s12874-020-00994-0

**Published:** 2020-05-27

**Authors:** Emily Granger, Tim Watkins, Jamie C. Sergeant, Mark Lunt

**Affiliations:** 1 Centre for Epidemiology Versus Arthritis, Centre for Musculoskeletal Research, Division of Musculoskeletal and Dermatological Sciences, School of Biological Sciences, Faculty of Biology, Medicine and Health, University of Manchester, Manchester, M13 9PT UK; 2 Department of Developmental Disability Neuropsychiatry, School of Psychiatry, University of New South Wales, Sydney, Australia; 3 Centre for Biostatistics, Division of Population Health, Health Services Research and Primary Care, School of Health Sciences, Faculty of Biology, Medicine and Health, University of Manchester, Manchester, M13 9PT UK

**Keywords:** Covariate balance, Confounding, Propensity scores, Diagnostics, Epidemiology

## Abstract

**Background:**

Propensity scores are widely used to deal with confounding bias in medical research. An incorrectly specified propensity score model may lead to residual confounding bias; therefore it is essential to use diagnostics to assess propensity scores in a propensity score analysis. The current use of propensity score diagnostics in the medical literature is unknown. The objectives of this study are to (1) assess the use of propensity score diagnostics in medical studies published in high-ranking journals, and (2) assess whether the use of propensity score diagnostics differs between studies (a) in different research areas and (b) using different propensity score methods.

**Methods:**

A PubMed search identified studies published in high-impact journals between Jan 1st 2014 and Dec 31st 2016 using propensity scores to answer an applied medical question. From each study we extracted information regarding how propensity scores were assessed and which propensity score method was used. Research area was defined using the journal categories from the Journal Citations Report.

**Results:**

A total of 894 papers were included in the review. Of these, 187 (20.9%) failed to report whether the propensity score had been assessed. Commonly reported diagnostics were *p*-values from hypothesis tests (36.6%) and the standardised mean difference (34.6%). Statistical tests provided marginally stronger evidence for a difference in diagnostic use between studies in different research areas (*p* = 0.033) than studies using different propensity score methods (*p* = 0.061).

**Conclusions:**

The use of diagnostics in the propensity score medical literature is far from optimal, with different diagnostics preferred in different areas of medicine. The propensity score literature may improve with focused efforts to change practice in areas where suboptimal practice is most common.

## Background

Whilst randomised controlled trials (RCTs) are the gold standard for evaluating treatment effects, they are often infeasible due to time, cost or ethical constraints. In such situations, observational data may provide valuable information. Unfortunately, observational data analyses are subject to confounding bias. This occurs when patient characteristics that influence the outcome have unbalanced distributions across treatment groups. Any differences observed in the outcome between treatment groups may be partly due to the differences in patient characteristics.

Traditionally, multivariable regression is used to account for the differences in patient characteristics between treatment groups. However, this approach is not always suitable. For example, when the study outcome is binary, a rule of thumb suggests that 10 events should be observed per covariate included in the regression model [1]. This could be infeasible if the outcome is rare and there are many covariates to adjust for. Propensity scores provide a potential solution to this problem. Rosenbaum and Rubin [2] first introduced the propensity score, defined as the probability of treatment assignment conditional on baseline characteristics. Additionally, they demonstrated that conditioning on the propensity score will balance the distribution of characteristics between treatment groups, reducing the chance of confounding bias. Propensity scores are useful for situations with rare binary outcomes because adjusting for the propensity score only is sufficient to improve balance on the measured covariates. They are also useful in situations where the relationship between covariates and treatment is better understood than the relationship between covariates and outcome, since treatment is modelled rather than outcome. Additionally, comparing propensity score distributions between treatment groups can help identify areas of non-overlap in covariate distributions, which are often overlooked when using traditional regression methods [3]. However, it is important to note that propensity scores cannot account for unmeasured confounding: balance will only be improved on covariates used to estimate the propensity score.

Most commonly, propensity scores are estimated using logistic regression. Treatment assignment is regressed on baseline characteristics and the predicted probabilities are the estimated propensity scores. Assuming no unmeasured confounding and no misspecification of the propensity score model, unbiased estimates of treatment effects can be obtained using one of four techniques: matching, stratification, weighting or covariate adjustment. We briefly describe these techniques here, but readers are referred elsewhere for more details [2, 4–9]. Matching involves forming matched sets of treated and control patients, on the basis of having similar propensity scores. Stratification involves dividing patients into equally sized strata based on their propensity score and weighting involves assigning propensity-based weights to each patient. Estimated treatment effects can then be obtained by comparing outcomes in the matched set, within strata (an overall estimate can be obtained by pooling the strata-specific estimates) or in the weighted sample. Finally, covariate adjustment is implemented by including the propensity score as a covariate when regressing outcome on treatment. Each of these techniques aim to balance patient characteristics between treatment groups, but misspecification of the propensity score model could prevent achieving adequate balance, thereby leading to residual confounding bias. Hence, an essential step of propensity score implementation is using appropriate diagnostics to assess the propensity score and ensure that it has adequately reduced confounding bias. Many authors [10–17] have made recommendations regarding appropriate use of diagnostics. More specifically, they recommended against the use of hypothesis tests comparing covariate means or proportions and advocated using standardised differences.

Despite their introduction in 1983, propensity scores were not commonly applied in the medical literature until around 20 years later. More recently, they have become increasingly popular [10]. In the last decade (2007–2017) the number of articles returned from searching ‘propensity scores’ in PubMed more than tripled over each 5 year period. Following the increase in use of propensity scores, a number of reviews [10, 11, 18–25] assessing their implementation were published. Regrettably, each review found that propensity score implementation was suboptimal, particularly regarding the use of diagnostics. Many authors were not reporting the use of any propensity score diagnostic, and those who did were often using hypothesis tests, which are widely discouraged. If appropriate diagnostics are not used to demonstrate the balance of potential confounders achieved by the propensity score, readers of the research have no basis for trusting the results. Of the existing reviews on the propensity score literature, only three [11, 19, 21] consider articles from all areas of medicine, and these collectively include articles published up to 2012. Since 2012, there has been numerous publications providing guidance on the use of propensity score diagnostics [10–12, 14–17], or proposing new propensity score diagnostics [26–29]. Considering these recent developments in methodology and guidance on practice, the use of propensity score diagnostics in recent medical studies may have improved. Therefore the aim of this review is to update the literature on diagnostic use, but with a focus on high-ranking journals. Such journals could be considered more influential as they are often looked towards as a beacon of best practice. Furthermore, it may beneficial to know which types of studies are more or less likely to report use of suboptimal diagnostics. This information could help us to identify pockets of good practice and areas where efforts to change practice should be focused. Bearing this in mind, the objectives of this review are to: (1) assess the use of propensity score diagnostics in medical studies published in high-ranking journals and (2) compare use of diagnostics between studies (a) in different research areas and (b) using different propensity score methods.

## Methods

### Search strategy

A PubMed search was conducted on 13th November 2017 to identify articles using propensity scores. We searched for articles with “propensity score” or “propensity matched” in the title, abstract or as a Medical Subject Heading (MeSH). The search was limited to publications between 2014 and 2016 and to journals which [1] were ranked in the top 10 by the 2013 Journal Citation Report (JCR) impact factor in any JCR medicine category and [2] had a JCR impact factor of at least 4 (the full text search string is given in Additional file 1).

### Study selection

Studies which used propensity scores to answer an applied medical question were included. This includes studies which aim to assess the effect of a health intervention (e.g. drugs or surgical intervention), or the effect of an exposure (e.g. alcohol), on a health related outcome. Studies were excluded if they were methodological, editorials, reviews or letters. Titles and abstracts were screened to assess eligibility.

### Data extraction

Eligible studies were checked manually by one author (EG) to extract information regarding which diagnostic was used to assess the propensity score and which method was used to condition on the propensity score. For eligible journals, the research area and impact factor was obtained from the JCR website (https://webofknowledge.com).

To assess the reliability of the manual search for data extraction, an automatic full-text search was conducted by a second author (TW) using FileLocator Pro to identify which of the included articles used the ‘standardised difference’, ‘c-statistic’ or the ‘Hosmer-Lemeshow test’ as a propensity score diagnostic. These diagnostics were selected because the variety of terms which could be used to refer to these diagnostics is limited compared to other diagnostics (e.g. a t-test may be referred to as hypothesis test, significance test, testing equality of means etc.). For any discrepancies between the two data extraction methods, the article in question was manually checked (EG). Most discrepancies (135/147; 92%) were due to the full-text search either incorrectly selecting articles which used the diagnostic for something other than propensity scores, or incorrectly omitting articles where the authors had referred to the diagnostic using different terminology.

### Data analysis

Multinomial logistic regression was used to investigate whether or not there were differences in diagnostic use between (a) studies in different research areas and (b) studies using different propensity score methods. In both models, the outcome was a categorical variable indicating which diagnostic was used: (1) hypothesis tests (2) standardised differences (3) c-statistic, (4) the Hosmer-Lemeshow test (5) eye-balling the data (i.e. informally assessing balance by scanning the values in a table or figure comparing covariate means between treatment groups), (6) other and (7) failed to report diagnostic use. The ‘other’ category comprised of diagnostics that were rarely observed (< 4% studies). The independent variables were indicators for either research area or propensity score method and the categories used are the same as those presented in Figs. 2, 3 and 4. Studies which used multiple diagnostics were included in the model once for each diagnostic used and this was accounted for by using a robust standard error estimator which accounts for non-independent observations [30].

The *p*-values associated with each model’s F-statistic were reported as an informal measure of the strength of evidence for differences in diagnostic use between research areas and studies using different propensity score methods. To further investigate possible differences in diagnostic use, we reported the proportion (and associated 95% confidence interval) of studies using each diagnostic by research area and propensity score method. The 95% confidence intervals for proportions were calculated using the following formula: $$ {f}^{-1}\left\{\mathit{\ln}\frac{\hat{p}}{1-\hat{p}}\pm {t}_{1-\raisebox{1ex}{$\alpha $}\!\left/ \!\raisebox{-1ex}{$2$}\right.,\vartheta}\frac{\hat{s}}{\hat{p}\left(1-\hat{p}\right)}\right\} $$,

where $$ {f}^{-1}(y)=\frac{e^y}{1+{e}^y} $$, $$ \hat{p} $$ and $$ \hat{s} $$ are estimates of the proportion and associated standard error respsecitvely, and $$ {t}_{1-\raisebox{1ex}{$\alpha $}\!\left/ \!\raisebox{-1ex}{$2$}\right.,\vartheta } $$ is the $$ {\left(1+\raisebox{1ex}{$\alpha $}\!\left/ \!\raisebox{-1ex}{$2$}\right.\right)}^{th} $$ quantile of Student’s *t* distribution with *ϑ* degrees of freedom. The logit transformation of the confidence interval was used to ensure that the limits lay between 0 and 1.

## Results

**Fig. 1:**
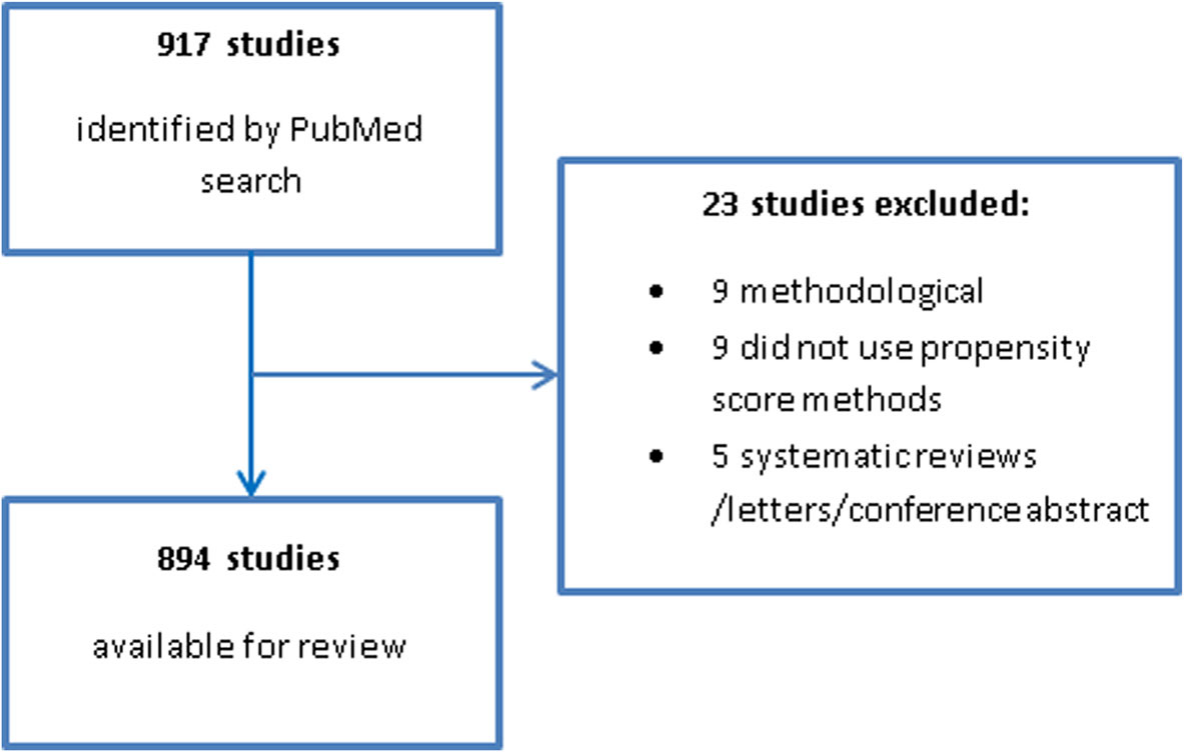
Flowchart of study selection

The PubMed search identified 917 studies, of which 23 did not meet the inclusion criteria (Fig. 1). The remaining 894 studies were included in the review (a list of the included studies is given in Additional file 2).

Of the 894 studies included in the review, 187 (20.9%) failed to report whether propensity score diagnostics were used. A further 21 (2.3%) reported that the propensity score had been assessed, but did not report how. Where diagnostics were reported, the most common were: *p*-values from hypothesis tests (36.6%), standardised difference (34.6%), c-statistic (15.4%) and the Hosmer-Lemeshow test (5.7%). Eye-balling the data after adjustment to assess balance was reported in (4.3%) studies. These percentages total to more than 100 since many authors used multiple diagnostics.

The average impact factor (median [interquartile range]) was highest among articles where balance was assessed by eye-balling the data (9.3 [6.0, 14.9]). Ordering the remaining diagnostic categories by median impact factor gives: propensity score assessed, but diagnostic not reported (7.2 [6.0, 12.5]), standardised difference (7.0 [5.6, 12.5]), c-statistic (7.0 [5.5, 11.9]), Hosmer-Lemeshow test (7.0 [5.4, 10.4]) *p*-values (7.0 [5.2, 9.8]), did not report diagnostic use (7.0 [5.3, 12.0]) and other (6.1 [5.3,10.7]). The similarity in these median indicates no discernible association between diagnostic and impact factor.

### Comparison of diagnostics between different research areas

**Fig. 2:**
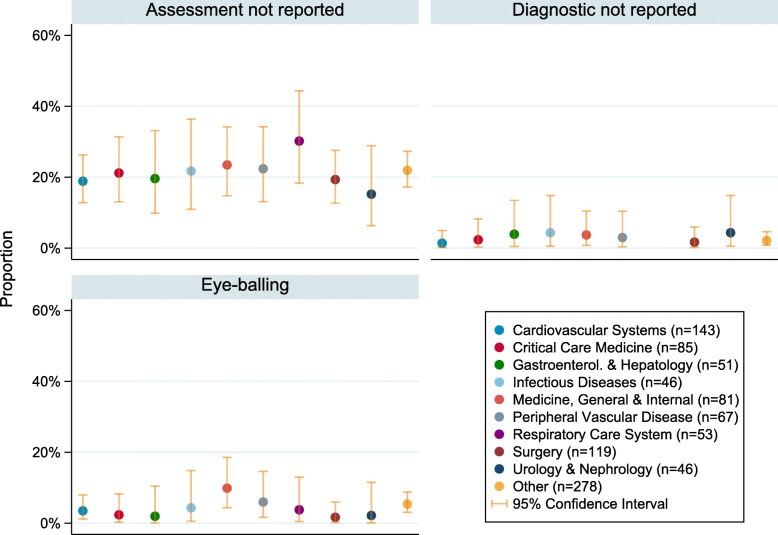
The proportion (95% Confidence Interval) of studies which did not report use of a propensity score diagnostic, by research area. ‘Assessment not reported’ refers to papers which did not specify whether propensity scores were assessed; ‘Diagnostic not reported’ refers to papers which reported that assessment took place, but not how

**Fig. 3:**
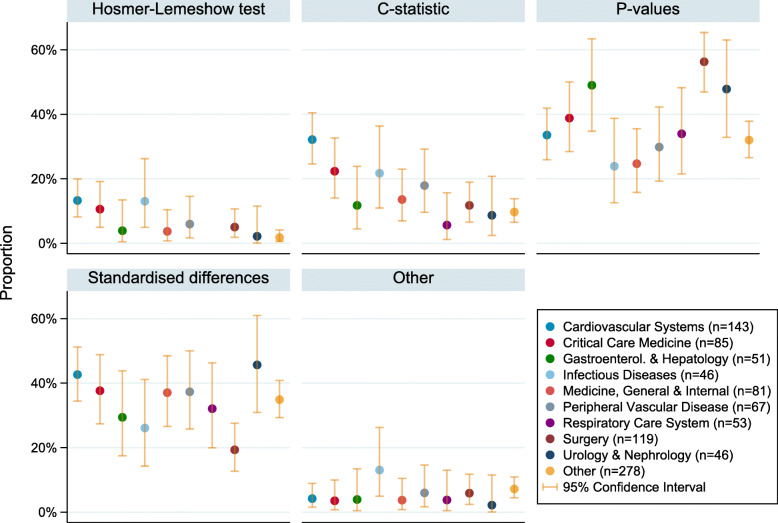
The proportion (95% Confidence Interval) of studies using each diagnostic, by research area. ‘Other’ includes: absolute differences, graphical approaches, post-matching c-statistic, regression, standardised bias, and variance ratios

The most common research areas (defined as areas containing 5% or more of the total number of studies) are listed in Figs. 2 and 3. There were 25 additional research areas; these have been grouped together into an ‘other’ category. The number of studies in each of the additional research areas was between 1 (0.11%) and 36 (4.03%). More details on the additional research areas and how many studies were in area is given in Additional file 3. For the most common research areas, the proportion of studies which either: (1) did not report whether balance assessment took place, (2) reported that balance assessment took place, but did not report which diagnostic was used or (3) relied on eyeballing the data for balance assessment, are given in Fig. 2, along with 95% confidence intervals. The equivalent proportions and 95% confidence intervals for studies using: the Hosmer-Lemeshow test, c-statistic, hypothesis tests, standardised differences or “other” are given in Fig. 3. The F-statistic associated with the multinomial regression model of diagnostic on research area had *p*-value = 0.033, suggesting a possible difference in diagnostic use between research areas.

Across all research areas, studies in respiratory care had the highest proportion of papers failing to report whether or not diagnostics were used (30% [95% CI, 18–44%]). In the remaining research areas, between 15 and 23% of studies failed to report this information.

There is little evidence for a difference in the use of eye-balling or Hosmer-Lemeshow test between research areas (as indicated by the overlapping confidence intervals), however there were some differences in the use of c-statistics, *p*-values (with hypothesis tests) and standardised differences.

Cardiovascular studies had the highest reported use of c-statistics (32% [95% CI, 25–40%]). For comparison, the research areas with the lowest reported use of c-statistics were urology and nephrology (9% [95% CI, 2–21%]) and respiratory care systems (6% [95% CI, 1–16%]).

Most research areas had approximately 25–50% of papers reporting the use of *p*-values. The area with the lowest proportion of reported use of p-values was infectious diseases (24% [95% CI, 13–39%]), followed by medicine general and internal (25% [95% CI, 16–36%]), cardiovascular systems (34% [95% CI, 26–42%]) and peripheral vascular disease (30% [95% CI, 13–39%]). Surgery studies had the highest proportion of reported use of p-values (56% [95% CI, 47–65%]).

On the other hand, surgery studies had the lowest proportion of papers reporting use of standardised differences (19% [95% CI, 13–28%]). The remaining research areas had at least 26% of papers reporting use of standardised differences and the highest proportion was in studies on urology and nephrology (46% [95% CI, 31–61%]).

### Comparison of diagnostics between studies using different propensity score methods

Of the 894 studies included, 693 (78%) used propensity score matching, 57 (6%) used stratification, 106 (12%) used covariate adjustment and 115 (13%) used weighted. These percentages total to more than 100 because some studies used multiple methods. Of those that reported use of weighting, 108 used inverse-probability-of-treatment weights, 3 used standardised-mortality-ratio weighting, 1 used overlap weights and 3 did not report which weights were used.

**Fig. 4:**
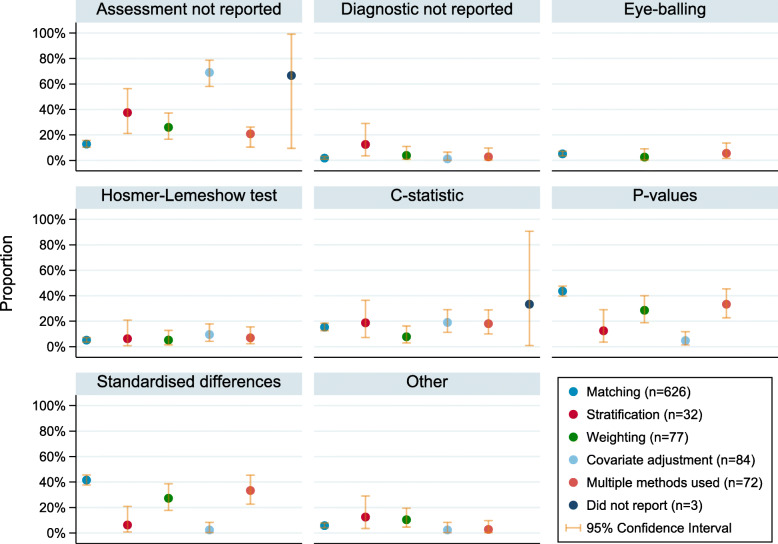
The proportion (95% Confidence Interval) of studies using each diagnostic, by propensity score method. Assessment not reported’ refers to papers which did not specify whether propensity scores were assessed; ‘Diagnostic not reported’ refers to papers which reported that assessment took place, but not how; ‘Other’ includes: absolute differences, graphical approaches, post-matching c-statistic, regression, standardised bias, and variance ratios

Figure 4 compares the proportions of studies using each diagnostic by propensity score method used. Studies using covariate adjustment for the propensity score were most likely to not report whether diagnostics were used. The proportion of covariate adjustment studies failing to report this information was 69% [95% CI, 58–79%], whereas for stratified, weighted and matched studies, the equivalent proportions were 38% [95% CI, 21–56%], 26% [95% CI, 17–37%] and 13% [95% CI, 10–16%] respectively.

While matched studies were the least likely to not report whether diagnostics were used, they were the most likely to report the use of *p*-values or standardised differences. Of the matched studies, 44% [95% CI, 40–48%] reported use of p-values, compared to 5% [95% CI, 1–12%] for covariate adjustment studies, and 42% [95% CI, 38–46%] of matched studies reported use of standardised differences, compared to 2% [95% CI, 0–8%] of covariate adjustment studies.

The proportion of reported use of eye-balling, the Hosmer-Lemeshow test or the c-statistic to assess propensity scores was very similar between studies using different methods. Although there were observed differences in the use of *p*-values, standardised difference and proportion of studies not reporting assessment between studies using different methods, the F-statistic associated with the multinomial regression model of diagnostic on propensity score method had p-value = 0.061, indicating weak evidence for differences overall.

## Discussion

A methodological review of 894 articles was conducted to assess the use of propensity score diagnostics in medical papers published in high-impact journals. Our results demonstrate that the use of propensity score diagnostics is suboptimal, even in high-impact papers, where best practice might be expected. Approximately one fifth of authors did not report the use of any diagnostic, and of those who did, hypothesis tests were the most common, despite the widespread discouragement of their use.

The problem with hypothesis tests is that they are dependent upon sample size and the hypotheses they test are not relevant. The former disadvantage is particularly problematic when matching; in the matched set an apparent improvement in balance may be due to the loss of statistical power after discarding observations. The hypotheses are irrelevant since they aim to test whether two population parameters are equal to each other, using sample data assumed to be drawn from those populations. However, when assessing propensity scores we are only interested in whether or not balance has been achieved in the sample used to estimate the treatment effects.

Additionally, our results indicated that the Hosmer-Lemeshow test and c-statistic were also among the most commonly used diagnostics for propensity scores. These diagnostics are traditionally used for logistic regression, where the former measures goodness-of-fit. Although a significant Hosmer-Lemeshow test can identify problems with the propensity score model, it lacks power and hence a non-significant result cannot be used as evidence that a propensity score model is correct [31]. The c-statistic measures model discrimination, which, in a propensity score context, is the ability to predict treatment assignment. However, propensity scores aim to balance confounding variables, and this may not improve with improved discrimination: including variables associated with the exposure but not the outcome will improve model discrimination, but may also increase bias and variance in estimated treatment effects [32, 33]. For these reasons, we recommend against using logistic regression diagnostics for propensity scores. An exception to this recommendation, is using the c-statistic as a post-matching diagnostic [34]. If the propensity score has adequately removed imbalances between treatment groups, patient characteristics should have no association with treatment assignment in the matched set. In this case, c-statistics close to 0.5 indicate successful balance.

Standardised differences were the most commonly used diagnostic in our results, after hypothesis testing. Standardised differences are preferred because they are independent of sample size and are a property of the sample [35]. It has been suggested that a standardised difference of less than 0.1 can be considered as adequate balance [36], however this is an arbitrary threshold. The levels of acceptable imbalance will likely depend on the strength of association between covariate and outcome: stronger predictors of outcome will contribute more towards confounding bias and balance should be prioritised on these variables [37]. Additionally, it is recommended to check standardised differences in second order moments and interaction terms, since failing to do so may prevent sufficient balance being achieved on non-linear or non-additive terms [13]. If sufficient balance is not reached, authors may consider adding higher order terms, three-way interactions, transforming variables or re-categorising variables [38].

Whereas previous reviews [10, 11, 18–25] did not limit their searches by journal impact factor, ours focused on high-impact journals and despite the differences in inclusion criteria there were still similarities in our results. Previous reviews [10, 11, 18–25] found that between 11 and 59% (20.9% in the current study) of authors did not report the use of any diagnostic; − unfortunately, there is no evidence to suggest that this percentage is decreasing over time. Furthermore, most previous reviews [10, 11, 18–20, 22–25] also found that hypothesis tests were the most common diagnostic (the only review [21] that did not report this finding did not go into detail about which diagnostics were used). We add to the current literature by demonstrating that even in high-impact journals, where we might expect best practice to be more common, many authors are still not reporting diagnostic use, or reporting use of suboptimal diagnostics. However, comparing our results to previous reviews does suggest an increase in the use of standardised differences over time. Including this one, three reviews reported the proportion of papers using standardised differences and considered papers across all areas of medicine. The proportions and publication years (breaking down our results by year) in increasing order were: 4% (1996–2003), 15% (2011–2012), 34% (2014), 35% (2015) and 42% (2016). The increase in use of standardised differences could be a result of the recent published recommendations for their use [10–17].

Our results indicated that there were some differences in diagnostic use between different research areas. It is likely that authors will read papers within their own area and follow the apparent norm when implementing these methods in their own work. Therefore, diagnostic use could improve if tutorial papers with best practice examples were published in the leading journals of those research areas where suboptimal practice is most common. When comparing diagnostic use between studies using different propensity score methods, we observed large differences in the reported use of diagnostics in matched studies compared to the other propensity score methods: matched studies were less likely to omit reporting diagnostic use and more likely to use standardised differences. By matching subjects’ results in distinct treatment groups, it is easy to compare means between groups, and so it is arguably easier to comprehend how to implement standardised differences when matching compared to the alternative methods. This could be the reason for the disparity in reported diagnostic use between matching and other methods. Fortunately there is existing guidance on how to use propensity score diagnostics when weighing [39] or using covariate adjustment [40], and well as when matching [13].

A limitation of the current study is that information regarding diagnostic use and propensity score method was only obtained manually by one author. However, we used a full-text search program to identify papers using three of the more commonly used diagnostics and thus could check for discrepancies. This revealed only a few errors; therefore, we assume that the manual search was similarly reliable for the other diagnostics. Secondly, the research areas are not necessarily categorised in the most informative way. Creating distinct and meaningful categories was challenging and there are a number of ways in which research areas could have been arbitrarily defined. We felt it was best to make use of a system already in place by using categories defined on the JCR website. Thirdly, our search may not have revealed all relevant literature. A recent study [41] found that many articles that use a particular methodology do not report its use in the title or abstract. Consequently, our review could have missed relevant articles which use propensity score methods. Moreover, by defining our search terms as “propensity score” or “propensity matched”, it is possible that studies using inverse probability of treatment weighting could be underrepresented. However, the proportion of studies in our results that used this weighting method (12%) is similar to that observed in other reviews [11, 22–25] on the use of propensity scores (0–14%).

Finally, regarding diagnostic use, we only collected information on which diagnostics were used and not on how they were implemented. For example, when standardised differences are used, they may be used to check balance in different samples (e.g. a matched set or a weighted sample), different thresholds for acceptable balance may be used and residual imbalance s may be handled in a variety of ways. Future research could investigate how propensity score diagnostics are implemented in the medical literature: this could help inform best practice.

## Conclusions

In conclusion, the use of diagnostics in the propensity score literature remains suboptimal. Many authors are still failing to report whether diagnostics were used to assess the propensity score. However, over time we have seen some improvement. Standardised differences are currently the most widely recommended diagnostic and we have seen an increase in their use compared to previous reviews. Additionally, we identified the research areas in which suboptimal practice was most common. The propensity score literature may benefit from focused efforts to improve practice in these areas. .

## Supplementary information

**Additional file 1.** Full text search string used to identify articles

**Additional file 2.** List of included studies

**Additional file 3.** Further details on Research Areas

## Data Availability

The dataset generated and analysed during the current study is available in the PropensityScoreReview repository: https://github.com/EmilyG602/PropensityScoreReview

## Abbreviations

JCR: Journal Citation Report
CI: Confidence Interval
