# Supplementary material for: A review of the use of propensity score diagnostics in papers published in high-ranking medical journals
Source: BMC Med Res Methodol. 2020 May 27;20:132. doi: 10.1186/s12874-020-00994-0 (PMC7251670; doi:10.1186/s12874-020-00994-0)
Supplement: Supplementary file 1 — Additional file 1. Full text search string used to identify articles [file 12874_2020_994_MOESM1_ESM.docx]

Additional file 1: Full text search string used to identify articles

2014:2016[dp] AND humans[mh] AND (propensity score[tiab] OR propensity matched[tiab] OR propensity score[mh])

AND

("Acta Derm Venereol"[ta] OR "Acta Neuropathol"[ta] OR "Addict Biol"[ta] OR "Addiction"[ta] OR "Adv Drug Deliv Rev"[ta] OR "Adv Nutr"[ta] OR "Ageing Res Rev"[ta] OR "Aging Cell"[ta] OR "AIDS"[ta] OR "Aliment Pharmacol Ther"[ta] OR "Allergy"[ta] OR "Alzheimers Dement"[ta] OR "Am J Clin Nutr"[ta] OR "Am J Gastroenterol"[ta] OR "Am J Kidney Dis"[ta] OR "Am J Ophthalmol"[ta] OR "Am J Pathol"[ta] OR "Am J Physiol Lung Cell Mol Physiol"[ta] OR "Am J Psychiatry"[ta] OR "Am J Respir Cell Mol Biol"[ta] OR "Am J Respir Crit Care Med"[ta] OR "Am J Respir Crit Care Med"[ta] OR "Am J Sports Med"[ta] OR "Am J Surg Pathol"[ta] OR "Am J Transplant"[ta] OR "Anesthesiology"[ta] OR "Angiogenesis"[ta] OR "Ann Emerg Med"[ta] OR "Ann Fam Med"[ta] OR "Ann Intern Med"[ta] OR "Ann Neurol"[ta] OR "Ann Rheum Dis"[ta] OR "Ann Surg"[ta] OR "Annu Rev Immunol"[ta] OR "Annu Rev Med"[ta] OR "Annu Rev Nutr"[ta] OR "Annu Rev Pathol"[ta] OR "Annu Rev Pharmacol"[ta] OR OR "Annu Rev Public Health"[ta] OR "Antioxid Redox Signal"[ta] OR "Arch Neurol"[ta] OR "Arch Ophthalmol"[ta] OR "Arch Pediatr Adolesc Med"[ta] OR "Arch Toxicol"[ta] OR "Arterioscler Thromb Vasc Biol"[ta] OR "Arthritis Care Res (Hoboken)"[ta] OR "Arthritis Res Ther"[ta] OR "Arthritis Rheumatol"[ta] OR "Atheroscler Suppl"[ta] OR "Biol Psychiatry"[ta] OR "Blood Rev"[ta] OR "Blood"[ta] OR "BMC Med"[ta] OR "BMJ"[ta] OR "Br J Anaesth"[ta] OR "Br J Dermatol"[ta] OR "Br J Psychiatry"[ta] OR "Br J Sports Med"[ta] OR "Br J Surg"[ta] OR "Brain"[ta] OR "Bull World Health Organ"[ta] OR "CA Cancer J Clin"[ta] OR "Cancer Cell"[ta] OR "Cancer Discov"[ta] OR "Cancer Res"[ta] OR "Cell Metab"[ta] OR "Chest"[ta] OR "Circ Cardiovasc Imaging"[ta] OR "Circ Cardiovasc Interv"[ta] OR "Circ Res"[ta] OR "Circulation"[ta] OR "Clin Exp Allergy"[ta] OR "Clin Gastroenterol Hepatol"[ta] OR "Clin Infect Dis"[ta] OR "Clin J Am Soc Nephrol"[ta] OR "Clin Microbiol Infect"[ta] OR "Clin Pharmacol Ther"[ta] OR "Clin Rev Allergy Immunol"[ta] OR "Cochrane Database Syst Rev"[ta] OR "Cold Spring Harb Perspect Med"[ta] OR "Crit Care Med"[ta] OR "Crit Care"[ta] OR "Crit Rev Food Sci Nutr"[ta] OR "Crit Rev Toxicol"[ta] OR "Curr Opin Infect Dis"[ta] OR "Curr Opin Lipidol"[ta] OR "Curr Opin Nephrol Hypertens"[ta] OR "Curr Opin Rheumatol"[ta] OR "Dent Mater"[ta] OR "Diabetes Care"[ta] OR "Diabetes"[ta] OR "Dis Model Mech"[ta] OR "Drug Resist Updat"[ta] OR "EMBO Mol Med"[ta] OR "Emerg Infect Dis"[ta] OR "Endocr Rev"[ta] OR "Endoscopy"[ta] OR "Environ Health Perspect"[ta] OR "Epidemiol Rev"[ta] OR "Epidemiology"[ta] OR "Eur Heart J"[ta] OR "Eur J Epidemiol"[ta] OR "Eur J Heart Fail"[ta] OR "Eur J Nucl Med Mol Imaging"[ta] OR "Eur Respir J"[ta] OR "Eur Urol"[ta] OR "Euro Surveill"[ta] OR "Exerc Immunol Rev"[ta] OR "Exerc Sport Sci Rev"[ta] OR "Exp Dermatol"[ta] OR "Fertil Steril"[ta] OR "Forensic Toxicol"[ta] OR "Front Neuroendocrinol"[ta] OR "Gastroenterology"[ta] OR "Gut"[ta] OR "Haematologica"[ta] OR "Health Aff (Millwood)"[ta] OR "Health Technol Assess"[ta] OR "Hepatology"[ta] OR "Hum Brain Mapp"[ta] OR "Hum Reprod Update"[ta] OR "Hum Reprod"[ta] OR "Hypertension"[ta] OR "Immunity"[ta] OR "Immunol Rev"[ta] OR "Inflamm Bowel Dis"[ta] OR "Int J Epidemiol"[ta] OR "Int J Obes (Lond)"[ta] OR "Intensive Care Med"[ta] OR "J Acquir Immune Defic Syndr"[ta] OR "J Allergy Clin Immunol"[ta] OR "J Am Acad Child Psychiatry"[ta] OR "J Am Acad Dermatol"[ta] OR "J Am Coll Cardiol"[ta] OR "J Am Coll Surg"[ta] OR "J Am Geriatr Soc"[ta] OR "J Am Med Assoc"[ta] OR "J Am Med Dir Assoc"[ta] OR "J Am Soc Nephrol"[ta] OR "J Antimicrob Chemother"[ta] OR "J Bone Joint Surg Am"[ta] OR "J Bone Joint Surg Am"[ta] OR "J Cachexia Sarcopenia Muscle"[ta] OR "J Cardiovasc Magn Reson"[ta] OR "J Cereb Blood Flow Metab"[ta] OR "J Clin Epidemiol"[ta] OR "J Clin Invest"[ta] OR "J Clin Oncol"[ta] OR "J Dent Res"[ta] OR "J Exp Med"[ta] OR "J Gerontol A Biol Sci Med Sci"[ta] OR "J Heart Lung Transplant"[ta] OR "J Heart Lung Transplant"[ta] OR "J Hepatol"[ta] OR "J Infect Dis"[ta] OR "J Invest Dermatol"[ta] OR "J Med Internet Res"[ta] OR "J Med Internet Res"[ta] OR "J Natl Cancer Inst"[ta] OR "J Neurol Neurosurg Psychiatry"[ta] OR "J Neuropathol Exp Neurol"[ta] OR "J Nucl Med"[ta] OR "J Nutr Biochem"[ta] OR "J Pathol"[ta] OR "J Pineal Res"[ta] OR "J Psychiatry Neurosci"[ta] OR "J Thorac Oncol"[ta] OR "J Thromb Haemost"[ta] OR "J Toxicol Environ Health B Crit Rev"[ta] OR "JACC Cardiovasc Imaging"[ta] OR "JACC Cardiovasc Interv"[ta] OR "JAMA Dermatol"[ta] OR "JAMA Intern Med"[ta] OR "JAMA Psychiatry"[ta] OR "Kidney Int"[ta] OR "Lancet Infect Dis"[ta] OR "Lancet Neurol"[ta] OR "Lancet Oncol"[ta] OR "Lancet"[ta] OR "Leukemia"[ta] OR "Med Res Rev"[ta] OR "Med Sci Sports Exerc"[ta] OR "Milbank Q"[ta] OR "Mod Pathol"[ta] OR "Mol Aspects Med"[ta] OR "Mol Psychiatry"[ta] OR "Mutat Res Rev Mutat Res"[ta] OR "N Engl J Med"[ta] OR "Nanotoxicology"[ta] OR "Nat Immunol"[ta] OR "Nat Med"[ta] OR "Nat Rev Cancer"[ta] OR "Nat Rev Cardiol"[ta] OR "Nat Rev Clin Oncol"[ta] OR "Nat Rev Drug Discov"[ta] OR "Nat Rev Endocrinol"[ta] OR "Nat Rev Gastroenterol Hepatol"[ta] OR "Nat Rev Immunol"[ta] OR "Nat Rev Nephrol"[ta] OR "Nat Rev Neurol"[ta] OR "Nat Rev Rheumatol"[ta] OR "Nat Rev Urol"[ta] OR "Neurobiol Aging"[ta] OR "Neuroimage"[ta] OR "Neurology"[ta] OR "Neuropathol Appl Neurobiol"[ta] OR "Neuropsychopharmacology"[ta] OR "Neurorehabil Neural Repair"[ta] OR "Neuroscientist"[ta] OR "Nutr Rev"[ta] OR "Obes Rev"[ta] OR "Obesity (Silver Spring)"[ta] OR "Obstet Gynecol"[ta] OR "Ocul Surf"[ta] OR "Ophthalmology"[ta] OR "Osteoarthritis Cartilage"[ta] OR "Pain"[ta] OR "Part Fibre Toxicol"[ta] OR "Pediatrics"[ta] OR "Pharmacol Rev"[ta] OR "Pharmacol Ther"[ta] OR "Pigment Cell Melanoma Res"[ta] OR "PLoS Med"[ta] OR "PLoS Negl Trop Dis"[ta] OR "Proc Nutr Soc"[ta] OR "Prog Lipid Res"[ta] OR "Prog Retin Eye Res"[ta] OR "Psychother Psychosom"[ta] OR "Radiology"[ta] OR "Radiother Oncol"[ta] OR "Rheumatology"[ta] OR "Schizophr Bull"[ta] OR "Sci Transl Med"[ta] OR "Semin Immunopathol"[ta] OR "Sleep Med Rev"[ta] OR "Sports Med"[ta] OR "Stem Cells Dev"[ta] OR "Stem Cells"[ta] OR "Stroke"[ta] OR "Surg Obes Relat Dis"[ta] OR "Theranostics"[ta] OR "Thorax"[ta] OR "Thromb Haemost"[ta] OR "Tob Control"[ta] OR "Toxicol Sci"[ta] OR "Trends Endocrinol Metab"[ta] OR "Trends Immunol"[ta] OR "Trends Mol Med"[ta] OR "Trends Pharmacol Sci"[ta] OR "Ultraschall Med"[ta] OR "World Psychiatry"[ta])

NOT

(2013[ppdat] OR 2017[ppdat] OR review[pt] OR systematic[sb] OR simulation[tiab] OR comment[pt] OR editorial[pt])
