# Supplementary material for: A review of the use of propensity score diagnostics in papers published in high-ranking medical journals
Source: BMC Med Res Methodol. 2020 May 27;20:132. doi: 10.1186/s12874-020-00994-0 (PMC7251670; doi:10.1186/s12874-020-00994-0)
Supplement: Supplementary file 2 — Additional file 2. List of included studies [file 12874_2020_994_MOESM2_ESM.docx]

Additional file 2: List of included studies

1. Markar, S.R., et al. Significance of Microscopically Incomplete Resection Margin After Esophagectomy for Esophageal. *Cancer Ann Surg*. 2016
2. Abdel-Wahab, M., et al. Aortic regurgitation after transcatheter aortic valve implantation with balloon- and self-expandable prostheses: a pooled analysis from a 2-center experience. *JACC Cardiovasc Interv*. 2014
3. Abe, T., et al. Association between helicopter with physician versus ground emergency medical services and survival of adults with major trauma in Japan. *Crit Care*. 2014
4. Ache, K., et al. Are advance directives associated with better hospice care? *J Am Geriatr Soc* 2014
5. Aggarwal, V., et al. Safety and effectiveness of drug-eluting versus bare-metal stents in saphenous vein bypass graft percutaneous coronary interventions: insights from the Veterans Affairs CART program*. J Am Coll Cardiol*. 2014
6. Alexopoulos, D., et al. Ticagrelor vs prasugrel one-month maintenance therapy: impact on platelet reactivity and bleeding events. *Thromb Haemost*. 2014
7. Alonso, A., et al. Intracranial hemorrhage mortality in atrial fibrillation patients treated with dabigatran or warfarin. *Stroke*. 2014
8. Alves, M.D., et al. Effect of cefepime dose on mortality of patients with Gram-negative bacterial bloodstream infections: a prospective cohort study. *J Antimicrob Chemother.* 2014
9. Andersson, C., et al. Association of beta-blocker therapy with risks of adverse cardiovascular events and deaths in patients with ischemic heart disease undergoing noncardiac surgery: a Danish nationwide cohort study. *JAMA Intern Med.* 2014
10. Antoniou, T., et al. Statins and the risk of herpes zoster: a population-based cohort study. *Clin Infect Dis.* 2014
11. Apostolakis, S., et al. Hormone replacement therapy and adverse outcomes in women with atrial fibrillation: an analysis from the atrial fibrillation follow-up investigation of rhythm management trial. *Stroke*. 2014
12. Baddley, J.W., et al. Non-viral opportunistic infections in new users of tumour necrosis factor inhibitor therapy: results of the SAfety Assessment of Biologic ThERapy (SABER) study. *Ann Rheum Dis*. 2014
13. Bangalore, S., et al. 2014 Eighth Joint National Committee panel recommendation for blood pressure targets revisited: results from the INVEST study. *J Am Coll Cardiol*. 2014
14. Bangalore, S., et al. Heparin monotherapy or bivalirudin during percutaneous coronary intervention in patients with non-ST-segment-elevation acute coronary syndromes or stable ischemic heart disease: results from the Evaluation of Drug-Eluting Stents and Ischemic Events registry. *Circ Cardiovasc Interv*. 2014
15. Bashir, R., et al. Comparative outcomes of catheter-directed thrombolysis plus anticoagulation vs anticoagulation alone to treat lower-extremity proximal deep vein thrombosis. *JAMA Intern Med*. 2014
16. Beattie, C.J., et al. Allopurinol initiation and change in blood pressure in older adults with hypertension. *Hypertension*. 2014
17. Berkowitz, S.A., et al. Initial choice of oral glucose-lowering medication for diabetes mellitus: a patient-centered comparative effectiveness study. *JAMA Intern Med*. 2014
18. Blot, S., et al. Does contemporary vancomycin dosing achieve therapeutic targets in a heterogeneous clinical cohort of critically ill patients? Data from the multinational DALI study. *Crit Care*. 2014
19. Byrne, D.D., et al. Risk of hip fracture associated with untreated and treated chronic hepatitis B virus infection. *J Hepatol*. 2014
20. Cavazzuti, I., et al. Early therapy with IgM-enriched polyclonal immunoglobulin in patients with septic shock. *Intensive Care Med*. 2014
21. Chancharoenthana, W., et al. The outcomes of kidney transplantation in hepatitis B surface antigen (HBsAg)-negative recipients receiving graft from HBsAg-positive donors: a retrospective, propensity score-matched study. *Am J Transplant*. 2014
22. Chang, J.S., et al. Preoperative chemoradiotherapy effects on anastomotic leakage after rectal cancer resection: a propensity score matching analysis. *Ann Surg*. 2014
23. Chao, P.W., et al. Association of postdischarge rehabilitation with mortality in intensive care unit survivors of sepsis. *Am J Respir Crit Care Med*. 2014
24. Chatu, S., et al. The impact of timing and duration of thiopurine treatment on first intestinal resection in Crohn's disease: national UK population-based study 1989-2010. *Am J Gastroenterol*. 2014
25. Chen, A.B., et al. Comparative effectiveness of intensity-modulated versus 3D conformal radiation therapy among medicare patients with stage III lung cancer. *J Thorac Oncol.* 2014
26. Chen, Y.Y., et al. Two distinct Do-Not-Resuscitate protocols leaving less to the imagination: an observational study using propensity score matching. *BMC Med*. 2014
27. Chien, A.T., et al. Two-year impact of the alternative quality contract on pediatric health care quality and spending. *Pediatrics.* 2014
28. Chung, S.W., et al. Prognostic effect of erroneous surgical procedures in patients with osteosarcoma: evaluation using propensity score matching. *J Bone Joint Surg Am*. 2014
29. Chung, T.K., et al. Examining national outcomes after thyroidectomy with nerve monitoring. *J Am Coll Surg*. 2014
30. Cole, T., et al. Usage of recombinant human bone morphogenetic protein in cervical spine procedures: analysis of the MarketScan longitudinal database. *J Bone Joint Surg Am*. 2014
31. Collaborative, S. Impact of postoperative non-steroidal anti-inflammatory drugs on adverse events after gastrointestinal surgery. *Br J Surg*. 2014
32. Contreras, G., et al. Comparison of mortality of ESRD patients with lupus by initial dialysis modality. *Clin J Am Soc Nephrol*. 2014
33. Corsonello, A., et al. Proton pump inhibitors and functional decline in older adults discharged from acute care hospitals*. J Am Geriatr Soc*. 2014
34. Cremer, P.C., et al. Myocardial perfusion imaging in emergency department patients with negative cardiac biomarkers: yield for detecting ischemia, short-term events, and impact of downstream revascularization on mortality. *Circ Cardiovasc Imaging*. 2014
35. Crews, D.C., et al. Predialysis health, dialysis timing, and outcomes among older United States adults. *J Am Soc Nephrol*. 2014
36. Danchin, N., et al. Five-year survival in patients with ST-segment-elevation myocardial infarction according to modalities of reperfusion therapy: the French Registry on Acute ST-Elevation and Non-ST-Elevation Myocardial Infarction (FAST-MI) 2005. *Cohort Circulation*. 2014
37. de la Torre Hernandez, J.M., et al. Clinical impact of intravascular ultrasound guidance in drug-eluting stent implantation for unprotected left main coronary disease: pooled analysis at the patient-level of 4 registries. *JACC Cardiovasc Interv*. 2014
38. de Mestral, C., et al. Comparative operative outcomes of early and delayed cholecystectomy for acute cholecystitis: a population-based propensity score analysis. *Ann Surg*. 2014
39. Dejam, A., et al. The effect of age and clinical circumstances on the outcome of red blood cell transfusion in critically ill patients. *Crit Care*. 2014
40. Doran, B., et al. Prognostic value of fasting versus nonfasting low-density lipoprotein cholesterol levels on long-term mortality: insight from the National Health and Nutrition Examination Survey III (NHANES-III). *Circulation*. 2014
41. Duchman, K.R., et al. Differences in short-term complications between unicompartmental and total knee arthroplasty: a propensity score matched analysis. *J Bone Joint Surg Am.* 2014
42. Ducloux, D., et al. Polyclonal antithymocyte globulin and cardiovascular disease in kidney transplant recipients. *J Am Soc Nephrol*. 2014
43. Dusetzina, S.B., et al. Cost sharing and adherence to tyrosine kinase inhibitors for patients with chronic myeloid leukemia. *J Clin Oncol.* 2014
44. Edelstein, A.I., et al. Impact of Resident Involvement on Orthopaedic Surgery Outcomes: An Analysis of 30,628 Patients from the American College of Surgeons National Surgical Quality Improvement Program Database. *J Bone Joint Surg Am.* 2014
45. El Malki, H.O., et al. Radical versus conservative surgical treatment of liver hydatid cysts. *Br J Surg*. 2014
46. Ensor, T., et al. Mobilizing communities to improve maternal health: results of an intervention in rural Zambia Bull World Health. *Organ*. 2014
47. Erdman, M.J., et al. A comparison of severe hemodynamic disturbances between dexmedetomidine and propofol for sedation in neurocritical care patients. *Crit Care Med.* 2014
48. Ezer, N., et al. Cisplatin vs. carboplatin-based chemoradiotherapy in patients >65 years of age with stage III non-small cell lung cancer. *Radiother Oncol.* 2014
49. Faillie, J.L., et al. Incretin based drugs and risk of acute pancreatitis in patients with type 2 diabetes: cohort study. *Bmj*. 2014
50. Fernandez-Ruiz, M., et al. Initial use of echinocandins does not negatively influence outcome in Candida parapsilosis bloodstream infection: a propensity score analysis. *Clin Infect Dis.* 2014
51. Fischer, J.P., et al. Propensity-matched, longitudinal outcomes analysis of complications and cost: comparing abdominal free flaps and implant-based breast reconstruction. *J Am Coll Surg*. 2014
52. Fisher, B.T., et al. Antifungal prophylaxis associated with decreased induction mortality rates and resources utilized in children with new-onset acute myeloid leukemia. *Clin Infect Dis*. 2014
53. Flaker, G., et al. Amiodarone, anticoagulation, and clinical events in patients with atrial fibrillation: insights from the ARISTOTLE trial. *J Am Coll Cardiol*. 2014
54. Frenette, A.J., et al. Albumin administration is associated with acute kidney injury in cardiac surgery: a propensity score analysis. *Crit Care.* 2014
55. Friberg, L. Safety of dronedarone in routine clinical care. *J Am Coll Cardiol*. 2014
56. Frohlich, G.M., et al. Long-term survival in patients undergoing percutaneous interventions with or without intracoronary pressure wire guidance or intracoronary ultrasonographic imaging: a large cohort study. *JAMA Intern Med*. 2014
57. Fukuda, H., et al. Hepatocellular carcinoma: concomitant sorafenib promotes necrosis after radiofrequency ablation--propensity score matching analysis. *Radiology*. 2014
58. Funk, D., et al. Low-dose corticosteroid treatment in septic shock: a propensity-matching study. *Crit Care Med*. 2014
59. Gagne, J.J., et al. Comparative effectiveness of generic and brand-name statins on patient outcomes: a cohort study. *Ann Intern Med*. 2014
60. Gandaglia, G., et al. The effect of neoadjuvant chemotherapy on perioperative outcomes in patients who have bladder cancer treated with radical cystectomy: a population-based study. *Eur Urol*. 2014
61. Gandaglia, G., et al. Gonadotropin-releasing hormone agonists and acute kidney injury in patients with prostate cancer. *Eur Urol.* 2014
62. Garnacho-Montero, J., et al. De-escalation of empirical therapy is associated with lower mortality in patients with severe sepsis and septic shock Intensive. *Care Med*. 2014
63. Gelbard, R., et al. Effect of delaying same-admission cholecystectomy on outcomes in patients with diabetes. *Br J Surg.* 2014
64. Gerhard, T., et al. Comparative mortality risks of antipsychotic medications in community-dwelling older adults. *Br J Psychiatry*. 2014
65. Gershengorn, H.B., et al. Association between arterial catheter use and hospital mortality in intensive care units. *JAMA Intern Med*. 2014
66. Goel, S.S., et al. Renin-angiotensin system blockade therapy after surgical aortic valve replacement for severe aortic stenosis: a cohort study. *Ann Intern Med.* 2014
67. Gordon, S.C., et al. Antiviral therapy for chronic hepatitis B virus infection and development of hepatocellular carcinoma in a US population. *Clin Gastroenterol Hepatol.* 2014
68. Gosmanova, E.O., et al. Association of medical treatment nonadherence with all-cause mortality in newly treated hypertensive US veterans. *Hypertension*. 2014
69. Goudie, A., et al. Attributable cost and length of stay for central line-associated bloodstream infections. *Pediatrics*. 2014
70. Grindem, H., et al. Nonsurgical or Surgical Treatment of ACL Injuries: Knee Function, Sports Participation, and Knee Reinjury: The Delaware-Oslo ACL Cohort Study. *J Bone Joint Surg Am*. 2014
71. Gronnier, C., et al. Impact of neoadjuvant chemoradiotherapy on postoperative outcomes after esophageal cancer resection: results of a European multicenter study. *Ann Surg*. 2014
72. Hamady, Z.Z., et al. One-millimeter cancer-free margin is curative for colorectal liver metastases: a propensity score case-match approach. *Ann Surg.* 2014
73. Hamandi, B., et al. Impact of infectious disease consultation on the clinical and economic outcomes of solid organ transplant recipients admitted for infectious complications. *Clin Infect Dis*. 2014
74. Hanna, D.B., et al. Increase in single-tablet regimen use and associated improvements in adherence-related outcomes in HIV-infected women*. J Acquir Immune Defic Syndr*. 2014
75. Hannan, E.L., et al. Coronary artery bypass graft surgery versus drug-eluting stents for patients with isolated proximal left anterior descending disease. *J Am Coll Cardiol.* 2014
76. Hayes, D., Jr., et al. Pulmonary hypertension in cystic fibrosis with advanced lung disease. *Am J Respir Crit Care Med*. 2014
77. Hermans, G., et al. Acute outcomes and 1-year mortality of intensive care unit-acquired weakness. A cohort study and propensity-matched analysis. *Am J Respir Crit Care Med*. 2014
78. Hesler, B.D., et al. Association between fibromyalgia and adverse perioperative outcomes. *Br J Anaesth*. 2014
79. Heyworth, L., et al. Influence of shared medical appointments on patient satisfaction: a retrospective 3-year study. *Ann Fam Med*. 2014
80. Hlatky, M.A., et al. Adoption and effectiveness of internal mammary artery grafting in coronary artery bypass surgery among Medicare beneficiaries. *J Am Coll Cardiol.* 2014
81. Hlatky, M.A., et al. Economic outcomes in the Study of Myocardial Perfusion and Coronary Anatomy Imaging Roles in Coronary Artery Disease registry: the SPARC Study. *J Am Coll Cardiol*. 2014
82. Hollm-Delgado, M.G., et al. Acute lower respiratory infection among Bacille Calmette-Guerin (BCG)-vaccinated children. *Pediatrics.* 2014
83. Hsiao, F.Y., et al. Dose-responsive effect of psychotropic drug use and subsequent dementia: a nationwide propensity score matched case-control study in Taiwan. *J Am Med Dir Assoc.* 2014
84. Huybrechts, K.F., et al. Antidepressant use in pregnancy and the risk of cardiac defects. *N Engl J Med.* 2014
85. Hynes, B.G., et al. Carotid artery stenting for recurrent carotid artery restenosis after previous ipsilateral carotid artery endarterectomy or stenting: a report from the National Cardiovascular Data Registry*. JACC Cardiovasc Interv.* 2014
86. Ibrahim, G.M., et al. Impaired development of intrinsic connectivity networks in children with medically intractable localization-related epilepsy. *Hum Brain Mapp.* 2014
87. Iqbal, M.B., et al. Radial versus femoral access is associated with reduced complications and mortality in patients with non-ST-segment-elevation myocardial infarction: an observational cohort study of 10,095 patients.  *Circ Cardiovasc Interv.* 2014
88. Iwagami, M., et al. Postoperative polymyxin B hemoperfusion and mortality in patients with abdominal septic shock: a propensity-matched analysis. *Crit Care Med.* 2014
89. Jackson, E.A., et al. Impact of sex on morbidity and mortality rates after lower extremity interventions for peripheral arterial disease: observations from the Blue Cross Blue Shield of Michigan Cardiovascular Consortium. *J Am Coll Cardiol.* 2014
90. Jenkinson, R.J., et al. Delayed wound closure increases deep-infection rate associated with lower-grade open fractures: a propensity-matched cohort study. *J Bone Joint Surg Am*. 2014
91. Jeschke, M.G., et al. Hypoglycemia is associated with increased postburn morbidity and mortality in pediatric patients. *Crit Care Med*. 2014
92. Johnsen, S.P., et al. Preadmission oral anticoagulant treatment and clinical outcome among patients hospitalized with acute stroke and atrial fibrillation: a nationwide study. *Stroke.* 2014
93. Johri, M., et al. Estimation of maternal and child mortality one year after user-fee elimination: an impact evaluation and modelling study in Burkina Faso. *Bull World Health* *Organ.* 2014
94. Jones, D.A., et al. Mortality in South Asians and Caucasians after percutaneous coronary intervention in the United Kingdom: an observational cohort study of 279,256 patients from the BCIS (British Cardiovascular Intervention Society) National Database.  *JACC Cardiovasc Interv*. 2014
95. Joo, H., et al. Cost of informal caregiving associated with stroke among the elderly in the United States. *Neurology*. 2014
96. Kadakia, M.B., et al. Factors associated with vascular complications in patients undergoing balloon-expandable transfemoral transcatheter aortic valve replacement via open versus percutaneous approaches. *Circ Cardiovasc Interv*. 2014
97. Kainuma, S., et al. Restrictive mitral annuloplasty with or without surgical ventricular reconstruction in ischaemic cardiomyopathy: impacts on neurohormonal activation, reverse left ventricular remodelling and survival. *Eur J Heart Fail.* 2014
98. Kamezaki, F., et al. Plasma levels of nitric oxide metabolites are markedly reduced in normotensive men with electrocardiographically determined left ventricular hypertrophy. *Hypertension*. 2014
99. Kang, D.H., et al. Early surgery versus conventional treatment for asymptomatic severe mitral regurgitation: a propensity analysis. *J Am Coll Cardiol*. 2014
100. Kariyawasam, V.C., et al. Early use of thiopurines or methotrexate reduces major abdominal and perianal surgery in Crohn's disease. *Inflamm Bowel Dis.* 2014
101. Karran, A., et al. Propensity score analysis of oesophageal cancer treatment with surgery or definitive chemoradiotherapy. *Br J Surg.* 2014
102. Kasama, S., et al. Effects of oral nicorandil therapy on sympathetic nerve activity and cardiac events in patients with chronic heart failure: subanalysis of our previous report using propensity score matching. *Eur J Nucl Med Mol Imaging*. 2014
103. Kashy, B.K., et al. Effect of hydroxyethyl starch on postoperative kidney function in patients having noncardiac surgery. *Anesthesiology.* 2014
104. Keating, N.L., et al. Androgen-deprivation therapy and diabetes control among diabetic men with prostate cancer. *Eur Urol.* 2014
105. Keenan, B.T., et al. Obstructive sleep apnoea treatment and fasting lipids: a comparative effectiveness study. *Eur Respir J.* 2014
106. Kemp, A.H., et al. Effects of depression, anxiety, comorbidity, and antidepressants on resting-state heart rate and its variability: an ELSA-Brasil cohort baseline study. *Am J Psychiatry*. 2014
107. Khera, S., et al. Management and outcomes of ST-elevation myocardial infarction in nursing home versus community-dwelling older patients: a propensity matched study. *J Am Med Dir Assoc*. 2014
108. Kilic, S., et al. The effect of thrombus aspiration during primary percutaneous coronary intervention on clinical outcome in daily clinical practice*. Thromb Haemost.* 2014
109. Kim, E.K., et al. A statewide colectomy experience: the role of full bowel preparation in preventing surgical site infection. *Ann Surg*. 2014
110. Kim, G.A., et al. HBsAg seroclearance after nucleoside analogue therapy in patients with chronic hepatitis B: clinical outcomes and durability. *Gut*. 2014
111. Kim, H., et al. A population-based approach indicates an overall higher patient mortality with peritoneal dialysis compared to hemodialysis in Korea. *Kidney Int*. 2014
112. Kim, H.H., et al. Long-term results of laparoscopic gastrectomy for gastric cancer: a large-scale case-control and case-matched Korean multicenter study. *J Clin Oncol.* 2014
113. Kim, J.S., et al. Long-term outcomes of neointimal hyperplasia without neoatherosclerosis after drug-eluting stent implantation. *JACC Cardiovasc Imaging.* 2014
114. Kim, L.K., et al. Comparison of trends and outcomes of carotid artery stenting and endarterectomy in the United States, 2001 to 2010*. Circ Cardiovasc Interv.* 2014
115. Kim, S.J., et al. An optimal transition time to extracorporeal cardiopulmonary resuscitation for predicting good neurological outcome in patients with out-of-hospital cardiac arrest: a propensity-matched study Crit Care 2014
116. Kincaid, D.L., et al. HIV communication programs, condom use at sexual debut, and HIV infections averted in South Africa, 2005. *J Acquir Immune Defic Syndr.* 2014
117. Kiser, T.H., et al. Outcomes associated with corticosteroid dosage in critically ill patients with acute exacerbations of chronic obstructive pulmonary disease*. Am J Respir Crit Care Med.* 2014
118. Koivunen, R.J., et al. Predictors of early mortality in young adults after intracerebral hemorrhage Stroke 2014
119. Kooiman, J., et al. Risk of acute kidney injury after percutaneous coronary interventions using radial versus femoral vascular access: insights from the Blue Cross Blue Shield of Michigan Cardiovascular Consortium. *Circ Cardiovasc Interv*. 2014
120. Kovesdy, C.P., et al. Observational modeling of strict vs conventional blood pressure control in patients with chronic kidney disease. *JAMA Intern Med.* 2014
121. Kranz, A.M., et al. Preventive Services by Medical and Dental Providers and Treatment Outcomes*. J Dent Res.* 2014
122. Kumar, R.K., et al. Robot-assisted partial nephrectomy in patients with baseline chronic kidney disease: a multi-institutional propensity score-matched analysis. *Eur Urol.* 2014
123. Kumar, V.A., et al. Survival of propensity matched incident peritoneal and hemodialysis patients in a United States health care system. *Kidney Int.* 2014
124. Kuo, S.C., et al. Association between recent use of fluoroquinolones and rhegmatogenous retinal detachment: a population-based cohort study. *Clin Infect Dis*. 2014
125. Kuragano, T., et al. Association between hemoglobin variability, serum ferritin levels, and adverse events/mortality in maintenance hemodialysis patients. *Kidney Int.* 2014
126. Kutup, A., et al. What should be the gold standard for the surgical component in the treatment of locally advanced esophageal cancer: transthoracic versus transhiatal esophagectomy. *Ann Surg*. 2014
127. Kwon, Y., et al. The foregut theory as a possible mechanism of action for the remission of type 2 diabetes in low body mass index patients undergoing subtotal gastrectomy for gastric cancer. *Surg Obes Relat Dis*. 2014
128. Landreneau, R.J., et al. Recurrence and survival outcomes after anatomic segmentectomy versus lobectomy for clinical stage I non-small-cell lung cancer: a propensity-matched analysis. *J Clin Oncol.* 2014
129. Lavallee, P.C., et al. Influenza vaccination and cardiovascular risk in patients with recent TIA and stroke. *Neurology*. 2014
130. Layton, J.B., et al. Sodium phosphate does not increase risk for acute kidney injury after routine colonoscopy, compared with polyethylene glycol. *Clin Gastroenterol Hepatol.* 2014
131. Lee, S.J., et al. Impact of chronic obstructive pulmonary disease on the mortality of patients with non-small-cell lung cancer. *J Thorac Oncol.* 2014
132. Lee, S.J., et al. Effects of pivoting neuromuscular training on pivoting control and proprioception. *Med Sci Sports Exerc*. 2014
133. Lee, Y.G., et al. Risk factors and prognostic impact of venous thromboembolism in Asian patients with non-small cell lung cancer. *Thromb Haemost.* 2014
134. Leipsic, J., et al. Sex-based prognostic implications of nonobstructive coronary artery disease: results from the international multicenter CONFIRM study. *Radiology.* 2014
135. Leow, J.J., et al. Propensity-matched comparison of morbidity and costs of open and robot-assisted radical cystectomies: a contemporary population-based analysis in the United States. *Eur Urol.* 2014
136. Levin, M.A., et al. Low intraoperative tidal volume ventilation with minimal PEEP is associated with increased mortality. *Br J Anaesth.* 2014
137. Lewinter, C., et al. Impact of aspirin and statins on long-term survival in patients hospitalized with acute myocardial infarction complicated by heart failure: an analysis of 1706 patients. *Eur J Heart Fail.* 2014
138. Li, Y., et al. Sulfonylurea use and incident cardiovascular disease among patients with type 2 diabetes: prospective cohort study among women. *Diabetes Care*. 2014
139. Liang, J.J., et al. Outcomes after percutaneous coronary intervention with stents in patients treated with thoracic external beam radiation for cancer. *JACC Cardiovasc Interv.* 2014
140. Liao, C.C., et al. Outcomes after surgery in patients with previous stroke. *Br J Surg*. 2014
141. Liddle, A.D., et al. Adverse outcomes after total and unicompartmental knee replacement in 101,330 matched patients: a study of data from the National Joint Registry for England and Wales. *Lancet*. 2014
142. Lilly, C.M., et al. Thrombosis prophylaxis and mortality risk among critically ill adults. *Chest.* 2014
143. Lim, Y.S., et al. Mortality, liver transplantation, and hepatocellular carcinoma among patients with chronic hepatitis B treated with entecavir vs lamivudine. *Gastroenterology.* 2014
144. Lin, T.Y., et al. Association between chronic osteomyelitis and deep-vein thrombosis. Analysis of a nationwide population-based registry. *Thromb Haemost.* 2014
145. Linder, A., et al. Small acute increases in serum creatinine are associated with decreased long-term survival in the critically ill. *Am J Respir Crit Care Med.* 2014
146. Lipitz-Snyderman, A., et al. Long-term central venous catheter use and risk of infection in older adults with cancer*. J Clin Oncol.* 2014
147. Liu, C.J., et al. Treatment of patients with dual hepatitis C and B by peginterferon alpha and ribavirin reduced risk of hepatocellular carcinoma and mortality. *Gut.* 2014
148. Liu, J., et al. Serum free fatty acid biomarkers of lung cancer. *Chest.* 2014
149. Lonardo, N.W., et al. Propofol is associated with favorable outcomes compared with benzodiazepines in ventilated intensive care unit patients. *Am J Respir Crit Care Med.* 2014
150. Lopez-Cortes, L.E., et al. Monotherapy versus combination therapy for sepsis due to multidrug-resistant Acinetobacter baumannii: analysis of a multicentre prospective cohort. *J Antimicrob Chemother.* 2014
151. Lucas, D.J., et al. Interhospital transfer and adverse outcomes after general surgery: implications for pay for performance. *J Am Coll Surg*. 2014
152. MacLaren, R., et al. Histamine-2 receptor antagonists vs proton pump inhibitors on gastrointestinal tract hemorrhage and infectious complications in the intensive care unit. *JAMA Intern Med*. 2014
153. Main, M.L., et al. Acute mortality in critically ill patients undergoing echocardiography with or without an ultrasound contrast agent. *JACC Cardiovasc Imaging*. 2014
154. Makam, A.N., et al. Risk of thiazide-induced metabolic adverse events in older adults. *J Am Geriatr Soc.* 2014
155. Marcum, Z.A., et al. Effect of multiple pharmacy use on medication adherence and drug-drug interactions in older adults with Medicare Part D*. J Am Geriatr Soc*. 2014
156. Marra, F., et al. Effectiveness of neuraminidase inhibitors in preventing hospitalization during the H1N1 influenza pandemic in British Columbia, Canada. J Antimicrob Chemother. 2014
157. Martin, C.T., et al. Thirty-Day Morbidity After Single-Level Anterior Cervical Discectomy and Fusion: Identification of Risk Factors and Emphasis on the Safety of Outpatient Procedures. *J Bone Joint Surg Am.* 2014
158. Masoudi, F.A., et al. Comparative effectiveness of cardiac resynchronization therapy with an implantable cardioverter-defibrillator versus defibrillator therapy alone: a cohort study. *Ann Intern Med*. 2014
159. Mathew, J.G., et al. Efficacy and safety of early parenteral anticoagulation as a bridge to warfarin after mechanical valve replacement. *Thromb Haemost.* 2014
160. McCutcheon, B.A., et al. Long-term outcomes of patients with nonsurgically managed uncomplicated appendicitis. *J Am Coll Surg*. 2014
161. McDonald, J.S., et al. Risk of intravenous contrast material-mediated acute kidney injury: a propensity score-matched study stratified by baseline-estimated glomerular filtration rate. *Radiology.* 2014
162. McDonald, R.J., et al. Intravenous contrast material exposure is not an independent risk factor for dialysis or mortality. *Radiology.* 2014
163. Metcalfe, K., et al. Contralateral mastectomy and survival after breast cancer in carriers of BRCA1 and BRCA2 mutations: retrospective analysis. *Bmj*. 2014
164. Migita, K., et al. Venous thromboembolism after total joint arthroplasty: results from a Japanese multicenter cohort study. *Arthritis Res Ther*. 2014
165. Miller, M., et al. Antidepressant dose, age, and the risk of deliberate self-harm. *JAMA Intern Med.* 2014
166. Min, Y.W., et al. Proton pump inhibitor use significantly increases the risk of spontaneous bacterial peritonitis in 1965 patients with cirrhosis and ascites: a propensity score matched cohort study. *Aliment Pharmacol Ther*. 2014
167. Molto, A., et al. Effectiveness of tumor necrosis factor alpha blockers in early axial spondyloarthritis: data from the DESIR cohort. *Arthritis Rheumatol.* 2014
168. Mortara, A., et al. Treatment with inotropes and related prognosis in acute heart failure: contemporary data from the Italian Network on Heart Failure (IN-HF) Outcome registry. *J Heart Lung Transplant.* 2014
169. Mortensen, J.K., et al. Impact of prestroke selective serotonin reuptake inhibitor treatment on stroke severity and mortality. *Stroke.* 2014
170. Muriel, A., et al. Survival effects of inferior vena cava filter in patients with acute symptomatic venous thromboembolism and a significant bleeding risk. *J Am Coll Cardiol.* 2014
171. Nagami, Y., et al. Usefulness of non-magnifying narrow-band imaging in screening of early esophageal squamous cell carcinoma: a prospective comparative study using propensity score matching. *Am J Gastroenterol.* 2014
172. Nanda, A., et al. Neoadjuvant hormonal therapy use and the risk of death in men with prostate cancer treated with brachytherapy who have no or at least a single risk factor for coronary artery disease. *Eur Urol.* 2014
173. Nielsen, D.V., et al. Health outcomes with and without use of inotropic therapy in cardiac surgery: results of a propensity score-matched analysis. *Anesthesiology.* 2014
174. Nutman, A., et al. A case-control study to identify predictors of 14-day mortality following carbapenem-resistant Acinetobacter baumannii bacteraemia. *Clin Microbiol Infect*. 2014
175. O'Brien, D.D., et al. Intraoperative risk factors associated with postoperative pressure ulcers in critically ill patients: a retrospective observational study. *Crit Care Med.* 2014
176. Oh, H.J., et al. The benefit of specialized team approaches in patients with acute kidney injury undergoing continuous renal replacement therapy: propensity score matched analysis. *Crit Care*. 2014
177. Ohinmaa, A.E., et al. Canadian estimates of health care utilization costs for rheumatoid arthritis patients with and without therapy with biologic agents. *Arthritis Care Res (Hoboken).* 2014
178. Ortiz, H., et al. Multicentre propensity score-matched analysis of conventional versus extended abdominoperineal excision for low rectal cancer. *Br J Surg.* 2014
179. Osterman, M.T., et al. Comparative effectiveness of infliximab and adalimumab for Crohn's disease. *Clin Gastroenterol Hepatol.* 2014
180. Ou, S.Y., et al. Effect of the use of low and high potency statins and sepsis outcomes. *Intensive Care Med.* 2014
181. Page, V.J., et al. Statin use and risk of delirium in the critically ill. *Am J Respir Crit Care Med.* 2014
182. Parikh, N.D., et al. Inpatient weekend ERCP is associated with a reduction in patient length of stay. *Am J Gastroenterol.* 2014
183. Park, K.W., et al. Everolimus-eluting Xience v/Promus versus zotarolimus-eluting resolute stents in patients with diabetes mellitus. *JACC Cardiovasc Interv*. 2014
184. Park, S.H., et al. The efficacy of non-carbapenem antibiotics for the treatment of community-onset acute pyelonephritis due to extended-spectrum beta-lactamase-producing Escherichia coli. *J Antimicrob Chemother*. 2014
185. Park, S.H., et al. Short versus prolonged courses of antibiotic therapy for children with uncomplicated Gram-negative bacteraemia. *J Antimicrob Chemother*. 2014
186. Pasternak, B., et al. Association of treatment with carvedilol vs metoprolol succinate and mortality in patients with heart failure*. JAMA Intern Med.* 2014
187. Paul, S., et al. Long term survival with thoracoscopic versus open lobectomy: propensity matched comparative analysis using SEER-Medicare database*. Bmj.* 2014
188. Perel, P., et al. Red blood cell transfusion and mortality in trauma patients: risk-stratified analysis of an observational study. *PLoS Med.* 2014
189. Planer, D., et al. Prognosis of patients with non-ST-segment-elevation myocardial infarction and nonobstructive coronary artery disease: propensity-matched analysis from the Acute Catheterization and Urgent Intervention Triage Strategy trial. *Circ Cardiovasc Interv.* 2014
190. Poddar, K.L., et al. Risk of cerebrovascular events in patients with patent foramen ovale and intracardiac devices. *JACC Cardiovasc Interv*. 2014
191. Potosky, A.L., et al. Effectiveness of primary androgen-deprivation therapy for clinically localized prostate cancer. *J Clin Oncol*. 2014
192. Queen, M.A., et al. Comparative effectiveness of empiric antibiotics for community-acquired pneumonia. *Pediatrics*. 2014
193. Raghunathan, K., et al. Association between the choice of IV crystalloid and in-hospital mortality among critically ill adults with sepsis*. *Crit Care Med*. 2014
194. Rasmussen, J.B., et al. Relative efficacy of cefuroxime versus dicloxacillin as definitive antimicrobial therapy in methicillin-susceptible Staphylococcus aureus bacteraemia: a propensity-score adjusted retrospective cohort study*. J Antimicrob Chemother.* 2014
195. Ravi, B., et al. Relation between surgeon volume and risk of complications after total hip arthroplasty: propensity score matched cohort study. *Bmj*. 2014
196. Rawat, R., et al. The impact of a food assistance program on nutritional status, disease progression, and food security among people living with HIV in Uganda. *J Acquir Immune Defic Syndr*. 2014
197. Rawlings, A.M., et al. Diabetes in midlife and cognitive change over 20 years: a cohort study. *Ann Intern Med.* 2014
198. Rice, J.B., et al. Burden of diabetic foot ulcers for medicare and private insurers. *Diabetes Care.* 2014
199. Ritzwoller, D.P., et al. Comparative effectiveness of adjunctive bevacizumab for advanced lung cancer: the cancer research network experience*. J Thorac Oncol.* 2014
200. Robertson, J.O., et al. Concomitant tricuspid valve surgery during implantation of continuous-flow left ventricular assist devices: a Society of Thoracic Surgeons database analysis*. J Heart Lung Transplant.* 2014
201. Robinson, W.R., et al. Childbearing is not associated with young women's long-term obesity risk. *Obesity (Silver Spring).* 2014
202. Rudolph, J.L., et al. A delirium risk modification program is associated with hospital outcomes. *J Am Med Dir Assoc*. 2014
203. Saleh, A., et al. Allogenic blood transfusion following total hip arthroplasty: results from the nationwide inpatient sample, 2000 to 2009*. J Bone Joint Surg Am*. 2014
204. Salisbury, A.C., et al. Blood transfusion during acute myocardial infarction: association with mortality and variability across hospitals. *J Am Coll Cardiol.* 2014
205. Sanford, D.E., et al. Association of discharge home with home health care and 30-day readmission after pancreatectomy*. J Am Coll Surg*. 2014
206. Santana-Davila, R., et al. Cisplatin versus carboplatin-based regimens for the treatment of patients with metastatic lung cancer. An analysis of Veterans Health Administration data. *J Thorac Oncol.* 2014
207. Schmidt, M., et al. Impact of fluid balance on outcome of adult patients treated with extracorporeal membrane oxygenation. *Intensive Care Med.* 2014
208. Schmidt, M., et al. Preadmission use of nonaspirin nonsteroidal anti-inflammatory drugs and 30-day stroke mortality. *Neurology.* 2014
209. Schmitt, S., et al. Infectious diseases specialty intervention is associated with decreased mortality and lower healthcare costs. *Clin Infect Dis.* 2014
210. Schmitz, M.L., et al. Acute ischemic stroke and long-term outcome after thrombolysis: nationwide propensity score-matched follow-up study. *Stroke*. 2014
211. Seitz, D.P., et al. Postoperative medical complications associated with anesthesia in older adults with dementia. *J Am Geriatr Soc.* 2014
212. Seow, H., et al. Impact of community based, specialist palliative care teams on hospitalisations and emergency department visits late in life and hospital deaths: a pooled analysis. *Bmj.* 2014
213. Shah, M., et al. Warfarin use and the risk for stroke and bleeding in patients with atrial fibrillation undergoing dialysis. *Circulation*. 2014
214. Shao, Y.H., et al. Cancer-specific survival after metastasis following primary radical prostatectomy compared with radiation therapy in prostate cancer patients: results of a population-based, propensity score-matched analysis. *Eur Urol.* 2014
215. Shinzawa, M., et al. Comparison of methylprednisolone plus prednisolone with prednisolone alone as initial treatment in adult-onset minimal change disease: a retrospective cohort study. *Clin J Am Soc Nephrol.* 2014
216. Shreibati, J.B., et al. Outcomes after coronary artery calcium and other cardiovascular biomarker testing among asymptomatic medicare beneficiaries. *Circ Cardiovasc Imaging*. 2014
217. Shrestha, N.K., et al. Adverse events, healthcare interventions and healthcare utilization during home infusion therapy with daptomycin and vancomycin: a propensity score-matched cohort study. *J Antimicrob Chemother.* 2014
218. Sick, A.C., et al. Empiric combination therapy for gram-negative bacteremia. *Pediatrics.* 2014
219. Siedner, M.J., et al. Cefepime vs other antibacterial agents for the treatment of Enterobacter species bacteremia. *Clin Infect Dis.* 2014
220. Silverman, D., et al. Skin involvement and breast cancer: are T4b lesions of all sizes created equal? *J Am Coll Surg.* 2014
221. Simonetti, A.F., et al. Impact of pre-hospital antibiotic use on community-acquired pneumonia. *Clin Microbiol Infect*. 2014
222. Sloan, F.A.; Hanrahan, B.W. Cost offsets to medicare attributable to receipt of hip, knee, and shoulder arthroplasty. *Arthritis Care Res (Hoboken).* 2014
223. Smith, C.B., et al. Comparative outcomes of elderly stage I lung cancer patients treated with segmentectomy via video-assisted thoracoscopic surgery versus open resection. *J Thorac Oncol.* 2014
224. Solomon, M.D., et al. Comparative effectiveness of clopidogrel in medically managed patients with unstable angina and non-ST-segment elevation myocardial infarction. *J Am Coll Cardiol*. 2014
225. Sommers, B.D., et al. Changes in mortality after Massachusetts health care reform: a quasi-experimental study. *Ann Intern Med.* 2014
226. Sooriakumaran, P., et al. Comparative effectiveness of radical prostatectomy and radiotherapy in prostate cancer: observational study of mortality outcomes. *Bmj.* 2014
227. Sooriakumaran, P., et al. A multinational, multi-institutional study comparing positive surgical margin rates among 22393 open, laparoscopic, and robot-assisted radical prostatectomy patients. *Eur Urol.* 2014
228. Sorbets, E., et al. Renin-angiotensin system antagonists and clinical outcomes in stable coronary artery disease without heart failure. *Eur Heart J*. 2014
229. Speicher, P.J., et al. Survival in the elderly after pneumonectomy for early-stage non-small cell lung cancer: a comparison with nonoperative management. *J Am Coll Surg*. 2014
230. Steingrub, J.S., et al. Treatment with neuromuscular blocking agents and the risk of in-hospital mortality among mechanically ventilated patients with severe sepsis. *Crit Care Med.* 2014
231. Svanstrom, H., et al. Use of clarithromycin and roxithromycin and risk of cardiac death: cohort study. *Bmj*. 2014
232. Swaans, M.J., et al. Survival of transcatheter mitral valve repair compared with surgical and conservative treatment in high-surgical-risk patients. *JACC Cardiovasc Interv.* 2014
233. Tagami, T., et al. Antithrombin and mortality in severe pneumonia patients with sepsis-associated disseminated intravascular coagulation: an observational nationwide study. *J Thromb Haemost*. 2014
234. Tajeu, G.S., et al. Death, debility, and destitution following hip fracture*. J Gerontol A Biol Sci Med Sci.* 2014
235. Taricco, M., et al. Impact of adapted physical activity and therapeutic patient education on functioning and quality of life in patients with postacute strokes. *Neurorehabil Neural Repair.* 2014
236. Tate, J.A., et al. Infection hospitalization increases risk of dementia in the elderly. *Crit Care Med.* 2014
237. Thompson, C.M., et al. Traumatic injury, early gene expression, and gram-negative bacteremia. *Crit Care Med.* 2014
238. Tinetti, M.E., et al. Antihypertensive medications and serious fall injuries in a nationally representative sample of older adults. *JAMA Intern Med.* 2014
239. Trotta, F., et al. Evaluation of safety of A/H1N1 pandemic vaccination during pregnancy: cohort study. *Bmj*. 2014
240. Turakhia, M.P., et al. Increased mortality associated with digoxin in contemporary patients with atrial fibrillation: findings from the TREAT-AF study*. J Am Coll Cardiol.* 2014
241. Turpie, A.G., et al. A non-interventional comparison of rivaroxaban with standard of care for thromboprophylaxis after major orthopaedic surgery in 17,701 patients with propensity score adjustment. *Thromb Haemost*. 2014
242. Tweet, M.S., et al. Spontaneous coronary artery dissection: revascularization versus conservative therapy. *Circ Cardiovasc Interv*. 2014
243. Vaara, S.T., et al. The attributable mortality of acute kidney injury: a sequentially matched analysis*. *Crit Care Med.* 2014
244. van Leersum, N., et al. Differences in circumferential resection margin involvement after abdominoperineal excision and low anterior resection no longer significant. *Ann Surg.* 2014
245. Vogel, R.I., et al. Exposure to indoor tanning without burning and melanoma risk by sunburn history*. J Natl Cancer Inst*. 2014
246. Vozoris, N.T., et al. Benzodiazepine drug use and adverse respiratory outcomes among older adults with COPD. *Eur Respir J*. 2014
247. Wachtel, H., et al. Primary hyperparathyroidism with negative imaging: a significant clinical problem. *Ann Surg.* 2014
248. Wagner, C.E., et al. Etomidate use and postoperative outcomes among cardiac surgery patients. *Anesthesiology.* 2014
249. Wald, R., et al. The association between renal replacement therapy modality and long-term outcomes among critically ill adults with acute kidney injury: a retrospective cohort study*. *Crit Care Med.* 2014
250. Walters, T.D., et al. Increased effectiveness of early therapy with anti-tumor necrosis factor-alpha vs an immunomodulator in children with Crohn's disease. *Gastroenterology.* 2014
251. Wang, H.H., et al. Risk of stroke in long-term dialysis patients compared with the general population. *Am J Kidney Dis*. 2014
252. Warschkow, R., et al. Perioperative blood transfusions do not impact overall and disease-free survival after curative rectal cancer resection: a propensity score analysis. *Ann Surg*. 2014
253. Watanabe, T., et al. Anti-tumour necrosis factor agents reduce non-steroidal anti-inflammatory drug-induced small bowel injury in rheumatoid arthritis patients. *Gut*. 2014
254. Wee, S.L., et al. Effectiveness of a national transitional care program in reducing acute care use. *J Am Geriatr Soc*. 2014
255. Weiner, D.E., et al. Oral intradialytic nutritional supplement use and mortality in hemodialysis patients*. Am J Kidney Dis.* 2014
256. Weiss, S.L., et al. Delayed antimicrobial therapy increases mortality and organ dysfunction duration in pediatric sepsis. *Crit Care Med*. 2014
257. Weston, A., et al. The efficacy of daptomycin versus vancomycin for methicillin-resistant Staphylococcus aureus bloodstream infection in patients with impaired renal function*. Clin Infect Dis*. 2014
258. Williamson, C., et al. Family history of premature cardiovascular disease: blood pressure control and long-term mortality outcomes in hypertensive patients. *Eur Heart J.* 2014
259. Willingham, M., et al. Association between intraoperative electroencephalographic suppression and postoperative mortality*. Br J Anaesth*. 2014
260. Wilson, F.P., et al. Dialysis versus nondialysis in patients with AKI: a propensity-matched cohort study. *Clin J Am Soc Nephrol*. 2014
261. Woelk, J.L., et al. Cost differences among robotic, vaginal, and abdominal hysterectomy. *Obstet Gynecol.* 2014
262. Wright, A.A., et al. Associations between palliative chemotherapy and adult cancer patients' end of life care and place of death: prospective cohort study. *Bmj.* 2014
263. Wright, J.D., et al. Comparative effectiveness of robotically assisted compared with laparoscopic adnexal surgery for benign gynecologic disease. *Obstet Gynecol.* 2014
264. Yang, J.H., et al. Percutaneous coronary intervention for nonculprit vessels in cardiogenic shock complicating ST-segment elevation acute myocardial infarction*. Crit Care Med*. 2014
265. Yang, J.H., et al. Angiotensin receptor blocker in patients with ST segment elevation myocardial infarction with preserved left ventricular systolic function: prospective cohort study. *Bmj.* 2014
266. Yang, J.H., et al. Association of beta-blocker therapy at discharge with clinical outcomes in patients with ST-segment elevation myocardial infarction undergoing primary percutaneous coronary intervention. *JACC Cardiovasc Interv*. 2014
267. Yang, N.H., et al. Videoconferencing to reduce stress among hospitalized children. *Pediatrics.* 2014
268. Yende, S., et al. Risk of cardiovascular events in survivors of severe sepsis. *Am J Respir Crit Care Med.* 2014
269. Yoshii, S., et al. Factors associated with risk for colorectal cancer recurrence after endoscopic resection of T1 tumors. *Clin Gastroenterol Hepatol*. 2014
270. Yu, P.J., et al. Propensity-matched analysis of the effect of preoperative intraaortic balloon pump in coronary artery bypass grafting after recent acute myocardial infarction on postoperative outcomes. *Crit Care.* 2014
271. Yusuf, A.A., et al. Comparative effectiveness of calcium acetate and sevelamer on clinical outcomes in elderly hemodialysis patients enrolled in Medicare part D*. Am J Kidney Dis*. 2014
272. Zhong, J.H., et al. Hepatic resection associated with good survival for selected patients with intermediate and advanced-stage hepatocellular carcinoma. *Ann Surg*. 2014
273. Kelly, K.N., et al. Disease severity, not operative approach, drives organ space infection after pediatric appendectomy. *Ann Surg*. 2014
274. Naganuma, T., et al. Long-term clinical outcomes after percutaneous coronary intervention versus coronary artery bypass grafting for ostial/midshaft lesions in unprotected left main coronary artery from the DELTA registry: a multicenter registry evaluating percutaneous coronary intervention versus coronary artery bypass grafting for left main treatment. *JACC Cardiovasc Interv*. 2014
275. Abdelsattar, Z.M., et al. Variation in Transfusion Practices and the Effect on Outcomes After Noncardiac Surgery. *Ann Surg*. 2015
276. Abraham, N.S., et al. Comparative risk of gastrointestinal bleeding with dabigatran, rivaroxaban, and warfarin: population based cohort study. *Bmj.* 2015
277. Adam, R., et al. Improved survival in liver transplant recipients receiving prolonged-release tacrolimus in the European Liver Transplant Registry. *Am J Transplant.* 2015
278. Adeoye, O., et al. Recombinant tissue-type plasminogen activator plus eptifibatide versus recombinant tissue-type plasminogen activator alone in acute ischemic stroke: propensity score-matched post hoc analysis. *Stroke.* 2015
279. Aldrich, N., et al. Genetic vs Environmental Factors That Correlate With Rosacea: A Cohort-Based Survey of Twins*. JAMA Dermatol*. 2015
280. Al-Holou, S.N., et al. The Association of Statin Use with Age-Related Macular Degeneration Progression: The Age-Related Eye Disease Study 2 Report Number 9. *Ophthalmology*. 2015
281. Alkhouli, M., et al. Comparative outcomes of catheter-directed thrombolysis plus anticoagulation versus anticoagulation alone in the treatment of inferior vena caval thrombosis. *Circ Cardiovasc Interv*. 2015
282. Allard, M.A., et al. Early and Long-term Oncological Outcomes After Laparoscopic Resection for Colorectal Liver Metastases: A Propensity Score-based Analysis. *Ann Surg*. 2015
283. Alperovitch, A., et al. Primary prevention with lipid lowering drugs and long term risk of vascular events in older people: population based cohort study. *Bmj*. 2015
284. Andria, N., et al. Mortality burden related to infection with carbapenem-resistant Gram-negative bacteria among haematological cancer patients: a retrospective cohort study. *J Antimicrob Chemother*. 2015
285. Antun, A., et al. Inhibitor recurrence after immune tolerance induction: a multicenter retrospective cohort study. *J Thromb Haemost.* 2015
286. Armstrong, A.W., et al. Psoriasis and risk of diabetes-associated microvascular and macrovascular complications. *J Am Acad Dermatol.* 2015
287. Arriola, C.S., et al. Does Influenza Vaccination Modify Influenza Severity? Data on Older Adults Hospitalized With Influenza During the 2012-2013 Season in the United States. *J Infect Dis.* 2015
288. Asai, S., et al. Effects of Concomitant Methotrexate on Large Joint Replacement in Patients With Rheumatoid Arthritis Treated With Tumor Necrosis Factor Inhibitors: A Multicenter Retrospective Cohort Study in Japan. *Arthritis Care Res (Hoboken).* 2015
289. Auerbach, S.R., et al. Maintenance steroid use at 30 days post-transplant and outcomes of pediatric heart transplantation: A propensity matched analysis of the Pediatric Heart Transplant Study database. *J Heart Lung Transplant*. 2015
290. Autmizguine, J., et al. Anaerobic antimicrobial therapy after necrotizing enterocolitis in VLBW infants. *Pediatrics.* 2015
291. Bai, A.D., et al. Impact of Infectious Disease Consultation on Quality of Care, Mortality, and Length of Stay in Staphylococcus aureus Bacteremia: Results From a Large Multicenter Cohort Study. *Clin Infect Dis*. 2015
292. Bai, A.D., et al. Comparative effectiveness of cefazolin versus cloxacillin as definitive antibiotic therapy for MSSA bacteraemia: results from a large multicentre cohort study. *J Antimicrob Chemother.* 2015
293. Bandyopadhyay, D., et al. Outcomes of beta-blocker use in pulmonary arterial hypertension: a propensity-matched analysis*. Eur Respir J*. 2015
294. Bangalore, S., et al. Revascularization in Patients With Multivessel Coronary Artery Disease and Chronic Kidney Disease: Everolimus-Eluting Stents Versus Coronary Artery Bypass Graft Surgery*. J Am Coll Cardiol.* 2015
295. Bangalore, S., et al. Everolimus Eluting Stents Versus Coronary Artery Bypass Graft Surgery for Patients With Diabetes Mellitus and Multivessel Disease. *Circ Cardiovasc Interv*. 2015
296. Bangalore, S., et al. Everolimus-eluting stents or bypass surgery for multivessel coronary disease. *N Engl J Med.* 2015
297. Barbash, I.M., et al. Comparison of vascular closure devices for access site closure after transfemoral aortic valve implantation. *Eur Heart J.* 2015
298. Bateman, B.T., et al. Statins and congenital malformations: cohort study*. Bmj.* 2015
299. Bateman, B.T., et al. Calcium Channel Blocker Exposure in Late Pregnancy and the Risk of Neonatal Seizures. *Obstet Gynecol*. 2015
300. Bekelis, K., et al. Prehospital helicopter transport and survival of patients with traumatic brain injury. *Ann Surg.* 2015
301. Bekelman, J.E., et al. Effectiveness of androgen-deprivation therapy and radiotherapy for older men with locally advanced prostate cancer. *J Clin Oncol.* 2015
302. Benetos, A., et al. Treatment With Multiple Blood Pressure Medications, Achieved Blood Pressure, and Mortality in Older Nursing Home Residents: The PARTAGE Study. *JAMA Intern Med.* 2015
303. Ben-Gal, Y., et al. Surgical versus percutaneous coronary revascularization for multivessel disease in diabetic patients with non-ST-segment-elevation acute coronary syndrome: analysis from the Acute Catheterization and Early Intervention Triage Strategy trial. *Circ Cardiovasc Interv.* 2015
304. Berger, L.M., et al. Home Foreclosure and Child Protective Services Involvement. *Pediatrics.* 2015
305. Berry, D.L., et al. Exposure to a patient-centered, Web-based intervention for managing cancer symptom and quality of life issues: impact on symptom distress. *J Med Internet Res.* 2015
306. Bertelsen, C.A., et al. Disease-free survival after complete mesocolic excision compared with conventional colon cancer surgery: a retrospective, population-based study. *Lancet Oncol.* 2015
307. Bhavan, K.P., et al. Self-Administered Outpatient Antimicrobial Infusion by Uninsured Patients Discharged from a Safety-Net Hospital: A Propensity-Score-Balanced Retrospective Cohort Study. *PLoS Med.* 2015
308. Bige, N., et al. Homeless Patients in the ICU: An Observational Propensity-Matched Cohort Study. *Crit Care Med.* 2015
309. Blackstone, E.H., et al. Propensity-matched comparisons of clinical outcomes after transapical or transfemoral transcatheter aortic valve replacement: a placement of aortic transcatheter valves (PARTNER)-I trial substudy. *Circulation*. 2015
310. Boshuisen, K., et al. Intelligence quotient improves after antiepileptic drug withdrawal following pediatric epilepsy surgery. *Ann Neurol*. 2015
311. Brennan, J.M., et al. Three-year outcomes associated with embolic protection in saphenous vein graft intervention: results in 49 325 senior patients in the Medicare-linked National Cardiovascular Data Registry CathPCI Registry. *Circ Cardiovasc Interv.* 2015
312. Bristow, R.E., et al. Sociodemographic disparities in advanced ovarian cancer survival and adherence to treatment guidelines. *Obstet Gynecol.* 2015
313. Brown, J.B., et al. Pretrauma center red blood cell transfusion is associated with reduced mortality and coagulopathy in severely injured patients with blunt trauma. *Ann Surg*. 2015
314. Brown, J.B., et al. Pre-trauma center red blood cell transfusion is associated with improved early outcomes in air medical trauma patients. *J Am Coll Surg.* 2015
315. Brugaletta, S., et al. Absorb bioresorbable vascular scaffold versus everolimus-eluting metallic stent in ST-segment elevation myocardial infarction: 1-year results of a propensity score matching comparison: the BVS-EXAMINATION Study (bioresorbable vascular scaffold-a clinical evaluation of everolimus eluting coronary stents in the treatment of patients with ST-segment elevation myocardial infarction). *JACC Cardiovasc Interv.* 2015
316. Callaghan, B., et al. Longitudinal patient-oriented outcomes in neuropathy: Importance of early detection and falls. *Neurology.* 2015
317. Cantor, W.J., et al. Reperfusion Times for Radial Versus Femoral Access in Patients With ST-Elevation Myocardial Infarction Undergoing Primary Percutaneous Coronary Intervention: Observations From the Cardiac Care Network Provincial Primary PCI Registry. *Circ Cardiovasc Interv*. 2015
318. Capodanno, D., et al. Impact of bridging with perioperative low-molecular-weight heparin on cardiac and bleeding outcomes of stented patients undergoing non-cardiac surgery. *Thromb Haemost*. 2015
319. Cardwell, C.R., et al. Statin use after diagnosis of breast cancer and survival: a population-based cohort study. *Epidemiology.* 2015
320. Cauchy, F., et al. Risk factors and consequences of conversion in laparoscopic major liver resection. *Br J Surg.* 2015
321. Caulley, L., et al. Direct costs of adult chronic rhinosinusitis by using 4 methods of estimation: Results of the US Medical Expenditure Panel Survey. *J Allergy Clin Immunol*. 2015
322. Chan, E.K., et al. Long-term mortality from cardiac causes after adjuvant hypofractionated vs. conventional radiotherapy for localized left-sided breast cancer. *Radiother Oncol*. 2015
323. Chan, W., et al. Clinical outcomes of treatment by percutaneous coronary intervention versus coronary artery bypass graft surgery in patients with chronic kidney disease undergoing index revascularization in Ontario. *Circ Cardiovasc Interv*. 2015
324. Chang, H.Y., et al. Risk of gastrointestinal bleeding associated with oral anticoagulants: population based retrospective cohort study. *Bmj*. 2015
325. Chen, C.M., et al. Effect of end-stage renal disease on long-term survival after a first-ever mechanical ventilation: a population-based study. *Crit Care.* 2015
326. Chen, C.Y., et al. Real world effectiveness of primary implantable cardioverter defibrillators implanted during hospital admissions for exacerbation of heart failure or other acute co-morbidities: cohort study of older patients with heart failure. *Bmj.* 2015
327. Chen, Y.C., et al. Clinical outcomes after interruption of entecavir therapy in HBeAg-negative chronic hepatitis B patients with compensated cirrhosis. *Aliment Pharmacol Ther*. 2015
328. Choi, J.C., et al. Early outcomes after carotid artery stenting compared with endarterectomy for asymptomatic carotid stenosis. *Stroke*. 2015
329. Chou, H.W., et al. Risks of cardiac arrhythmia and mortality among patients using new-generation macrolides, fluoroquinolones, and beta-lactam/beta-lactamase inhibitors: a Taiwanese nationwide study. *Clin Infect Dis.* 2015
330. Chu, C.C., et al. Propensity Score-matched Comparison of Postoperative Adverse Outcomes between Geriatric Patients Given a General or a Neuraxial Anesthetic for Hip Surgery: A Population-based Study. *Anesthesiology.* 2015
331. Chughtai, B., et al. Use and risks of surgical mesh for pelvic organ prolapse surgery in women in New York state: population based cohort study. *Bmj*. 2015
332. Cindolo, L., et al. Drug adherence and clinical outcomes for patients under pharmacological therapy for lower urinary tract symptoms related to benign prostatic hyperplasia: population-based cohort study Eur Urol 2015
333. Corso, C.D., et al. Role of Chemoradiotherapy in Elderly Patients With Limited-Stage Small-Cell Lung Cancer. *J Clin Oncol*. 2015
334. Dankiewicz, J., et al. Survival in patients without acute ST elevation after cardiac arrest and association with early coronary angiography: a post hoc analysis from the TTM trial. *Intensive Care Med.* 2015
335. de Rooij, T., et al. A nationwide comparison of laparoscopic and open distal pancreatectomy for benign and malignant disease. *J Am Coll Surg.* 2015
336. de Souza, A.R., et al. Prognosis of acute variceal bleeding: Is being on beta-blockers an aggravating factor? A short-term survival analysis. *Hepatology.* 2015
337. de Vries, H., et al. Cardiac rehabilitation and survival in a large representative community cohort of Dutch patients. *Eur Heart J.* 2015
338. Desai, R.J., et al. Disease-modifying antirheumatic drug use and the risk of incident hyperlipidemia in patients with early rheumatoid arthritis: a retrospective cohort study. *Arthritis Care Res (Hoboken).* 2015
339. Desai, R.J., et al. Exposure to prescription opioid analgesics in utero and risk of neonatal abstinence syndrome: population based cohort study. *Bmj.* 2015
340. Dewilde, W.J., et al. The effect of acenocoumarol on the antiplatelet effect of clopidogrel Thromb Haemost 2015
341. Dimopoulos, M.A., et al. Retrospective matched-pairs analysis of bortezomib plus dexamethasone versus bortezomib monotherapy in relapsed multiple myeloma. *Haematologica*. 2015
342. Douglas, I.J., et al. Bariatric Surgery in the United Kingdom: A Cohort Study of Weight Loss and Clinical Outcomes in Routine Clinical Care. *PLoS Med.* 2015
343. Douketis, J.D., et al. Perioperative bridging anticoagulation during dabigatran or warfarin interruption among patients who had an elective surgery or procedure. Substudy of the RE-LY trial. *Thromb Haemost*. 2015
344. Du, X.L., et al. Comparative Effectiveness of Chemotherapy Regimens in Prolonging Survival for Two Large Population-Based Cohorts of Elderly Adults with Breast and Colon Cancer in 1992-2009. *J Am Geriatr Soc.* 2015
345. Dubreuil, M., et al. Allopurinol initiation and all-cause mortality in the general population*. Ann Rheum Dis*. 2015
346. Ducarme, G., et al. Maternal and Neonatal Morbidity After Attempted Operative Vaginal Delivery According to Fetal Head Station. *Obstet Gynecol.* 2015
347. Edner, M., et al. Association between renin-angiotensin system antagonist use and mortality in heart failure with severe renal insufficiency: a prospective propensity score-matched cohort study. *Eur Heart J.* 2015
348. Ensing, S., et al. Risk of poor neonatal outcome at term after medically assisted reproduction: a propensity score-matched study. *Fertil Steril.* 2015
349. Epstein, A.J., et al. Adjuvant Chemotherapy Use and Health Care Costs After Introduction of Genomic Testing in Breast Cancer. *J Clin Oncol*. 2015
350. Ezer, N., et al. Outcomes after Stereotactic Body Radiotherapy versus Limited Resection in Older Patients with Early-Stage Lung Cancer. *J Thorac Oncol.* 2015
351. Fassl, J., et al. Perioperative administration of fibrinogen does not increase adverse cardiac and thromboembolic events after cardiac surgery. *Br J Anaesth.* 2015
352. Fawzy, A., et al. Practice Patterns and Outcomes Associated With Choice of Initial Vasopressor Therapy for Septic Shock. *Crit Care Med.* 2015
353. Fishman, E.I. Incident Diabetes and Mobility Limitations: Reducing Bias Through Risk-set Matching. *J Gerontol A Biol Sci Med Sci.* 2015
354. Fournier, J.P., et al. Tramadol use and the risk of hospitalization for hypoglycemia in patients with noncancer pain. *JAMA Intern Med.* 2015
355. Fulton, R.L., et al. Acetaminophen use and risk of myocardial infarction and stroke in a hypertensive cohort. *Hypertension*. 2015
356. Garg, S., et al. Outcomes of Percutaneous Coronary Intervention Performed at Offsite Versus Onsite Surgical Centers in the United Kingdom*. J Am Coll Cardiol*. 2015
357. Geri, G., et al. Immediate Percutaneous Coronary Intervention Is Associated With Improved Short- and Long-Term Survival After Out-of-Hospital Cardiac Arrest. *Circ Cardiovasc Interv*. 2015
358. Giri, J., et al. Proximal versus distal embolic protection for carotid artery stenting: a national cardiovascular data registry analysis. *JACC Cardiovasc Interv*. 2015
359. Gisev, N., et al. A cost-effectiveness analysis of opioid substitution therapy upon prison release in reducing mortality among people with a history of opioid dependence*. Addiction*. 2015
360. Godoy Garraza, L., et al. Effect of the Garrett Lee Smith Memorial Suicide Prevention Program on Suicide Attempts Among Youths. *JAMA Psychiatry.* 2015
361. Goldberger, J.J., et al. Effect of Beta-Blocker Dose on Survival After Acute Myocardial Infarction. *J Am Coll Cardiol*. 2015
362. Goudie, A., et al. Costs of Venous Thromboembolism, Catheter-Associated Urinary Tract Infection, and Pressure Ulcer. *Pediatrics.* 2015
363. Gozalo, P., et al. Volume Matters: Returning Home After Hip Fracture. *J Am Geriatr Soc*. 2015
364. Gozalo, P., et al. Changes in Medicare costs with the growth of hospice care in nursing homes. *N Engl J Med.* 2015
365. Graham, D.J., et al. Cardiovascular, bleeding, and mortality risks in elderly Medicare patients treated with dabigatran or warfarin for nonvalvular atrial fibrillation. *Circulation*. 2015
366. Grewal, K., et al. Missed strokes using computed tomography imaging in patients with vertigo: population-based cohort study. *Stroke.* 2015
367. Gronich, N., et al. Hypothyroidism is a Risk Factor for New-Onset Diabetes: A Cohort Study Diabetes Care 2015
368. Grunau, B.E., et al. Emergency Department Corticosteroid Use for Allergy or Anaphylaxis Is Not Associated With Decreased Relapses. *Ann Emerg Med.* 2015
369. Gurm, H.S., et al. Comparative effectiveness and safety of a catheterization laboratory-only eptifibatide dosing strategy in patients undergoing percutaneous coronary intervention. *Circ Cardiovasc Interv*. 2015
370. Habib, R.H., et al. CABG Versus PCI: Greater Benefit in Long-Term Outcomes With Multiple Arterial Bypass Grafting*. J Am Coll Cardiol*. 2015
371. Hachamovitch, R., et al. Predicting Risk Versus Predicting Potential Survival Benefit Using 123I-mIBG Imaging in Patients With Systolic Dysfunction Eligible for Implantable Cardiac Defibrillator Implantation: Analysis of Data From the Prospective ADMIRE-HF Study. *Circ Cardiovasc Imaging*. 2015
372. Hammad, T.A., et al. The Effect of Post-Exercise Ankle-Brachial Index on Lower Extremity Revascularization. *JACC Cardiovasc Interv.* 2015
373. Han, H.S., et al. Laparoscopic versus open liver resection for hepatocellular carcinoma: Case-matched study with propensity score matching*. J Hepatol*. 2015
374. Hannan, E.L., et al. Thirty-Day Readmissions After Transcatheter Aortic Valve Implantation Versus Surgical Aortic Valve Replacement in Patients With Severe Aortic Stenosis in New York State. *Circ Cardiovasc Interv.* 2015
375. Hansen, K.W., et al. Effectiveness of an early versus a conservative invasive treatment strategy in acute coronary syndromes: a nationwide cohort study. *Ann Intern Med*. 2015
376. Harrold, L.R., et al. The comparative effectiveness of abatacept versus anti-tumour necrosis factor switching for rheumatoid arthritis patients previously treated with an anti-tumour necrosis factor. *Ann Rheum Dis*. 2015
377. Harrold, L.R., et al. Comparative effectiveness and safety of rituximab versus subsequent anti-tumor necrosis factor therapy in patients with rheumatoid arthritis with prior exposure to anti-tumor necrosis factor therapies in the United States Corrona registry. *Arthritis Res Ther*. 2015
378. Harskamp, R.E., et al. Comparative Effectiveness of Hybrid Coronary Revascularization vs Coronary Artery Bypass Grafting*. J Am Coll Surg*. 2015
379. Hasegawa, K., et al. Improved management of acute asthma among pregnant women presenting to the ED. *Chest*. 2015
380. Hasselqvist-Ax, I., et al. Early cardiopulmonary resuscitation in out-of-hospital cardiac arrest. *N Engl J Med.* 2015
381. Hayes, D., Jr., et al. Influence of donor and recipient age in lung transplantation. *J Heart Lung Transplant*. 2015
382. Hayes, D., Jr., et al. Influence of age on survival in adult patients on extracorporeal membrane oxygenation before lung transplantation. *J Heart Lung Transplant.* 2015
383. Hayes, J.F., et al. The effect of sibutramine prescribing in routine clinical practice on cardiovascular outcomes: a cohort study in the United Kingdom*. Int J Obes (Lond).* 2015
384. Helwani, M.A., et al. Effects of regional versus general anesthesia on outcomes after total hip arthroplasty: a retrospective propensity-matched cohort study. *J Bone Joint Surg Am*. 2015
385. Hernandez, I., et al. Risk of bleeding with dabigatran in atrial fibrillation. *JAMA Intern Med*. 2015
386. Hong, S.J., et al. Outcomes of spot stenting versus long stenting after intentional subintimal approach for long chronic total occlusions of the femoropopliteal artery. *JACC Cardiovasc Interv.* 2015
387. Hsiang, J.C., et al. Statin and the risk of hepatocellular carcinoma and death in a hospital-based hepatitis B-infected population: A propensity score landmark analysis*. J Hepatol*. 2015
388. Hsu, C.C., et al. Use of Nonsteroidal Anti-Inflammatory Drugs and Risk of Chronic Kidney Disease in Subjects With Hypertension: Nationwide Longitudinal Cohort Study. *Hypertension.* 2015
389. Hsu, Y.C., et al. Association between antiviral treatment and extrahepatic outcomes in patients with hepatitis C virus infection. *Gut.* 2015
390. Hunt, L.J., et al. Pain in Community-Dwelling Older Adults with Dementia: Results from the National Health and Aging Trends Study. *J Am Geriatr Soc.* 2015
391. Iaffaldano, P., et al. Fingolimod versus interferon beta/glatiramer acetate after natalizumab suspension in multiple sclerosis. *Brain*. 2015
392. Ibrahim, S.L., et al. Low-dose Gentamicin for Uncomplicated Enterococcus faecalis Bacteremia May be Nephrotoxic in Children. *Clin Infect Dis*. 2015
393. Inoue, S., et al. Tracheal intubation by trainees does not alter the incidence or duration of postoperative sore throat and hoarseness: a teaching hospital-based propensity score analysis. *Br J Anaesth*. 2015
394. Iqbal, M.B., et al. Time-trend analyses of bleeding and mortality after primary percutaneous coronary intervention during out of working hours versus in-working hours: an observational study of 11 466 patients. *Circ Cardiovasc Interv.* 2015
395. Irons, D.E., et al. Tests of the effects of adolescent early alcohol exposures on adult outcomes. *Addiction*. 2015
396. Ius, F., et al. Preemptive treatment with therapeutic plasma exchange and rituximab for early donor-specific antibodies after lung transplantation. *J Heart Lung Transplant*. 2015
397. Jang, J.W., et al. Long-term effect of antiviral therapy on disease course after decompensation in patients with hepatitis B virus-related cirrhosis. *Hepatology.* 2015
398. Jang, W.J., et al. Long-term survival benefit of revascularization compared with medical therapy in patients with coronary chronic total occlusion and well-developed collateral circulation. *JACC Cardiovasc Interv*. 2015
399. Jarosek, S.L., et al. Propensity-weighted long-term risk of urinary adverse events after prostate cancer surgery, radiation, or both. *Eur Urol.* 2015
400. Jarrett, H., et al. Impact of Institutional Volume on Outcomes of Catheter Directed Thrombolysis in the Treatment of Acute Proximal Deep Vein Thrombosis: A 6-Year US Experience (2005-2010). *Circulation.* 2015
401. Jiang, L., et al. Comparison of Outcomes of Hepatic Resection and Radiofrequency Ablation for Hepatocellular Carcinoma Patients with Multifocal Tumors Meeting the Barcelona-Clinic Liver Cancer Stage A Classification. *J Am Coll Surg.* 2015
402. Jones, S.E., et al. Sarcopenia in COPD: prevalence, clinical correlates and response to pulmonary rehabilitation. *Thorax.* 2015
403. Jung, J.M., et al. Prestroke antiplatelet agents in first-ever ischemic stroke: clinical effects Neurology 2015
404. Kadakia, M.B., et al. Transradial Versus Transfemoral Access in Patients Undergoing Rescue Percutaneous Coronary Intervention After Fibrinolytic Therapy. *JACC Cardiovasc Interv.* 2015
405. Kalincik, T., et al. Switch to natalizumab versus fingolimod in active relapsing-remitting multiple sclerosis. *Ann Neurol*. 2015
406. Kamperidis, V., et al. Surgical sutureless and transcatheter aortic valves: hemodynamic performance and clinical outcomes in propensity score-matched high-risk populations with severe aortic stenosis. *JACC Cardiovasc Interv.* 2015
407. Kang, B.J., et al. Failure of high-flow nasal cannula therapy may delay intubation and increase mortality Intensive. *Care Med.* 2015
408. Kang, M., et al. Fondaparinux for the treatment of suspected heparin-induced thrombocytopenia: a propensity score-matched study. *Blood.* 2015
409. Kang, S.J., et al. Effect of obesity on coronary atherosclerosis and outcomes of percutaneous coronary intervention: grayscale and virtual histology intravascular ultrasound substudy of assessment of dual antiplatelet therapy with drug-eluting stents. *Circ Cardiovasc Interv.* 2015
410. Kang, T.W., et al. Small Hepatocellular Carcinoma: Radiofrequency Ablation versus Nonanatomic Resection--Propensity Score Analyses of Long-term Outcomes. *Radiology.* 2015
411. Kawamoto, H., et al. Impact of Strut Width in Periprocedural Myocardial Infarction: A Propensity-Matched Comparison Between Bioresorbable Scaffolds and the First-Generation Sirolimus-Eluting Stent. *JACC Cardiovasc Interv*. 2015
412. Kereiakes, D.J., et al. Stent Thrombosis in Drug-Eluting or Bare-Metal Stents in Patients Receiving Dual Antiplatelet Therapy. *JACC Cardiovasc Interv.* 2015
413. Khera, R., et al. Trends in the use of percutaneous ventricular assist devices: analysis of national inpatient sample data, 2007 through 2012*. JAMA Intern Med*. 2015
414. Khullar, O.V., et al. Survival After Sublobar Resection versus Lobectomy for Clinical Stage IA Lung Cancer: An Analysis from the National Cancer Data Base. *J Thorac Oncol.* 2015
415. Kim, H.D., et al. Evaluation of early-stage hepatocellular carcinoma by magnetic resonance imaging with gadoxetic acid detects additional lesions and increases overall survival. *Gastroenterology*. 2015
416. Kim, M.N., et al. Increased risk of hepatocellular carcinoma in chronic hepatitis B patients with transient elastography-defined subclinical cirrhosis. *Hepatology.* 2015
417. Kim, S.C., et al. Patterns of health care utilization related to initiation of amitriptyline, duloxetine, gabapentin, or pregabalin in fibromyalgia. *Arthritis Res Ther.* 2015
418. Kim, S.C., et al. Dipeptidyl peptidase-4 inhibitors in type 2 diabetes may reduce the risk of autoimmune diseases: a population-based cohort study*. Ann Rheum Dis.* 2015
419. Kim, T.H., et al. Heparin bridging in warfarin anticoagulation therapy initiation could increase bleeding in non-valvular atrial fibrillation patients: a multicenter propensity-matched analysis. *J Thromb Haemost.* 2015
420. Knox, L., et al. What Peer Mentoring Adds to Already Good Patient Care: Implementing the Carpeta Roja Peer Mentoring Program in a Well-Resourced Health Care System. *Ann Fam Med.* 2015
421. Ko, K.Y., et al. (131)I treatment for thyroid cancer and the risk of developing salivary and lacrimal gland dysfunction and a second primary malignancy: a nationwide population-based cohort study*. Eur J Nucl Med Mol Imaging*. 2015
422. Komaba, H., et al. Parathyroidectomy and survival among Japanese hemodialysis patients with secondary hyperparathyroidism. *Kidney Int.* 2015
423. Kooiman, J., et al. Association between acute kidney injury and in-hospital mortality in patients undergoing percutaneous coronary interventions. *Circ Cardiovasc Interv.* 2015
424. Koulouridis, I., et al. Hospital-acquired acute kidney injury and hospital readmission: a cohort study*. Am J Kidney Dis.* 2015
425. Kragh, J.F., Jr., et al. Transfusion for shock in US military war casualties with and without tourniquet use. *Ann Emerg Med.* 2015
426. Krishna, S.G., et al. Morbid Obesity Is Associated With Adverse Clinical Outcomes in Acute Pancreatitis: A Propensity-Matched Study. *Am J Gastroenterol.* 2015
427. Kumar, G., et al. Severe sepsis in hematopoietic stem cell transplant recipients**. Crit Care Med.* 2015
428. Kuo, Y.F., et al. Diabetes Mellitus Care Provided by Nurse Practitioners vs Primary Care Physicians. *J Am Geriatr Soc*. 2015
429. Kuritzky, A.M., et al. Lobectomy by Video-Assisted Thoracic Surgery vs Muscle-Sparing Thoracotomy for Stage I Lung Cancer: A Critical Evaluation of Short- and Long-Term Outcomes. *J Am Coll Surg.* 2015
430. Laccetti, A.L., et al. Effect of prior cancer on outcomes in advanced lung cancer: implications for clinical trial eligibility and accrual. *J Natl Cancer Inst.* 2015
431. Ladha, K., et al. Intraoperative protective mechanical ventilation and risk of postoperative respiratory complications: hospital based registry study. *Bmj*. 2015
432. Larsen, K.S., et al. Is urate crystal precipitation a predictor of cardiovascular risk in hyperuricemic patients? A Danish cohort study. *Arthritis Res Ther.* 2015
433. Lauridsen, M.D., et al. Acute kidney injury treated with renal replacement therapy and 5-year mortality after myocardial infarction-related cardiogenic shock: a nationwide population-based cohort study. *Crit Care*. 2015
434. Lauridsen, T.K., et al. Echocardiographic Findings Predict In-Hospital and 1-Year Mortality in Left-Sided Native Valve Staphylococcus aureus Endocarditis: Analysis From the International Collaboration on Endocarditis-Prospective Echo Cohort Study. *Circ Cardiovasc Imaging*. 2015
435. Laurin, L.P., et al. Outcomes of Infection-Related Hospitalization according to Dialysis Modality. *Clin J Am Soc Nephrol.* 2015
436. Le May, M.R., et al. Prophylactic warfarin therapy after primary percutaneous coronary intervention for anterior ST-segment elevation myocardial infarction. *JACC Cardiovasc Interv*. 2015
437. Lee, C.C., et al. Risk of Aortic Dissection and Aortic Aneurysm in Patients Taking Oral Fluoroquinolone*. JAMA Intern Med*. 2015
438. Lee, J.H., et al. Comparison of outcomes after laparoscopy-assisted and open total gastrectomy for early gastric cancer. *Br J Surg*. 2015
439. Lee, M.T., et al. Risk of skin ulcerations associated with oral nicorandil therapy: a population-based study. *Br J Dermatol.* 2015
440. Lee, N., et al. Neuraminidase inhibitors, superinfection and corticosteroids affect survival of influenza patients. *Eur Respir J.* 2015
441. Lee, S.M., et al. Association between Withholding Angiotensin Receptor Blockers in the Early Postoperative Period and 30-day Mortality: A Cohort Study of the Veterans Affairs Healthcare System. *Anesthesiology.* 2015
442. Lee, T.W., et al. Activities of daily living in nursing home and home care settings: a retrospective 1-year cohort study*. J Am Med Dir Assoc*. 2015
443. Lee, Y.G., et al. Implications of cytogenetics for venous thromboembolism in acute myeloid leukaemia*. Thromb Haemost.* 2015
444. Leite, T.T., et al. Renal Outcomes in Critically Ill Patients Receiving Propofol or Midazolam. *Clin J Am Soc Nephrol.* 2015
445. Leithead, J.A., et al. Non-selective beta-blockers are associated with improved survival in patients with ascites listed for liver transplantation*. Gut.* 2015
446. Leonard, C.E., et al. Comparative risk of ischemic stroke among users of clopidogrel together with individual proton pump inhibitors. *Stroke*. 2015
447. Li, X., et al. Sensitivity analysis of methods for active surveillance of acute myocardial infarction using electronic databases. *Epidemiology.* 2015
448. Liborio, A.B., et al. AKI complications in critically ill patients: association with mortality rates and RRT. *Clin J Am Soc Nephrol.* 2015
449. Lichtenstein, K.A., et al. Statin Use Is Associated With Incident Diabetes Mellitus Among Patients in the HIV Outpatient Study. *J Acquir Immune Defic Syndr*. 2015
450. Lim, Y.J., et al. Role of Postoperative Radiotherapy in Nonlocalized Thymoma: Propensity-Matched Analysis of Surveillance, Epidemiology, and End Results Database. *J Thorac Oncol.* 2015
451. Lin, C.C., et al. Androgen deprivation with or without radiation therapy for clinically node-positive prostate cancer. *J Natl Cancer Inst*. 2015
452. Lin, J.J., et al. Survival of patients with stage IV lung cancer with diabetes treated with metformin. *Am J Respir Crit Care Med*. 2015
453. Lopez-Cortes, L.E., et al. Is reduced vancomycin susceptibility a factor associated with poor prognosis in MSSA bacteraemia? *J Antimicrob Chemother*. 2015
454. Lu, N., et al. Total joint arthroplasty and the risk of myocardial infarction: a general population, propensity score-matched cohort study. *Arthritis Rheumatol.* 2015
455. Mack, C.D., et al. Comparative Effectiveness of Oxaliplatin Versus 5-flourouricil in Older Adults: An Instrumental Variable Analysis. *Epidemiology.* 2015
456. MacNeil Vroomen, J., et al. Community-dwelling patients with dementia and their informal caregivers with and without case management: 2-year outcomes of a pragmatic trial. *J Am Med Dir Assoc*. 2015
457. Maehara, A., et al. Comparison of Stent Expansion Guided by Optical Coherence Tomography Versus Intravascular Ultrasound: The ILUMIEN II Study (Observational Study of Optical Coherence Tomography [OCT] in Patients Undergoing Fractional Flow Reserve [FFR] and Percutaneous Coronary Intervention). *JACC Cardiovasc Interv.* 2015
458. Mansur, A., et al. Impact of statin therapy on mortality in patients with sepsis-associated acute respiratory distress syndrome (ARDS) depends on ARDS severity: a prospective observational cohort study BMC Med 2015
459. Mariette, C., et al. Self-expanding covered metallic stent as a bridge to surgery in esophageal cancer: impact on oncologic outcomes. *J Am Coll Surg*. 2015
460. Markar, S., et al. Salvage Surgery After Chemoradiotherapy in the Management of Esophageal Cancer: Is It a Viable Therapeutic Option? *J Clin Oncol.* 2015
461. Marques, H.P., et al. Long-term Results of Domino Liver Transplantation for Hepatocellular Carcinoma Using the Double Piggy-back Technique: A 13-Year Experience. *Ann Surg.* 2015
462. Marubashi, S., et al. Anatomical versus non-anatomical resection for hepatocellular carcinoma. *Br J Surg*. 2015
463. Maura, G., et al. Comparison of the short-term risk of bleeding and arterial thromboembolic events in nonvalvular atrial fibrillation patients newly treated with dabigatran or rivaroxaban versus vitamin K antagonists: a French nationwide propensity-matched cohort study. *Circulation.* 2015
464. May, P., et al. Prospective Cohort Study of Hospital Palliative Care Teams for Inpatients With Advanced Cancer: Earlier Consultation Is Associated With Larger Cost-Saving Effect. *J Clin Oncol.* 2015
465. Meghea, C.I., et al. Statewide Medicaid Enhanced Prenatal Care Programs and Infant Mortality. *Pediatrics*. 2015
466. Messager, M., et al. Laparoscopic Gastric Mobilization Reduces Postoperative Mortality After Esophageal Cancer Surgery: A French Nationwide Study. *Ann Surg.* 2015
467. Mikell, J.L., et al. Postoperative radiotherapy is associated with better survival in non-small cell lung cancer with involved N2 lymph nodes: results of an analysis of the National Cancer Data Base. *J Thorac Oncol.* 2015
468. Miller, M., et al. Prescription opioid duration of action and the risk of unintentional overdose among patients receiving opioid therapy*. JAMA Intern Med.* 2015
469. Min, J.J., et al. Effects of Palonosetron on Perioperative Cardiovascular Complications in Patients Undergoing Noncardiac Surgery With General Anesthesia: A Retrospective Cohort Study. *Clin Pharmacol Ther.* 2015
470. Mogensen, K.M., et al. Nutritional Status and Mortality in the Critically Ill. *Crit Care Med.* 2015
471. Molnar, M.Z., et al. Association of hepatitis C viral infection with incidence and progression of chronic kidney disease in a large cohort of US veterans. *Hepatology.* 2015
472. Molto, A., et al. Brief Report: Nonsteroidal Antiinflammatory Drug-Sparing Effect of Tumor Necrosis Factor Inhibitors in Early Axial Spondyloarthritis: Results From the DESIR Cohort*. Arthritis Rheumatol.* 2015
473. Muniz, L.F., et al. Impact of Therapy on Metabolic Syndrome in Young Adult Premenopausal Female Lupus Patients: Beneficial Effect of Antimalarials. *Arthritis Care Res (Hoboken).* 2015
474. Murakoshi, N., et al. Prognostic impact of supraventricular premature complexes in community-based health checkups: the Ibaraki Prefectural Health Study. *Eur Heart J.* 2015
475. Nadeau-Fredette, A.C., et al. An Incident Cohort Study Comparing Survival on Home Hemodialysis and Peritoneal Dialysis (Australia and New Zealand Dialysis and Transplantation Registry). *Clin J Am Soc Nephrol.* 2015
476. Naganuma, T., et al. Cerebral Microbleeds Predict Intracerebral Hemorrhage in Hemodialysis Patients. *Stroke.* 2015
477. Nakhoul, G.N., et al. Implantable cardioverter-defibrillators in patients with CKD: a propensity-matched mortality analysis. *Clin J Am Soc Nephrol.* 2015
478. Nerland, U.S., et al. Minimally invasive decompression versus open laminectomy for central stenosis of the lumbar spine: pragmatic comparative effectiveness study*. Bmj*. 2015
479. Ng, K.J., et al. Risks of venous thromboembolism in patients with liver cirrhosis: a nationwide cohort study in Taiwan. *J Thromb Haemost*. 2015
480. Nijhoff, F., et al. Transcatheter aortic valve implantation with the new balloon-expandable Sapien 3 versus Sapien XT valve system: a propensity score-matched single-center comparison. *Circ Cardiovasc Interv*. 2015
481. Nussbaum, D.P., et al. Long-term Oncologic Outcomes After Neoadjuvant Radiation Therapy for Retroperitoneal Sarcomas. *Ann Surg*. 2015
482. Nwaogu, I., et al. Venous Thromboembolism after Breast Reconstruction in Patients Undergoing Breast Surgery: An American College of Surgeons NSQIP Analysis. *J Am Coll Surg*. 2015
483. Nyboe Andersen, N., et al. Association between tumour necrosis factor-alpha inhibitors and risk of serious infections in people with inflammatory bowel disease: nationwide Danish cohort study*. Bmj.* 2015
484. Oh, S.H., et al. An observational study of surface versus endovascular cooling techniques in cardiac arrest patients: a propensity-matched analysis. *Crit Care.* 2015
485. Olszewski, A.J., et al. Treatment selection and outcomes in early-stage classical Hodgkin lymphoma: analysis of the National Cancer Data Base. *J Clin Oncol.* 2015
486. Ornstein, K.A., et al. Association Between Hospice Use and Depressive Symptoms in Surviving Spouses. *JAMA Intern Med.* 2015
487. Osho, A.A., et al. Determining eligibility for lung transplantation: A nationwide assessment of the cutoff glomerular filtration rate. *J Heart Lung Transplant.* 2015
488. Osterman, M.T., et al. Effectiveness and Safety of Immunomodulators With Anti-Tumor Necrosis Factor Therapy in Crohn's Disease. *Clin Gastroenterol Hepatol.* 2015
489. Ou, S.M., et al. Effects on Clinical Outcomes of Adding Dipeptidyl Peptidase-4 Inhibitors Versus Sulfonylureas to Metformin Therapy in Patients With Type 2 Diabetes Mellitus. *Ann Intern Med.* 2015
490. Pappas, D.A., et al. Herpes Zoster Reactivation in Patients With Rheumatoid Arthritis: Analysis of Disease Characteristics and Disease-Modifying Antirheumatic Drugs. *Arthritis Care Res (Hoboken).* 2015
491. Park, J.S., et al. Multicentre study of robotic intersphincteric resection for low rectal cancer. *Br J Surg.* 2015
492. Parodi, G., et al. Morphine is associated with a delayed activity of oral antiplatelet agents in patients with ST-elevation acute myocardial infarction undergoing primary percutaneous coronary intervention. *Circ Cardiovasc Interv*. 2015
493. Patel, V.R., et al. Dehydrated Human Amnion/Chorion Membrane Allograft Nerve Wrap Around the Prostatic Neurovascular Bundle Accelerates Early Return to Continence and Potency Following Robot-assisted Radical Prostatectomy: Propensity Score-matched Analysis. *Eur Urol.* 2015
494. Peng, K.P., et al. Increased risk of Bell palsy in patients with migraine: a nationwide cohort study. *Neurology.* 2015
495. Perman, S.M., et al. The Utility of Therapeutic Hypothermia for Post-Cardiac Arrest Syndrome Patients With an Initial Nonshockable Rhythm. *Circulation.* 2015
496. Permar, S.R., et al. Maternal HIV-1 envelope-specific antibody responses and reduced risk of perinatal transmission*. J Clin Invest.* 2015
497. Piessen, G., et al. Laparoscopic Versus Open Surgery for Gastric Gastrointestinal Stromal Tumors: What Is the Impact on Postoperative Outcome and Oncologic Results? *Ann Surg.* 2015
498. Pines, J.M., et al. Emergency department and inpatient hospital use by Medicare beneficiaries in patient-centered medical homes. *Ann Emerg Med.* 2015
499. Pommerening, M.J., et al. Primary skin closure after damage control laparotomy. *Br J Surg*. 2015
500. Potluri, V., et al. Kidney transplant outcomes for prior living organ donors. *J Am Soc Nephrol.* 2015
501. Povoa, P., et al. Clinical impact of stress dose steroids in patients with septic shock: insights from the PROWESS-Shock trial*. Crit Care.* 2015
502. Prieto-Alhambra, D., et al. Hormone replacement therapy and mid-term implant survival following knee or hip arthroplasty for osteoarthritis: a population-based cohort study. *Ann Rheum Dis*. 2015
503. Puig-Asensio, M., et al. Epidemiology and outcome of candidaemia in patients with oncological and haematological malignancies: results from a population-based surveillance in Spain. *Clin Microbiol Infect.* 2015
504. Puri, V., et al. Treatment Outcomes in Stage I Lung Cancer: A Comparison of Surgery and Stereotactic Body Radiation Therapy*. J Thorac Oncol.* 2015
505. Raghunathan, K., et al. Association between Initial Fluid Choice and Subsequent In-hospital Mortality during the Resuscitation of Adults with Septic Shock. *Anesthesiology.* 2015
506. Rajajee, V., et al. Impact of real-time ultrasound guidance on complications of percutaneous dilatational tracheostomy: a propensity score analysis. *Crit Care.* 2015
507. Rathbun, A.M., et al. Temporal effect of depressive symptoms on the longitudinal evolution of rheumatoid arthritis disease activity. *Arthritis Care Res (Hoboken).* 2015
508. Ratib, K., et al. Access site practice and procedural outcomes in relation to clinical presentation in 439,947 patients undergoing percutaneous coronary intervention in the United kingdom. *JACC Cardiovasc Interv.* 2015
509. Rhoads, K.F., et al. How do integrated health care systems address racial and ethnic disparities in colon cancer? *J Clin Oncol*. 2015
510. Riippa, I., et al. A Patient Portal With Electronic Messaging: Controlled Before-and-After Study*. J Med Internet Res.* 2015
511. Rinehart, J., et al. Closed-loop assisted versus manual goal-directed fluid therapy during high-risk abdominal surgery: a case-control study with propensity matching. *Crit Care.* 2015
512. Rokx, C., et al. Increased virological failure in naive HIV-1-infected patients taking lamivudine compared with emtricitabine in combination with tenofovir and efavirenz or nevirapine in the Dutch nationwide ATHENA cohort. *Clin Infect Dis*. 2015
513. Safley, D.M., et al. Impact of Glycoprotein IIb/IIIa Inhibition in Contemporary Percutaneous Coronary Intervention for Acute Coronary Syndromes: Insights From the National Cardiovascular Data Registry. *JACC Cardiovasc Interv.* 2015
514. Sammon, J.D., et al. Patterns of Declining Use and the Adverse Effect of Primary Androgen Deprivation on All-cause Mortality in Elderly Men with Prostate Cancer. *Eur Urol.* 2015
515. Sang, B.H., et al. Hypoalbuminemia Within Two Postoperative Days Is an Independent Risk Factor for Acute Kidney Injury Following Living Donor Liver Transplantation: A Propensity Score Analysis of 998 Consecutive Patients. *Crit Care Med.* 2015
516. Sanghavi, P., et al. Outcomes of Basic Versus Advanced Life Support for Out-of-Hospital Medical Emergencies. *Ann Intern Med.* 2015
517. Sanghavi, P., et al. Outcomes after out-of-hospital cardiac arrest treated by basic vs advanced life support. *JAMA Intern Med.* 2015
518. Santana-Davila, R., et al. Cisplatin and etoposide versus carboplatin and paclitaxel with concurrent radiotherapy for stage III non-small-cell lung cancer: an analysis of Veterans Health Administration data. *J Clin Oncol.* 2015
519. Sawicki, G.S., et al. Sustained Benefit from ivacaftor demonstrated by combining clinical trial and cystic fibrosis patient registry data. *Am J Respir Crit Care Med*. 2015
520. Scarborough, J.E., et al. The impact of functional dependency on outcomes after complex general and vascular surgery. *Ann Surg*. 2015
521. Schermerhorn, M.L., et al. Long-Term Outcomes of Abdominal Aortic Aneurysm in the Medicare Population. *N Engl J Med*. 2015
522. Schwartz, J.L., et al. Contraceptive efficacy, safety, fit, and acceptability of a single-size diaphragm developed with end-user input. *Obstet Gynecol.* 2015
523. Schymik, G., et al. Long-term results of transapical versus transfemoral TAVI in a real world population of 1000 patients with severe symptomatic aortic stenosis. *Circ Cardiovasc Interv*. 2015
524. Seeger, J.D., et al. Safety and effectiveness of dabigatran and warfarin in routine care of patients with atrial fibrillation. *Thromb Haemost.* 2015
525. Seiffge, D.J., et al. Recanalization therapies in acute ischemic stroke patients: impact of prior treatment with novel oral anticoagulants on bleeding complications and outcome. *Circulation.* 2015
526. Sharma, R., et al. Normalization of testosterone level is associated with reduced incidence of myocardial infarction and mortality in men. *Eur Heart J.* 2015
527. Shaw, A.D., et al. Impact of intravenous fluid composition on outcomes in patients with systemic inflammatory response syndrome. *Crit Care.* 2015
528. Sheffrin, M., et al. Weight Loss Associated with Cholinesterase Inhibitors in Individuals with Dementia in a National Healthcare System. *J Am Geriatr Soc.* 2015
529. Shibata, T., et al. Prevalence, Clinical Features, and Prognosis of Acute Myocardial Infarction Attributable to Coronary Artery Embolism. *Circulation.* 2015
530. Shin, J.Y., et al. Risk of intracranial haemorrhage in antidepressant users with concurrent use of non-steroidal anti-inflammatory drugs: nationwide propensity score matched study. *Bmj*. 2015
531. Shin, S.H., et al. A comparative study of laparoscopic vs. open distal pancreatectomy for left-sided ductal adenocarcinoma: a propensity score-matched analysis. *J Am Coll Surg.* 2015
532. Shiomi, A., et al. Effects of a diverting stoma on symptomatic anastomotic leakage after low anterior resection for rectal cancer: a propensity score matching analysis of 1,014 consecutive patients. *J Am Coll Surg.* 2015
533. Sileshi, B., et al. In-hospital outcomes of a minimally invasive off-pump left thoracotomy approach using a centrifugal continuous-flow left ventricular assist device. *J Heart Lung Transplant.* 2015
534. Singh, J.A., et al. Comparative effectiveness of urate lowering with febuxostat versus allopurinol in gout: analyses from large U.S. managed care cohort. *Arthritis Res Ther*. 2015
535. Smith, E.G., et al. Mortality associated with lithium and valproate treatment of US Veterans Health Administration patients with mental disorders*. Br J Psychiatry*. 2015
536. Snider, J.T., et al. Effect of hospital use of oral nutritional supplementation on length of stay, hospital cost, and 30-day readmissions among Medicare patients with COPD. *Chest.* 2015
537. Song, K.D., et al. Repeated Hepatic Resection versus Radiofrequency Ablation for Recurrent Hepatocellular Carcinoma after Hepatic Resection: A Propensity Score Matching Study. *Radiology.* 2015
538. Soubrane, O., et al. Laparoscopic Living Donor Left Lateral Sectionectomy: A New Standard Practice for Donor Hepatectomy. *Ann Surg.* 2015
539. Stefan, M.S., et al. Comparative Effectiveness of Noninvasive and Invasive Ventilation in Critically Ill Patients With Acute Exacerbation of Chronic Obstructive Pulmonary Disease. *Crit Care Med*. 2015
540. Stey, A., et al. Outcomes and costs of surgical treatments of necrotizing enterocolitis. *Pediatrics.* 2015
541. Suzuki, S., et al. Paracetamol therapy and outcome of critically ill patients: a multicenter retrospective observational study. *Crit Care.* 2015
542. Tagami, T., et al. Supplemental dose of antithrombin use in disseminated intravascular coagulation patients after abdominal sepsis. *Thromb Haemost.* 2015
543. Tagami, T., et al. Intravenous immunoglobulin and mortality in pneumonia patients with septic shock: an observational nationwide study. *Clin Infect Dis*. 2015
544. Tagami, T., et al. Recombinant human soluble thrombomodulin and mortality in severe pneumonia patients with sepsis-associated disseminated intravascular coagulation: an observational nationwide study*. J Thromb Haemost.* 2015
545. Tagami, T., et al. Low-dose corticosteroid use and mortality in severe community-acquired pneumonia patients. *Eur Respir J*. 2015
546. Takahashi, J., et al. Prognostic impact of chronic nitrate therapy in patients with vasospastic angina: multicentre registry study of the Japanese coronary spasm association. *Eur Heart J.* 2015
547. Tamburino, C., et al. 1-Year Outcomes After Transfemoral Transcatheter or Surgical Aortic Valve Replacement: Results From the Italian OBSERVANT Study. *J Am Coll Cardiol.* 2015
548. Tamiya, H., et al. Comparison of short-term mortality and morbidity between parenteral and enteral nutrition for adults without cancer: a propensity-matched analysis using a national inpatient database. *Am J Clin Nutr*. 2015
549. Tamma, P.D., et al. Carbapenem therapy is associated with improved survival compared with piperacillin-tazobactam for patients with extended-spectrum beta-lactamase bacteremia. *Clin Infect Dis.* 2015
550. Tanaka, S., et al. Secondhand smoke and incidence of dental caries in deciduous teeth among children in Japan: population based retrospective cohort study. *Bmj.* 2015
551. Tandon, P., et al. Risk of Bacterial Infection in Patients With Cirrhosis and Acute Variceal Hemorrhage, Based on Child-Pugh Class, and Effects of Antibiotics. *Clin Gastroenterol Hepatol.* 2015
552. Tandon, S.D., et al. Depression outcomes associated with an intervention implemented in employment training programs for low-income adolescents and young adults. *JAMA Psychiatry.* 2015
553. Taniguchi, T., et al. Initial Surgical Versus Conservative Strategies in Patients With Asymptomatic Severe Aortic Stenosis. *J Am Coll Cardiol.* 2015
554. Tanriover, B., et al. Induction Therapies in Live Donor Kidney Transplantation on Tacrolimus and Mycophenolate With or Without Steroid Maintenance. *Clin J Am Soc Nephrol.* 2015
555. Tarantino, I., et al. Prognostic Relevance of Palliative Primary Tumor Removal in 37,793 Metastatic Colorectal Cancer Patients: A Population-Based, Propensity Score-Adjusted Trend Analysis*. Ann Surg*. 2015
556. Thukkani, A.K., et al. Long-Term Outcomes in Patients With Diabetes Mellitus Related to Prolonging Clopidogrel More Than 12 Months After Coronary Stenting. *J Am Coll Cardiol.* 2015
557. Tomasello, S.D., et al. Management strategies in patients affected by chronic total occlusions: results from the Italian Registry of Chronic Total Occlusions. *Eur Heart J.* 2015
558. Tsai, C.L., et al. Gastro-oesophageal reflux disease increases the risk of intensive care unit admittance and mechanical ventilation use among patients with chronic obstructive pulmonary disease: a nationwide population-based cohort study. *Crit Care*. 2015
559. Tschan, F., et al. Impact of case-relevant and case-irrelevant communication within the surgical team on surgical-site infection. *Br J Surg.* 2015
560. Turner, D., et al. Efficacy of oral methotrexate in paediatric Crohn's disease: a multicentre propensity score study. *Gut.* 2015
561. Van der Linden, P., et al. Efficacy and safety of 6% hydroxyethyl starch 130/0.4 (Voluven) for perioperative volume replacement in children undergoing cardiac surgery: a propensity-matched analysis. *Crit Care*. 2015
562. Vargo, J.A., et al. Treatment Selection and Survival Outcomes in Early-Stage Diffuse Large B-Cell Lymphoma: Do We Still Need Consolidative Radiotherapy? *J Clin Oncol.* 2015
563. Veluswamy, R.R., et al. Limited Resection Versus Lobectomy for Older Patients With Early-Stage Lung Cancer: Impact of Histology*. J Clin Oncol.* 2015
564. Vidal-Ribas, P., et al. Positive attributes in children and reduced risk of future psychopathology. *Br J Psychiatry.* 2015
565. Vigod, S.N., et al. Antipsychotic drug use in pregnancy: high dimensional, propensity matched, population based cohort study. *Bmj.* 2015
566. Villines, T.C., et al. A comparison of the safety and effectiveness of dabigatran and warfarin in non-valvular atrial fibrillation patients in a large healthcare system. *Thromb Haemost*. 2015
567. Vyas, A., et al. Early Coronary Angiography and Survival After Out-of-Hospital Cardiac Arrest. *Circ Cardiovasc Interv*. 2015
568. Wang, B.Y., et al. Single-incision versus multiple-incision thoracoscopic lobectomy and segmentectomy: a propensity-matched analysis. *Ann Surg*. 2015
569. Wang, E.H., et al. Patients Selected for Definitive Concurrent Chemoradiation at High-volume Facilities Achieve Improved Survival in Stage III Non-Small-Cell Lung Cancer. *J Thorac Oncol.* 2015
570. Wang, I.K., et al. Comparison of Subdural Hematoma Risk between Hemodialysis and Peritoneal Dialysis Patients with ESRD. *Clin J Am Soc Nephrol*. 2015
571. Wang, X., et al. Mannitol and Outcome in Intracerebral Hemorrhage: Propensity Score and Multivariable Intensive Blood Pressure Reduction in Acute Cerebral Hemorrhage Trial 2 Results. *Stroke*. 2015
572. Weir, M.A., et al. beta-Blocker dialyzability and mortality in older patients receiving hemodialysis*. J Am Soc Nephrol.* 2015
573. Weisberg, D.F., et al. Long-term Prescription of Opioids and/or Benzodiazepines and Mortality Among HIV-Infected and Uninfected Patients. *J Acquir Immune Defic Syndr.* 2015
574. Welk, B., et al. The risk of fall and fracture with the initiation of a prostate-selective alpha antagonist: a population based cohort study. *Bmj*. 2015
575. Whitlock, E.L., et al. Perioperative Mortality, 2010 to 2014: A Retrospective Cohort Study Using the National Anesthesia Clinical Outcomes Registry. *Anesthesiology.* 2015
576. Willingham, M.D., et al. Concurrence of Intraoperative Hypotension, Low Minimum Alveolar Concentration, and Low Bispectral Index Is Associated with Postoperative Death. *Anesthesiology.* 2015
577. Wilson, M.Z., et al. Ulcerative Colitis Is Associated With an Increased Risk of Venous Thromboembolism in the Postoperative Period: The Results of a Matched Cohort Analysis. *Ann Surg.* 2015
578. Winn, A.N., et al. The real world effectiveness of hematopoietic transplant among elderly individuals with multiple myeloma. *J Natl Cancer Inst.* 2015
579. Wong, G.L., et al. Long-term safety of oral nucleos(t)ide analogs for patients with chronic hepatitis B: A cohort study of 53,500 subjects. *Hepatology.* 2015
580. Wright, A.A., et al. Use and Effectiveness of Intraperitoneal Chemotherapy for Treatment of Ovarian Cancer. *J Clin Oncol.* 2015
581. Wu, P.C., et al. Pentoxifylline Decreases Dialysis Risk in Patients With Advanced Chronic Kidney Disease*. Clin Pharmacol Ther*. 2015
582. Xing, J., et al. Care coordination program for Washington State Medicaid enrollees reduced inpatient hospital costs. *Health Aff (Millwood).* 2015
583. Xu, R., et al. Hyponatremia and Cognitive Impairment in Patients Treated with Peritoneal Dialysis. *Clin J Am Soc Nephrol.* 2015
584. Yang, Y., et al. Risk-adapted therapy for early-stage extranodal nasal-type NK/T-cell lymphoma: analysis from a multicenter study. *Blood.* 2015
585. Yehya, N., et al. Corticosteroid exposure in pediatric acute respiratory distress syndrome. *Intensive Care Med.* 2015
586. Yoshihara, M., et al. The efficacy of recombinant human soluble thrombomodulin for obstetric disseminated intravascular coagulation: a retrospective study. *Crit Care.* 2015
587. Yoshimura, J., et al. Benefit profile of recombinant human soluble thrombomodulin in sepsis-induced disseminated intravascular coagulation: a multicenter propensity score analysis. *Crit Care.* 2015
588. Yu, C.W., et al. Long-Term Clinical Outcomes of Final Kissing Ballooning in Coronary Bifurcation Lesions Treated With the 1-Stent Technique: Results From the COBIS II Registry (Korean Coronary Bifurcation Stenting Registry). *JACC Cardiovasc Interv*. 2015
589. Yuan, C., et al. Survival among patients with pancreatic cancer and long-standing or recent-onset diabetes mellitus. *J Clin Oncol*. 2015
590. Zhang, Z., et al. Cost-effectiveness of revascularization strategies: the ASCERT study*. J Am Coll Cardiol.* 2015
591. Zheng, Z., et al. Comparative effectiveness of laparoscopy vs open colectomy among nonmetastatic colon cancer patients: an analysis using the National Cancer Data Base. *J Natl Cancer Inst*. 2015
592. Zywiel, M.G., et al. Health economic implications of perioperative delirium in older patients after surgery for a fragility hip fracture. *J Bone Joint Surg Am.* 2015
593. Horkan, C.M., et al. The association of acute kidney injury in the critically ill and postdischarge outcomes: a cohort study*. *Crit Care Med*. 2015
594. Lin, T.T., et al. Primary prevention of atrial fibrillation with angiotensin-converting enzyme inhibitors and angiotensin receptor blockers in patients with end-stage renal disease undergoing dialysis. *Kidney Int.* 2015
595. Tesar, V., et al. Corticosteroids in IgA Nephropathy: A Retrospective Analysis from the VALIGA Study*. J Am Soc Nephrol*. 2015
596. Adams, K.F., Jr., et al. Dose response characterization of the association of serum digoxin concentration with mortality outcomes in the Digitalis Investigation Group trial. *Eur J Heart Fail.* 2016
597. Alexoff, A., et al. Inpatient Costs for Patients with Inflammatory Bowel Disease and Acute Pancreatitis*. Inflamm Bowel Dis*. 2016
598. Al-Holou, S.N., et al. The Association of Statin Use with Cataract Progression and Cataract Surgery: The AREDS2 Report Number 8. *Ophthalmology*. 2016
599. Alite, F., et al. Local control dependence on consecutive vs. nonconsecutive fractionation in lung stereotactic body radiation therapy. *Radiother Oncol.* 2016
600. Al-Sukhni, E., et al. No Survival Difference with Neoadjuvant Chemoradiotherapy Compared with Chemotherapy in Resectable Esophageal and Gastroesophageal Junction Adenocarcinoma: Results from the National Cancer Data Base. *J Am Coll Surg.* 2016
601. Altorki, N.K., et al. Anatomical Segmentectomy and Wedge Resections Are Associated with Comparable Outcomes for Patients with Small cT1N0 Non-Small Cell Lung Cancer. *J Thorac Oncol.* 2016
602. Alvarez-Garcia, J., et al. Electrophysiological Effects of Selective Atrial Coronary Artery Occlusion in Humans. *Circulation.* 2016
603. Amin, S., et al. Metformin Improves Survival in Patients with Pancreatic Ductal Adenocarcinoma and Pre-Existing Diabetes: A Propensity Score Analysis. *Am J Gastroenterol.* 2016
604. Ammann, E.M., et al. Intravenous immune globulin and thromboembolic adverse events in patients with hematologic malignancy. *Blood.* 2016
605. Ananthakrishnan, A.N., et al. Statin Use Is Associated With Reduced Risk of Colorectal Cancer in Patients With Inflammatory Bowel Diseases*. Clin Gastroenterol Hepatol.* 2016
606. Andersen, L.W., et al. Early administration of epinephrine (adrenaline) in patients with cardiac arrest with initial shockable rhythm in hospital: propensity score matched analysis. *Bmj.* 2016
607. Aquina, C.T., et al. Patients With Adhesive Small Bowel Obstruction Should Be Primarily Managed by a Surgical Team. *Ann Surg.* 2016
608. Aquina, C.T., et al. Missed Opportunity: Laparoscopic Colorectal Resection Is Associated With Lower Incidence of Small Bowel Obstruction Compared to an Open Approach. *Ann Surg.* 2016
609. Aranson, N.J., et al. Chronic Kidney Disease Class Predicts Mortality After Abdominal Aortic Aneurysm Repair in Propensity-matched Cohorts From the Medicare Population. *Ann Surg.* 2016
610. Asada, T., et al. Organ System Network Disruption in Nonsurvivors of Critically Ill Patients Crit Care Med 2016
611. Aso, S., et al. The Effect of Intraaortic Balloon Pumping Under Venoarterial Extracorporeal Membrane Oxygenation on Mortality of Cardiogenic Patients: An Analysis Using a Nationwide Inpatient Database. *Crit Care Med.* 2016
612. Atzema, C.L., et al. Outcomes among patients discharged from the emergency department with a diagnosis of peripheral vertigo. *Ann Neurol.* 2016
613. Avgil-Tsadok, M., et al. Dabigatran use in elderly patients with atrial fibrillation Thromb Haemost 2016
614. Ayling, O.G., et al. Dissociation of Early and Delayed Cerebral Infarction After Aneurysmal Subarachnoid Hemorrhage. *Stroke.* 2016
615. Azzalini, L., et al. Procedural and Long-Term Outcomes of Bioresorbable Scaffolds Versus Drug-Eluting Stents in Chronic Total Occlusions: The BONITO Registry (Bioresorbable Scaffolds Versus Drug-Eluting Stents in Chronic Total Occlusions). *Circ Cardiovasc Interv*. 2016
616. Banerjee, M., et al. Use of imaging tests after primary treatment of thyroid cancer in the United States: population based retrospective cohort study evaluating death and recurrence. *Bmj*. 2016
617. Bangalore, S., et al. Revascularization in Patients With Multivessel Coronary Artery Disease and Severe Left Ventricular Systolic Dysfunction: Everolimus-Eluting Stents Versus Coronary Artery Bypass Graft Surgery. *Circulation.* 2016
618. Bateman, B.T., et al. Late Pregnancy beta Blocker Exposure and Risks of Neonatal Hypoglycemia and Bradycardia. *Pediatrics.* 2016
619. Bateman, S.T., et al. Early High-Frequency Oscillatory Ventilation in Pediatric Acute Respiratory Failure. A Propensity Score Analysis. *Am J Respir Crit Care Med.* 2016
620. Bekkar, S., et al. Multicentre study of neoadjuvant chemotherapy for stage I and II oesophageal cancer. *Br J Surg*. 2016
621. Bentzer, P., et al. Plasma cytokine levels predict response to corticosteroids in septic shock. *Intensive Care Med*. 2016
622. Beppu, T., et al. Portal Vein Embolization Followed by Right-Side Hemihepatectomy for Hepatocellular Carcinoma Patients: A Japanese Multi-Institutional Study. *J Am Coll Surg.* 2016
623. Bertelsen, C.A., et al. Short-term outcomes after complete mesocolic excision compared with 'conventional' colonic cancer surgery. *Br J Surg*. 2016
624. Berthelot-Richer, M., et al. Discordant Grading of Aortic Stenosis Severity: Echocardiographic Predictors of Survival Benefit Associated With Aortic Valve Replacement. *JACC Cardiovasc Imaging.* 2016
625. Berwanger, O., et al. Association between pre-operative statin use and major cardiovascular complications among patients undergoing non-cardiac surgery: the VISION study. *Eur Heart J.* 2016
626. Bhatt, S.P., et al. beta-Blockers are associated with a reduction in COPD exacerbations. *Thorax.* 2016
627. Billot, L., et al. Impact Evaluation of a System-Wide Chronic Disease Management Program on Health Service Utilisation: A Propensity-Matched Cohort Study. *PLoS Med.* 2016
628. Bito, S., et al. Mechanical prophylaxis is a heparin-independent risk for anti-platelet factor 4/heparin antibody formation after orthopedic surgery. *Blood.* 2016
629. Blanke, P., et al. Long-Term Prognostic Utility of Coronary CT Angiography in Stable Patients With Diabetes Mellitus. *JACC Cardiovasc Imaging*. 2016
630. Blecker, S., et al. Observation Units as Substitutes for Hospitalization or Home Discharge. *Ann Emerg Med.* 2016
631. Blitz, J.D., et al. Preoperative Evaluation Clinic Visit Is Associated with Decreased Risk of In-hospital Postoperative Mortality. *Anesthesiology*. 2016
632. Bomberg, H., et al. Single-dose Antibiotic Prophylaxis in Regional Anesthesia: A Retrospective Registry Analysis. *Anesthesiology.* 2016
633. Brauer, D.G., et al. Operative Site Drainage after Hepatectomy: A Propensity Score Matched Analysis Using the American College of Surgeons NSQIP Targeted Hepatectomy Database. *J Am Coll Surg*. 2016
634. Brian Cassel, J., et al. Effect of a Home-Based Palliative Care Program on Healthcare Use and Costs. *J Am Geriatr Soc.* 2016
635. Broeg-Morvay, A., et al. Direct Mechanical Intervention Versus Combined Intravenous and Mechanical Intervention in Large Artery Anterior Circulation Stroke: A Matched-Pairs Analysis. *Stroke*. 2016
636. Brookhart, M.A., et al. Comparative Short-term Safety of Sodium Ferric Gluconate Versus Iron Sucrose in Hemodialysis Patients. *Am J Kidney Dis.* 2016
637. Brouwer, T.F., et al. Long-Term Clinical Outcomes of Subcutaneous Versus Transvenous Implantable Defibrillator Therapy. *J Am Coll Cardiol.* 2016
638. Brown, J.B., et al. Geographic Variation in Outcome Benefits of Helicopter Transport for Trauma in the United States: A Retrospective Cohort Study. *Ann Surg*. 2016
639. Brunet, L., et al. Progression of Liver Fibrosis and Modern Combination Antiretroviral Therapy Regimens in HIV-Hepatitis C-Coinfected Persons. *Clin Infect Dis.* 2016
640. Bulka, C.M., et al. Nondepolarizing Neuromuscular Blocking Agents, Reversal, and Risk of Postoperative Pneumonia. *Anesthesiology.* 2016
641. Byrne, J.P., et al. Timing of Pharmacologic Venous Thromboembolism Prophylaxis in Severe Traumatic Brain Injury: A Propensity-Matched Cohort Study. *J Am Coll Surg*. 2016
642. Cadilhac, D.A., et al. Better outcomes for hospitalized patients with TIA when in stroke units: An observational study. *Neurology.* 2016
643. Cao, B., et al. Adjuvant Corticosteroid Treatment in Adults With Influenza A (H7N9) Viral Pneumonia. *Crit Care Med*. 2016
644. Capodanno, D., et al. Computing Methods for Composite Clinical Endpoints in Unprotected Left Main Coronary Artery Revascularization: A Post Hoc Analysis of the DELTA Registry. *JACC Cardiovasc Interv*. 2016
645. Cappabianca, G., et al. Safety and efficacy of prothrombin complex concentrate as first-line treatment in bleeding after cardiac surgery. *Crit Care.* 2016
646. Castleberry, C.D., et al. Early initiation of mTOR inhibitors in children with heart transplantation: A propensity-based registry analysis. *J Heart Lung Transplant.* 2016
647. Cauchy, F., et al. Benefits of Laparoscopy in Elderly Patients Requiring Major Liver Resection. *J Am Coll Surg.* 2016
648. Chan, R.C., et al. Clinical Utility and Lifespan Profiling of Neurological Soft Signs in Schizophrenia. *Spectrum Disorders Schizophr Bull.* 2016
649. Chan, Y.H., et al. Thromboembolic, Bleeding, and Mortality Risks of Rivaroxaban and Dabigatran in Asians With Nonvalvular Atrial Fibrillation. *J Am Coll Cardiol.* 2016
650. Chan, Y.H., et al. Acute Kidney Injury in Asians With Atrial Fibrillation Treated With Dabigatran or Warfarin*. J Am Coll Cardiol.* 2016
651. Chao, P.W., et al. Long-Term Outcomes in Critically Ill Septic Patients Who Survived Cardiopulmonary Resuscitation*. Crit Care Med*. 2016
652. Chen, C.Y., et al. Does higher radiation dose lead to better outcome for non-operated localized esophageal squamous cell carcinoma patients who received concurrent chemoradiotherapy? A population based propensity-score matched analysis. *Radiother Oncol.* 2016
653. Chen, Y.C., et al. Clinical outcomes after spontaneous and nucleos(t)ide analogue-treated HBsAg seroclearance in chronic HBV infection Aliment. *Pharmacol Ther.* 2016
654. Cheng, F.W., et al. Body mass index and all-cause mortality among older adults. *Obesity (Silver Spring).* 2016
655. Chesnaye, N.C., et al. Mortality risk in European children with end-stage renal disease on dialysis. *Kidney Int.* 2016
656. Chester Wasko, M., et al. Prednisone Use and Risk of Mortality in Patients With Rheumatoid Arthritis: Moderation by Use of Disease-Modifying Antirheumatic Drugs. *Arthritis Care Res (Hoboken).* 2016
657. Cheung, A.C., et al. Combined ursodeoxycholic acid (UDCA) and fenofibrate in primary biliary cholangitis patients with incomplete UDCA response may improve outcomes. *Aliment Pharmacol Ther.* 2016
658. Cheung, T.T., et al. Pure Laparoscopic Hepatectomy Versus Open Hepatectomy for Hepatocellular Carcinoma in 110 Patients With Liver Cirrhosis: A Propensity Analysis at a Single Center. *Ann Surg*. 2016
659. Chiang, A., et al. A comparison between accelerated hypofractionation and stereotactic ablative radiotherapy (SABR) for early-stage non-small cell lung cancer (NSCLC): Results of a propensity score-matched analysis. *Radiother Oncol.* 2016
660. Chiotos, K., et al. Comparative effectiveness of echinocandins versus fluconazole therapy for the treatment of adult candidaemia due to Candida parapsilosis: a retrospective observational cohort study of the Mycoses Study Group (MSG-12). *J Antimicrob Chemother*. 2016
661. Chiu, M., et al. Moving to a Highly Walkable Neighborhood and Incidence of Hypertension: A Propensity-Score Matched Cohort Study. *Environ Health Perspect*. 2016
662. Cipriani, F., et al. Propensity score-based analysis of outcomes of laparoscopic versus open liver resection for colorectal metastases. *Br J Surg.* 2016
663. Clark, E., et al. Septic shock in chronic dialysis patients: clinical characteristics, antimicrobial therapy and mortality. *Intensive Care Med.* 2016
664. Claushuis, T.A., et al. Thrombocytopenia is associated with a dysregulated host response in critically ill sepsis patients. *Blood.* 2016
665. Couture, E.L., et al. Culprit Vessel Revascularization Prior to Diagnostic Angiography as a Strategy to Reduce Delays in Primary Percutaneous Coronary Intervention: A Propensity-Matched Analysis. *Circ Cardiovasc Interv.* 2016
666. Dad, T., et al. Aspirin Use and Incident Cardiovascular Disease, Kidney Failure, and Death in Stable Kidney Transplant Recipients: A Post Hoc Analysis of the Folic Acid for Vascular Outcome Reduction in Transplantation (FAVORIT) Trial. *Am J Kidney Dis.* 2016
667. Damman, K., et al. Loop diuretics, renal function and clinical outcome in patients with heart failure and reduced ejection fraction. *Eur J Heart Fail.* 2016
668. Daniel, P., et al. Time to first antibiotic and mortality in adults hospitalised with community-acquired pneumonia: a matched-propensity analysis. *Thorax.* 2016
669. Datta, J., et al. Implications of Lymph Node Staging on Selection of Adjuvant Therapy for Gastric Cancer in the United States: A Propensity Score-matched Analysis*. Ann Surg.* 2016
670. de Waard, G.A., et al. Changes in Coronary Blood Flow After Acute Myocardial Infarction: Insights From a Patient Study and an Experimental Porcine Model. *JACC Cardiovasc Interv.* 2016
671. Delaney, J.W., et al. The influence of corticosteroid treatment on the outcome of influenza A(H1N1pdm09)-related critical illness. *Crit Care.* 2016
672. Diller, G.P., et al. Depression requiring anti-depressant drug therapy in adult congenital heart disease: prevalence, risk factors, and prognostic value. *Eur Heart J.* 2016
673. Donneyong, M.M., et al. Risk of mortality with concomitant use of tamoxifen and selective serotonin reuptake inhibitors: multi-database cohort study. *Bmj*. 2016
674. Ecker, B.L., et al. Preoperative radiotherapy in the management of retroperitoneal liposarcoma Br J Surg 2016
675. Edeline, J., et al. Selective internal radiation therapy compared with sorafenib for hepatocellular carcinoma with portal vein thrombosis. *Eur J Nucl Med Mol Imaging*. 2016
676. Etminan, M., et al. Risk of Myocardial Infarction and Stroke With Single or Repeated Doses of Intravitreal Bevacizumab in Age-Related Macular Degeneration. *Am J Ophthalmol.* 2016
677. Faraoni, D., et al. Post-Operative Outcomes in Children With and Without Congenital Heart Disease Undergoing Noncardiac Surgery. *J Am Coll Cardiol.* 2016
678. Favalli, E.G., et al. Twelve-Year Retention Rate of First-Line Tumor Necrosis Factor Inhibitors in Rheumatoid Arthritis: Real-Life Data From a Local Registry. *Arthritis Care Res (Hoboken).* 2016
679. Ferraris, V.A., et al. Impact of Residents on Surgical Outcomes in High-Complexity Procedures. *J Am Coll Surg.* 2016
680. Fu, A.Z., et al. Association Between Hospitalization for Heart Failure and Dipeptidyl Peptidase 4 Inhibitors in Patients With Type 2 Diabetes: An Observational Study. *Diabetes Care.* 2016
681. Fuks, D., et al. Laparoscopy Decreases Pulmonary Complications in Patients Undergoing Major Liver Resection: A Propensity Score Analysis. *Ann Surg*. 2016
682. Fukuda, T., et al. Conventional Versus Compression-Only Versus No-Bystander Cardiopulmonary Resuscitation for Pediatric Out-of-Hospital Cardiac Arrest. *Circulation.* 2016
683. Furrer, R., et al. Postpartum Blood Loss in Women Treated for Intrahepatic Cholestasis of Pregnancy*. Obstet Gynecol.* 2016
684. Gagne, J.J., et al. Successful Comparison of US Food and Drug Administration Sentinel Analysis Tools to Traditional Approaches in Quantifying a Known Drug-Adverse Event Association. *Clin Pharmacol Ther.* 2016
685. Galsky, M.D., et al. Effectiveness of Adjuvant Chemotherapy for Locally Advanced Bladder Cancer. *J Clin Oncol.* 2016
686. Garcia-Huidobro, D., et al. Effect of Patient-Centered Medical Home on Preventive Services for Adolescents and Young Adults. *Pediatrics.* 2016
687. Gershengorn, H.B., et al. Association Between Overnight Extubations and Outcomes in the Intensive Care Unit. *JAMA Intern Med.* 2016
688. Goldhar, H.A., et al. The Temporal Risk of Heart Failure Associated With Adjuvant Trastuzumab in Breast Cancer Patients: A Population Study. *J Natl Cancer Inst*. 2016
689. Goossens, N., et al. Nonalcoholic Steatohepatitis Is Associated With Increased Mortality in Obese Patients Undergoing Bariatric Surgery. *Clin Gastroenterol Hepatol.* 2016
690. Gupta, P., et al. Association of Freestanding Children's Hospitals With Outcomes in Children With Critical Illness. *Crit Care Med.* 2016
691. Gupta, P., et al. Effect of Inhaled Nitric Oxide on Outcomes in Children With Acute Lung Injury: Propensity Matched Analysis From a Linked Database. *Crit Care Med.* 2016
692. Gutierrez-Gutierrez, B., et al. Ertapenem for the treatment of bloodstream infections due to ESBL-producing Enterobacteriaceae: a multinational pre-registered cohort study. *J Antimicrob Chemother*. 2016
693. Hafezi-Nejad, N., et al. Long term use of analgesics and risk of osteoarthritis progressions and knee replacement: propensity score matched cohort analysis of data from the Osteoarthritis Initiative. *Osteoarthritis Cartilage*. 2016
694. Hakeem, A., et al. Long-Term Prognosis of Deferred Acute Coronary Syndrome Lesions Based on Nonischemic Fractional Flow Reserve. *J Am Coll Cardiol*. 2016
695. Hambraeus, K., et al. Long-Term Outcome of Incomplete Revascularization After Percutaneous Coronary Intervention in SCAAR (Swedish Coronary Angiography and Angioplasty Registry). *JACC Cardiovasc Interv.* 2016
696. Han, S., et al. Safety of the Use of Blood Salvage and Autotransfusion During Liver Transplantation for Hepatocellular Carcinoma. *Ann Surg*. 2016
697. Hannan, E.L., et al. Utilization and 1-Year Mortality for Transcatheter Aortic Valve Replacement and Surgical Aortic Valve Replacement in New York Patients With Aortic Stenosis: 2011 to 2012. *JACC Cardiovasc Interv.* 2016
698. Haque, W., et al. Changes in treatment patterns and impact of radiotherapy for early stage diffuse large B cell lymphoma after Rituximab: A population-based analysis. *Radiother Oncol*. 2016
699. Haroon, M., et al. Certain class I HLA alleles and haplotypes implicated in susceptibility play a role in determining specific features of the psoriatic arthritis phenotype. *Ann Rheum Dis*. 2016
700. Harrold, L.R., et al. Comparative effectiveness of abatacept versus tocilizumab in rheumatoid arthritis patients with prior TNFi exposure in the US Corrona registry. *Arthritis Res Ther*. 2016
701. Havers, F., et al. Statin Use and Hospital Length of Stay Among Adults Hospitalized With Community-acquired Pneumonia. *Clin Infect Dis.* 2016
702. Hayes, J.F., et al. Adverse Renal, Endocrine, Hepatic, and Metabolic Events during Maintenance Mood Stabilizer Treatment for Bipolar Disorder: A Population-Based Cohort Study. *PLoS Med.* 2016
703. Hayes, J.F., et al. Self-harm, Unintentional Injury, and Suicide in Bipolar Disorder During Maintenance Mood Stabilizer Treatment: A UK Population-Based Electronic Health Records Study. *JAMA Psychiatry*. 2016
704. He, L., et al. Bayesian regression analyses of radiation modality effects on pericardial and pleural effusion and survival in esophageal cancer. *Radiother Oncol.* 2016
705. Hero, C., et al. Association Between Use of Lipid-Lowering Therapy and Cardiovascular Diseases and Death in Individuals With Type 1 Diabetes. *Diabetes Care.* 2016
706. Hogan, M.E., et al. Incremental health care costs for chronic pain in Ontario, Canada: a population-based matched cohort study of adolescents and adults using administrative data. *Pain.* 2016
707. Holihan, J.L., et al. Is Nonoperative Management Warranted in Ventral Hernia Patients With Comorbidities?: A Case-matched, Prospective, Patient-centered Study. *Ann Surg.* 2016
708. Holubar, S.D., et al. Neoadjuvant Radiotherapy: A Risk Factor for Short-Term Wound Complications after Radical Resection for Rectal Cancer? *J Am Coll Surg*. 2016
709. Honda, M., et al. Long-term Outcomes of Laparoscopic Versus Open Surgery for Clinical Stage I Gastric Cancer: The LOC-1 Study. *Ann Surg*. 2016
710. Honjo, K., et al. Marital Transition and Risk of Stroke: How Living Arrangement and Employment Status Modify Associations. *Stroke.* 2016
711. Horneff, G., et al. Comparison of treatment response, remission rate and drug adherence in polyarticular juvenile idiopathic arthritis patients treated with etanercept, adalimumab or tocilizumab. *Arthritis Res Ther.* 2016
712. Hou, W.H., et al. Dipeptidyl peptidase-4 inhibitor use is not associated with elevated risk of severe joint pain in patients with type 2 diabetes: a population-based cohort study. *Pain*. 2016
713. Hsieh, R.W., et al. The Association Between Internet Use and Ambulatory Care-Seeking Behaviors in Taiwan: A Cross-Sectional Study. *J Med Internet Res.* 2016
714. Hsu, C.W., et al. Risk of type 2 diabetes mellitus in patients with acute critical illness: a population-based cohort study. *Intensive Care Med.* 2016
715. Huang, K.P., et al. Cardiovascular risk in patients with alopecia areata (AA): A propensity-matched retrospective analysis*. J Am Acad Dermatol.* 2016
716. Huang, Y.W., et al. Statins Reduce the Risk of Cirrhosis and Its Decompensation in Chronic Hepatitis B Patients: A Nationwide Cohort Study. *Am J Gastroenterol.* 2016
717. Huybrechts, K.F., et al. Antipsychotic Use in Pregnancy and the Risk for Congenital Malformations. *JAMA Psychiatry.* 2016
718. Hwang, J.Y., et al. A Propensity-matched Analysis Comparing Survival After Esophagectomy Followed by Adjuvant Chemoradiation to Surgery Alone for Esophageal Squamous Cell Carcinoma. *Ann Surg*. 2016
719. Inghammar, M., et al. Oral fluoroquinolone use and serious arrhythmia: bi-national cohort study. *Bmj.* 2016
720. Iqbal, J., et al. Outcomes Following Primary Percutaneous Coronary Intervention in Patients With Previous Coronary Artery Bypass Surgery. *Circ Cardiovasc Interv.* 2016
721. Jackson, W.C., et al. Duration of Androgen Deprivation Therapy Influences Outcomes for Patients Receiving Radiation Therapy Following Radical Prostatectomy. *Eur Urol.* 2016
722. Johansson, I., et al. Prognostic Implications of Type 2 Diabetes Mellitus in Ischemic and Nonischemic Heart Failure*. J Am Coll Cardiol.* 2016
723. Kahn, J.A., et al. Substantial Decline in Vaccine-Type Human Papillomavirus (HPV) Among Vaccinated Young Women During the First 8 Years After HPV Vaccine Introduction in a Community. *Clin Infect Dis.* 2016
724. Kan, J., et al. Incidence and Clinical Outcomes of Stent Fractures on the Basis of 6,555 Patients and 16,482 Drug-Eluting Stents From 4 Centers. *JACC Cardiovasc Interv.* 2016
725. Kang, T.W., et al. Long-term Therapeutic Outcomes of Radiofrequency Ablation for Subcapsular versus Nonsubcapsular Hepatocellular Carcinoma: A Propensity Score Matched Study*. Radiology.* 2016
726. Kanwal, F., et al. Early outpatient follow-up and 30-day outcomes in patients hospitalized with cirrhosis. *Hepatology*. 2016
727. Kim, G.A., et al. Radiofrequency ablation as an alternative to hepatic resection for single small hepatocellular carcinomas. *Br J Surg.* 2016
728. Kim, H.S., et al. Association Between Level of Fibrosis, Rather Than Antiviral Regimen, and Outcomes of Patients With Chronic Hepatitis B.  *Clin Gastroenterol Hepatol.* 2016
729. Kim, J.H., et al. Association between obesity and lower urinary tract symptoms: propensity score matching study between healthy controls and obese patients seeking bariatric surgery. *Surg Obes Relat Dis.* 2016
730. Kim, M.J., et al. Preventive Suboccipital Decompressive Craniectomy for Cerebellar Infarction: A Retrospective-Matched Case-Control Study. *Stroke.* 2016
731. Kim, S.C., et al. Biologic Disease-Modifying Antirheumatic Drugs and Risk of High-Grade Cervical Dysplasia and Cervical Cancer in Rheumatoid Arthritis: A Cohort Study. *Arthritis Rheumatol.* 2016
732. Kim, S.K., et al. Prophylactic Central Neck Dissection Might Not Be Necessary in Papillary Thyroid Carcinoma: Analysis of 11,569 Cases from a Single Institution. *J Am Coll Surg.* 2016
733. Kirmani, B.H., et al. Long-Term Survival and Freedom From Reintervention After Off-Pump Coronary Artery Bypass Grafting: A Propensity-Matched Study. *Circulation.* 2016
734. Kishimoto, T., et al. Internet-Based Cognitive Behavioral Therapy for Social Anxiety with and without Guidance Compared to a Wait List in China: A Propensity Score Study. *Psychother Psychosom.* 2016
735. Kister, I., et al. Discontinuing disease-modifying therapy in MS after a prolonged relapse-free period: a propensity score-matched study. *J Neurol Neurosurg Psychiatry.* 2016
736. Kitamura, T., et al. Public-Access Defibrillation and Out-of-Hospital Cardiac Arrest in Japan. *N Engl J Med.* 2016
737. Koc, M., et al. Outcome of patients with right heart thrombi: the Right Heart Thrombi European Registry. *Eur Respir J.* 2016
738. Kohsaka, S., et al. Effects of Preoperative beta-Blocker Use on Clinical Outcomes after Coronary Artery Bypass Grafting: A Report from the Japanese Cardiovascular Surgery Database. *Anesthesiology.* 2016
739. Korhonen, P., et al. Pioglitazone use and risk of bladder cancer in patients with type 2 diabetes: retrospective cohort study using datasets from four European countries. *Bmj.* 2016
740. Krishna, S.G., et al. Effects of Bariatric Surgery on Outcomes of Patients With Acute Pancreatitis*. Clin Gastroenterol Hepatol.* 2016
741. Kronman, M.P., et al. Extended- Versus Narrower-Spectrum Antibiotics for Appendicitis Pediatrics 2016
742. Kubo, S., et al. Comparison of the efficacies of abatacept and tocilizumab in patients with rheumatoid arthritis by propensity score matching. *Ann Rheum Dis.* 2016
743. Kurlansky, P., et al. Coronary Artery Bypass Graft Versus Percutaneous Coronary Intervention: Meds Matter: Impact of Adherence to Medical Therapy on Comparative Outcomes*. Circulation.* 2016
744. Kyvernitakis, A., et al. Initial use of combination treatment does not impact survival of 106 patients with haematologic malignancies and mucormycosis: a propensity score analysis. *Clin Microbiol Infect*. 2016
745. Ladha, K.S., et al. Impact of Perioperative Epidural Placement on Postdischarge Opioid Use in Patients Undergoing Abdominal Surgery. *Anesthesiology.* 2016
746. Larsen, T.B., et al. Comparative effectiveness and safety of non-vitamin K antagonist oral anticoagulants and warfarin in patients with atrial fibrillation: propensity weighted nationwide cohort study. *Bmj.* 2016
747. Lasa, J.J., et al. Extracorporeal Cardiopulmonary Resuscitation (E-CPR) During Pediatric In-Hospital Cardiopulmonary Arrest Is Associated With Improved Survival to Discharge: A Report from the American Heart Association's Get With The Guidelines-Resuscitation (GWTG-R) Registry. *Circulation.* 2016
748. Lazarus, B., et al. Proton Pump Inhibitor Use and the Risk of Chronic Kidney Disease. *JAMA Intern Med.* 2016
749. Lee, J.H., et al. Comparative effectiveness of 3 bariatric surgery procedures: Roux-en-Y gastric bypass, laparoscopic adjustable gastric band, and sleeve gastrectomy. *Surg Obes Relat Dis.* 2016
750. Lee, S.S., et al. Long-term Quality of Life After Distal Subtotal and Total Gastrectomy: Symptom- and Behavior-oriented Consequences. *Ann Surg.* 2016
751. Leibowitz, M., et al. Association Between Achieved Low-Density Lipoprotein Levels and Major Adverse Cardiac Events in Patients With Stable Ischemic Heart Disease Taking Statin Treatment. *JAMA Intern Med.* 2016
752. Leta, T.H., et al. Outcomes of Unicompartmental Knee Arthroplasty After Aseptic Revision to Total Knee Arthroplasty: A Comparative Study of 768 TKAs and 578 UKAs Revised to TKAs from the Norwegian Arthroplasty Register (1994 to 2011). *J Bone Joint Surg Am*. 2016
753. Li, W., et al. Safety and Preliminary Efficacy of Early Tirofiban Treatment After Alteplase in Acute Ischemic Stroke Patients. *Stroke.* 2016
754. Lin, P.J., et al. Medicare Expenditures of Individuals with Alzheimer's Disease and Related Dementias or Mild Cognitive Impairment Before and After Diagnosis. *J Am Geriatr Soc.* 2016
755. Linden, S., et al. Risk of Suicidal Events With Atomoxetine Compared to Stimulant Treatment: A Cohort Study. *Pediatrics.* 2016
756. Lip, S., et al. Contrasting mortality risks among subgroups of treated hypertensive patients developing new-onset diabetes. *Eur Heart J.* 2016
757. Liu, P.H., et al. Surgical Resection Versus Radiofrequency Ablation for Single Hepatocellular Carcinoma </= 2 cm in a Propensity Score Model. *Ann Surg*. 2016
758. Liu, Y., et al. Frequent HIV Testing: Impact on HIV Risk Among Chinese Men Who Have Sex with Men. *J Acquir Immune Defic Syndr*. 2016
759. Lu, M., et al. Serum Biomarkers Indicate Long-term Reduction in Liver Fibrosis in Patients With Sustained Virological Response to Treatment for HCV Infection. *Clin Gastroenterol Hepatol.* 2016
760. Machuca, I., et al. Oral decontamination with aminoglycosides is associated with lower risk of mortality and infections in high-risk patients colonized with colistin-resistant, KPC-producing Klebsiella pneumoniae*. J Antimicrob Chemother*. 2016
761. MacIsaac, R.L., et al. Allopurinol and Cardiovascular Outcomes in Adults With Hypertension Hypertension 2016
762. Maeda, I., et al. Effect of continuous deep sedation on survival in patients with advanced cancer (J-Proval): a propensity score-weighted analysis of a prospective cohort study. *Lancet Oncol.* 2016
763. Mancia, G., et al. Cardiovascular outcomes at different on-treatment blood pressures in the hypertensive patients of the VALUE trial. *Eur Heart J*. 2016
764. Mannschreck, D., et al. Disparities in Surgical Care Among Women With Endometrial Cancer. *Obstet Gynecol.* 2016
765. Marcelli, D., et al. Dynamics of the erythropoiesis stimulating agent resistance index in incident hemodiafiltration and high-flux hemodialysis patients. *Kidney Int.* 2016
766. Marcus, J.L., et al. Use of Abacavir and Risk of Cardiovascular Disease Among HIV-Infected Individuals. *J Acquir Immune Defic Syndr*. 2016
767. Martin, A.S., et al. Trends in Severe Maternal Morbidity After Assisted Reproductive Technology in the United States, 2008-2012. *Obstet Gynecol.* 2016
768. Mazine, A., et al. Long-Term Outcomes of the Ross Procedure Versus Mechanical Aortic Valve Replacement: Propensity-Matched Cohort Study. *Circulation*. 2016
769. McDonald, J.S., et al. Is the Presence of a Solitary Kidney an Independent Risk Factor for Acute Kidney Injury after Contrast-enhanced CT? *Radiology.* 2016
770. Medbery, R.L., et al. Nodal Upstaging Is More Common with Thoracotomy than with VATS During Lobectomy for Early-Stage Lung Cancer: An Analysis from the National Cancer Data Base*. J Thorac Oncol.* 2016
771. Mellado, M., et al. Outcomes Associated With Inferior Vena Cava Filters Among Patients With Thromboembolic Recurrence During Anticoagulant Therapy. *JACC Cardiovasc Interv.* 2016
772. Mellinger, J.L., et al. Access to Subspecialty Care And Survival Among Patients With Liver Disease. *Am J Gastroenterol*. 2016
773. Michl, U., et al. Nerve-sparing Surgery Technique, Not the Preservation of the Neurovascular Bundles, Leads to Improved Long-term Continence Rates After Radical Prostatectomy. *Eur Urol.* 2016
774. Miller, S.C., et al. Palliative Care Consultations in Nursing Homes and Reductions in Acute Care Use and Potentially Burdensome End-of-Life Transitions. *J Am Geriatr Soc.* 2016
775. Minnerup, J., et al. Outcome After Thrombectomy and Intravenous Thrombolysis in Patients With Acute Ischemic Stroke: A Prospective Observational Study. *Stroke.* 2016
776. Mohanty, A., et al. Statins Are Associated With a Decreased Risk of Decompensation and Death in Veterans With Hepatitis C-Related Compensated Cirrhosis*. Gastroenterology.* 2016
777. Molnar, M.Z., et al. Survival of Elderly Adults Undergoing Incident Home Hemodialysis and Kidney Transplantation. *J Am Geriatr Soc.* 2016
778. Morgensztern, D., et al. Adjuvant Chemotherapy for Patients with T2N0M0 NSCLC. *J Thorac Oncol.* 2016
779. Moxon, C.A., et al. Safety of lumbar puncture in comatose children with clinical features of cerebral malaria. *Neurology.* 2016
780. Myslimi, F., et al. Orolingual Angioedema During or After Thrombolysis for Cerebral Ischemia. *Stroke.* 2016
781. Na, S.J., et al. Association Between Presence of a Cardiac Intensivist and Mortality in an Adult Cardiac Care Unit. *J Am Coll Cardiol.* 2016
782. Nagata, N., et al. Safety and Effectiveness of Early Colonoscopy in Management of Acute Lower Gastrointestinal Bleeding on the Basis of Propensity Score Matching Analysis. *Clin Gastroenterol Hepatol.* 2016
783. Nead, K.T., et al. Androgen Deprivation Therapy and Future Alzheimer's Disease Risk*. J Clin Oncol.* 2016
784. Nehra, D., et al. Acute Rehabilitation after Trauma: Does it Really Matter? *J Am Coll Surg.* 2016
785. Newman, T.B., et al. Retrospective Cohort Study of Phototherapy and Childhood Cancer in Northern California. *Pediatrics.* 2016
786. Noseworthy, P.A., et al. Direct Comparison of Dabigatran, Rivaroxaban, and Apixaban for Effectiveness and Safety in Nonvalvular Atrial Fibrillation. *Chest.* 2016
787. Nussbaum, D.P., et al. Preoperative or postoperative radiotherapy versus surgery alone for retroperitoneal sarcoma: a case-control, propensity score-matched analysis of a nationwide clinical oncology database*. Lancet Oncol*. 2016
788. Osthoff, M., et al. Low-Dose Acetylsalicylic Acid Treatment and Impact on Short-Term Mortality in Staphylococcus aureus Bloodstream Infection: A Propensity Score-Matched Cohort Study. *Crit Care Med.* 2016
789. Ou, S.M., et al. Long-Term Mortality and Major Adverse Cardiovascular Events in Sepsis Survivors. A Nationwide Population-based Study. *Am J Respir Crit Care Med*. 2016
790. Ouzounian, M., et al. Valve-Sparing Root Replacement Compared With Composite Valve Graft Procedures in Patients With Aortic Root Dilation*. J Am Coll Cardiol.* 2016
791. Pages, J., et al. Comparison of alcoholic chlorhexidine and povidone-iodine cutaneous antiseptics for the prevention of central venous catheter-related infection: a cohort and quasi-experimental multicenter study. *Intensive Care Med.* 2016
792. Palm, R., et al. Differences in Case Conferences in Dementia Specific vs Traditional Care Units in German Nursing Homes: Results from a Cross-Sectional Study. *J Am Med Dir Assoc.* 2016
793. Panczykowski, D., et al. Prophylactic Antiepileptics and Seizure Incidence Following Subarachnoid Hemorrhage: A Propensity Score-Matched Analysis. *Stroke.* 2016
794. Parikh, R.R., et al. Association of intensity-modulated radiation therapy on overall survival for patients with Hodgkin lymphoma. *Radiother Oncol.* 2016
795. Park, G.M., et al. Anatomic or Functional Evaluation as an Initial Test for Stable Coronary Artery Disease: A Propensity Score Analysis. *J Nucl Med.* 2016
796. Park, J.M., et al. Comparative Effectiveness of Prestroke Aspirin on Stroke Severity and Outcome. *Ann Neurol.* 2016
797. Patel, K.K., et al. Characteristics and Outcomes of Patients Presenting With Hypertensive Urgency in the Office Setting. *JAMA Intern Med.* 2016
798. Patel, S.D., et al. Comparison of angioplasty and bypass surgery for critical limb ischaemia in patients with infrapopliteal peripheral artery disease. *Br J Surg.* 2016
799. Perlas, A., et al. Anesthesia Technique and Mortality after Total Hip or Knee Arthroplasty: A Retrospective, Propensity Score-matched Cohort Study. *Anesthesiology.* 2016
800. Pilotto, A., et al. Warfarin Treatment and All-Cause Mortality in Community-Dwelling Older Adults with Atrial Fibrillation: A Retrospective Observational Study. *J Am Geriatr Soc.* 2016
801. Piscaglia, F., et al. Clinical patterns of hepatocellular carcinoma in nonalcoholic fatty liver disease: A multicenter prospective study. *Hepatology*. 2016
802. Porto, I., et al. Impact of Access Site on Bleeding and Ischemic Events in Patients With Non-ST-Segment Elevation Myocardial Infarction Treated With Prasugrel: The ACCOAST Access Substudy. *JACC Cardiovasc Interv.* 2016
803. Prescott, H.C., et al. Late mortality after sepsis: propensity matched cohort study. *Bmj.* 2016
804. Prochaska, J.J., et al. Likelihood of Unemployed Smokers vs Nonsmokers Attaining Reemployment in a One-Year Observational Study. *JAMA Intern Med*. 2016
805. Puckrein, G.A., et al. Impact of CMS Competitive Bidding Program on Medicare Beneficiary Safety and Access to Diabetes Testing Supplies: A Retrospective, Longitudinal Analysis. *Diabetes Care.* 2016
806. Puricel, S., et al. Bioresorbable Coronary Scaffold Thrombosis: Multicenter Comprehensive Analysis of Clinical Presentation, Mechanisms, and Predictors. *J Am Coll Cardiol.* 2016
807. Puymirat, E., et al. Correlates of pre-hospital morphine use in ST-elevation myocardial infarction patients and its association with in-hospital outcomes and long-term mortality: the FAST-MI (French Registry of Acute ST-elevation and non-ST-elevation Myocardial Infarction) programme. *Eur Heart J.* 2016
808. Puymirat, E., et al. beta blockers and mortality after myocardial infarction in patients without heart failure: multicentre prospective cohort study. *Bmj.* 2016
809. Pyo, J.H., et al. Long-Term Outcome of Endoscopic Resection vs. Surgery for Early Gastric Cancer: A Non-inferiority-Matched Cohort Study. *Am J Gastroenterol.* 2016
810. Qin, C., et al. Safety and Outcomes of Inpatient Compared with Outpatient Surgical Procedures for Ankle Fractures. *J Bone Joint Surg Am.* 2016
811. Ramos, R., et al. Statins for Prevention of Cardiovascular Events in a Low-Risk Population With Low Ankle Brachial Index. *J Am Coll Cardiol.* 2016
812. Ramsey, S.D., et al. Financial Insolvency as a Risk Factor for Early Mortality Among Patients With Cancer. *J Clin Oncol.* 2016
813. Renehan, A.G., et al. Watch-and-wait approach versus surgical resection after chemoradiotherapy for patients with rectal cancer (the OnCoRe project): a propensity-score matched cohort analysis. *Lancet Oncol.* 2016
814. Rivara, M.B., et al. Association of Vascular Access Type with Mortality, Hospitalization, and Transfer to In-Center Hemodialysis in Patients Undergoing Home Hemodialysis. *Clin J Am Soc Nephrol.* 2016
815. Rohatgi, N., et al. Surgical Comanagement by Hospitalists Improves Patient Outcomes: A Propensity Score Analysis. *Ann Surg.* 2016
816. Rosato, S., et al. Transcatheter Aortic Valve Implantation Compared With Surgical Aortic Valve Replacement in Low-Risk Patients. *Circ Cardiovasc Interv.* 2016
817. Rothschild, D.P., et al. Effect of Statin Therapy on Mortality in Older Adults Hospitalized with Coronary Artery Disease: A Propensity-Adjusted Analysis. *J Am Geriatr Soc.* 2016
818. Rusthoven, C.G., et al. Improved Survival With Prostate Radiation in Addition to Androgen Deprivation Therapy for Men With Newly Diagnosed Metastatic Prostate Cancer*. J Clin Oncol.* 2016
819. Sagara, Y., et al. Patient Prognostic Score and Associations With Survival Improvement Offered by Radiotherapy After Breast-Conserving Surgery for Ductal Carcinoma In Situ: A Population-Based Longitudinal Cohort Study. *J Clin Oncol.* 2016
820. Salado-Rasmussen, K., et al. Serological Response to Treatment of Syphilis with Doxycycline Compared with Penicillin in HIV-infected Individuals. *Acta Derm Venereol*. 2016
821. Sasabuchi, Y., et al. Risks and Benefits of Stress Ulcer Prophylaxis for Patients With Severe Sepsis. *Crit Care Med.* 2016
822. Sato, Y., et al. Tooth Loss and Decline in Functional Capacity: A Prospective Cohort Study from the Japan Gerontological Evaluation Study*. J Am Geriatr Soc.* 2016
823. Scarborough, J.E., et al. Nonoperative Management Is as Effective as Immediate Splenectomy for Adult Patients with High-Grade Blunt Splenic Injury. *J Am Coll Surg*. 2016
824. Scheitz, J.F., et al. Statins and risk of poststroke hemorrhagic complications. *Neurology.* 2016
825. Scherrer, J.F., et al. Prescription Opioid Duration, Dose, and Increased Risk of Depression in 3 Large Patient Populations. *Ann Fam Med*. 2016
826. Schneeweiss, S., et al. Short-term risk of liver and renal injury in hospitalized patients using micafungin: a multicentre cohort study. *J Antimicrob Chemother.* 2016
827. Schoenfeld, S.R., et al. Statin use and mortality in rheumatoid arthritis: a general population-based cohort study. *Ann Rheum Dis.* 2016
828. See, R.B., et al. Extended Motor Evoked Potentials Monitoring Helps Prevent Delayed Paraplegia After Aortic Surgery. *Ann Neurol.* 2016
829. Seisen, T., et al. Efficacy of High-Intensity Local Treatment for Metastatic Urothelial Carcinoma of the Bladder: A Propensity Score-Weighted Analysis From the National Cancer Data Base. *J Clin Oncol.* 2016
830. Sepriano, A., et al. Effect of Comedication With Conventional Synthetic Disease-Modifying Antirheumatic Drugs on Retention of Tumor Necrosis Factor Inhibitors in Patients With Spondyloarthritis: A Prospective Cohort Study. *Arthritis Rheumatol.* 2016
831. Shah, N., et al. Therapeutic Hypothermia and Stent Thrombosis: A Nationwide Analysis. *JACC Cardiovasc Interv.* 2016
832. Shander, A., et al. Outcomes of Protocol-Driven Care of Critically Ill Severely Anemic Patients for Whom Blood Transfusion Is Not an Option. *Crit Care Med.* 2016
833. Sheth, T.N., et al. Optical Coherence Tomography-Guided Percutaneous Coronary Intervention in ST-Segment-Elevation Myocardial Infarction: A Prospective Propensity-Matched Cohort of the Thrombectomy Versus Percutaneous Coronary Intervention Alone Trial. *Circ Cardiovasc Interv.* 2016
834. Shetty, V., et al. Methamphetamine Users Have Increased Dental Disease: A Propensity Score Analysis. *J Dent Res.* 2016
835. Shih, C.J., et al. Cardiovascular Outcomes of Dipeptidyl Peptidase-4 Inhibitors in Elderly Patients With Type 2 Diabetes: A Nationwide Study. *J Am Med Dir Assoc*. 2016
836. Shih, C.J., et al. Risks of Death and Stroke in Patients Undergoing Hemodialysis With New-Onset Atrial Fibrillation: A Competing-Risk Analysis of a Nationwide Cohort. *Circulation*. 2016
837. Shindoh, J., et al. Complete removal of the tumor-bearing portal territory decreases local tumor recurrence and improves disease-specific survival of patients with hepatocellular carcinoma. *J Hepatol.* 2016
838. Shofty, B., et al. Intrathecal or intraventricular therapy for post-neurosurgical Gram-negative meningitis: matched cohort study*. Clin Microbiol Infect*. 2016
839. Simonetti, A.F., et al. Declining mortality among hospitalized patients with community-acquired pneumonia. *Clin Microbiol Infect.* 2016
840. Singh, S., et al. Comparative Effectiveness and Safety of Anti-Tumor Necrosis Factor Agents in Biologic-Naive Patients With Crohn's Disease. *Clin Gastroenterol Hepatol.* 2016
841. Singh, S., et al. Comparative effectiveness and safety of infliximab and adalimumab in patients with ulcerative colitis. *Aliment Pharmacol Ther.* 2016
842. Sirker, A., et al. Outcomes From Selective Use of Thrombectomy in Patients Undergoing Primary Percutaneous Coronary Intervention for ST-Segment Elevation Myocardial Infarction: An Analysis of the British Cardiovascular Intervention Society/National Institute for Cardiovascular Outcomes Research (BCIS-NICOR) Registry, 2006-2013. *JACC Cardiovasc Interv*. 2016
843. Sorelius, K., et al. Nationwide Study of the Treatment of Mycotic Abdominal Aortic Aneurysms Comparing Open and Endovascular Repair. *Circulation.* 2016
844. Sperry, B.W., et al. Efficacy of Chemotherapy for Light-Chain Amyloidosis in Patients Presenting With Symptomatic Heart Failure. *J Am Coll Cardiol.* 2016
845. Sposito, C., et al. Propensity score analysis of outcomes following laparoscopic or open liver resection for hepatocellular carcinoma. *Br J Surg.* 2016
846. Stroup, T.S., et al. Comparative Effectiveness of Clozapine and Standard Antipsychotic Treatment in Adults With Schizophrenia. *Am J Psychiatry.* 2016
847. Sutton, N.R., et al. Outcomes of Patients With Atrial Fibrillation Undergoing Percutaneous Coronary Intervention. *J Am Coll Cardiol*. 2016
848. Suzuki, S., et al. The Efficacy and Tolerability of a Triple Therapy Containing a Potassium-Competitive Acid Blocker Compared With a 7-Day PPI-Based Low-Dose Clarithromycin Triple Therapy. *Am J Gastroenterol.* 2016
849. Tagami, T., et al. Changes in Therapeutic Hypothermia and Coronary Intervention Provision and In-Hospital Mortality of Patients With Out-of-Hospital Cardiac Arrest: A Nationwide Database Study. *Crit Care Med.* 2016
850. Tagami, T., et al. Prophylactic Antibiotics May Improve Outcome in Patients With Severe Burns Requiring Mechanical Ventilation: Propensity Score Analysis of a Japanese Nationwide Database. *Clin Infect Dis*. 2016
851. Takagi, K., et al. Comparison Between 1- and 2-Stent Strategies in Unprotected Distal Left Main Disease: The Milan and New-Tokyo Registry. *Circ Cardiovasc Interv.* 2016
852. Tamburino, C., et al. 1-Year Outcomes of Everolimus-Eluting Bioresorbable Scaffolds Versus Everolimus-Eluting Stents: A Propensity-Matched Comparison of the GHOST-EU and XIENCE V USA Registries. *JACC Cardiovasc Interv.* 2016
853. Tan, D.S., et al. Comparative Efficacy of Ceritinib and Crizotinib as Initial ALK-Targeted Therapies in Previously Treated Advanced NSCLC: An Adjusted Comparison with External Controls*. J Thorac Oncol.* 2016
854. Tan, T.L., et al. Polymicrobial Periprosthetic Joint Infections: Outcome of Treatment and Identification of Risk Factors. *J Bone Joint Surg Am.* 2016
855. Tapper, E.B., et al. Evaluation of proton pump inhibitor use on treatment outcomes with ledipasvir and sofosbuvir in a real-world cohort study. *Hepatology.* 2016
856. Tashiro, J., et al. Reduced Hospital Mortality With Surgical Ligation of Patent Ductus Arteriosus in Premature, Extremely Low Birth Weight Infants: A Propensity Score-matched Outcome Study*. Ann Surg.* 2016
857. Thongprayoon, C., et al. AKI after Transcatheter or Surgical Aortic Valve Replacement. *J Am Soc Nephrol.* 2016
858. Thourani, V.H., et al. Transcatheter aortic valve replacement versus surgical valve replacement in intermediate-risk patients: a propensity score analysis. *Lancet.* 2016
859. Tian, F., et al. Propensity score-matched analysis of robotic versus open surgical enucleation for small pancreatic neuroendocrine tumours. *Br J Surg.* 2016
860. Toh, S., et al. Risk for Hospitalized Heart Failure Among New Users of Saxagliptin, Sitagliptin, and Other Antihyperglycemic Drugs: A Retrospective Cohort Study. *Ann Intern Med.* 2016
861. Tolppanen, A.M., et al. Antipsychotic Use and Risk of Hospitalization or Death Due to Pneumonia in Persons With and Those Without Alzheimer Disease. *Chest*. 2016
862. Trabuco, E.C., et al. Association of Ovary-Sparing Hysterectomy With Ovarian Reserve. *Obstet Gynecol.* 2016
863. Treat, E., et al. Use of Polyethylene Glycol Electrolyte Solution Expedites Return of Bowel Function and Facilitates Early Discharge after Kidney Transplantation. *J Am Coll Surg.* 2016
864. Tseng, V.L., et al. Cataract Surgery and Mortality in the United States Medicare Population. *Ophthalmology.* 2016
865. Tsivgoulis, G., et al. Statin pretreatment is associated with better outcomes in large artery atherosclerotic stroke. *Neurology.* 2016
866. Unroe, K.T., et al. Effect of Hospice Use on Costs of Care for Long-Stay Nursing Home Decedents*. J Am Geriatr Soc*. 2016
867. Uppal, S., et al. Prophylactic Antibiotic Choice and Risk of Surgical Site Infection After Hysterectomy. *Obstet Gynecol*. 2016
868. Vallet, H., et al. Infliximab Versus Adalimumab in the Treatment of Refractory Inflammatory Uveitis: A Multicenter Study From the French Uveitis Network. *Arthritis Rheumatol*. 2016
869. Valuck, R.J., et al. Comparison of antidepressant classes and the risk and time course of suicide attempts in adults: propensity matched, retrospective cohort study*. Br J Psychiatry.* 2016
870. Wada, T., et al. Outcomes of Argatroban Treatment in Patients With Atherothrombotic Stroke: Observational Nationwide Study in Japan. *Stroke.* 2016
871. Wafaisade, A., et al. Prehospital administration of tranexamic acid in trauma patients. *Crit Care.* 2016
872. Walkey, A.J., et al. Practice Patterns and Outcomes of Treatments for Atrial Fibrillation During Sepsis: A Propensity-Matched Cohort Study. *Chest.* 2016
873. Wan, X., et al. Ulinastatin administration is associated with a lower incidence of acute kidney injury after cardiac surgery: a propensity score matched study. *Crit Care.* 2016
874. Wang, B.Y., et al. Thoracoscopic Lobectomy Produces Long-Term Survival Similar to That with Open Lobectomy in Cases of Non-Small Cell Lung Carcinoma: A Propensity-Matched Analysis Using a Population-Based Cancer Registry*. J Thorac Oncol.* 2016
875. Wang, S.Y., et al. Preoperative Breast Magnetic Resonance Imaging and Contralateral Breast Cancer Occurrence Among Older Women With Breast Cancer. *J Clin Oncol.* 2016
876. Warschkow, R., et al. Improved Survival After Primary Tumor Surgery in Metastatic Breast Cancer: A Propensity-adjusted, Population-based SEER Trend Analysis. *Ann Surg*. 2016
877. Weimer, J.M., et al. Withdrawal of Life-Sustaining Therapy in Patients With Intracranial Hemorrhage: Self-Fulfilling Prophecy or Accurate Prediction of Outcome? *Crit Care Med.* 2016
878. Welsh, F.K., et al. Propensity score-matched outcomes analysis of the liver-first approach for synchronous colorectal liver metastases. *Br J Surg.* 2016
879. White, R.H., et al. Outcomes After Vena Cava Filter Use in Noncancer Patients With Acute Venous Thromboembolism: A Population-Based Study. *Circulation.* 2016
880. Whittaker, W., et al. Associations between Extending Access to Primary Care and Emergency Department Visits: A Difference-In-Differences Analysis. *PLoS Med*. 2016
881. Wickremasinghe, A.C., et al. Neonatal Phototherapy and Infantile Cancer*. Pediatrics.* 2016
882. Wiewel, M.A., et al. Chronic antiplatelet therapy is not associated with alterations in the presentation, outcome, or host response biomarkers during sepsis: a propensity-matched analysis. *Intensive Care Med*. 2016
883. Wong, A.Y., et al. Cardiovascular outcomes associated with use of clarithromycin: population based study. *Bmj.* 2016
884. Wood, A.D., et al. Rheumatic Mitral Valve Disease Is Associated With Worse Outcomes in Stroke: A Thailand National Database Study. *Stroke.* 2016
885. Wright, J.D., et al. Comparative Effectiveness of Minimally Invasive Hysterectomy for Endometrial Cancer*. J Clin Oncol.* 2016
886. Wright, J.D., et al. Influence of Lymphadenectomy on Survival for Early-Stage Endometrial Cancer. *Obstet Gynecol.* 2016
887. Xie, Y., et al. Proton Pump Inhibitors and Risk of Incident CKD and Progression to ESRD. *J Am Soc Nephrol.* 2016
888. Yang, C.P., et al. Migraine and Risk of Ocular Motor Cranial Nerve Palsies: A Nationwide Cohort Study. *Ophthalmology*. 2016
889. Yang, T., et al. Perioperative blood transfusion does not influence recurrence-free and overall survivals after curative resection for hepatocellular carcinoma: A Propensity Score Matching Analysis. *J Hepatol.* 2016
890. Yu, T.M., et al. Risk of cancer in patients with polycystic kidney disease: a propensity-score matched analysis of a nationwide, population-based cohort study. *Lancet Oncol.* 2016
891. Yun, J.A., et al. Oncologic Outcomes of Single-incision Laparoscopic Surgery Compared With Conventional Laparoscopy for Colon Cancer. *Ann Surg.* 2016
892. Zavada, J., et al. A tailored approach to reduce dose of anti-TNF drugs may be equally effective, but substantially less costly than standard dosing in patients with ankylosing spondylitis over 1 year: a propensity score-matched cohort study. *Ann Rheum Dis*. 2016
893. Zaydfudim, V.M., et al. Liver Resection and Transplantation for Patients With Hepatocellular Carcinoma Beyond Milan Criteria. *Ann Surg.* 2016
894. Zheng, Z., et al. Coronary Artery Bypass Graft Surgery and Percutaneous Coronary Interventions in Patients With Unprotected Left Main Coronary Artery Disease. *JACC Cardiovasc Interv*. 2016
