# Supplementary material for: A review of the use of propensity score diagnostics in papers published in high-ranking medical journals
Source: BMC Med Res Methodol. 2020 May 27;20:132. doi: 10.1186/s12874-020-00994-0 (PMC7251670; doi:10.1186/s12874-020-00994-0)
Supplement: Supplementary file 3 — Additional file 3. Further details on Research Areas [file 12874_2020_994_MOESM3_ESM.docx]

Additional file 3: Further details on Research Areas

| **Research area** | **Number of articles (%)** |
| --- | --- |
| Anaesthesiology | 21 (2.35%) |
| Cardiac and Cardiovascular Systems | 115 (12.86%) |
| Clinical Neurology | 17 (1.90%) |
| Critical Care Medicine | 66 (7.38%) |
| Dentistry, Oral Surgery & Medicine | 2 (0.22%) |
| Dermatology | 5 (0.56%) |
| Emergency Medicine | 4 (0.45%) |
| Endocrinology & Metabolism | 6 (0.67%) |
| Gastroenterology & Hepatology | 51 (5.70%) |
| Geriatrics and Gerontology | 29 (3.24%) |
| Health Care Sciences & Services | 4 (0.45%) |
| Haematology | 6 (0.67%) |
| Immunology | 1 (0.11%) |
| Infectious Diseases | 46 (5.15%) |
| Medicine, General & Internal | 81 (9.06%) |
| Medicine, Research & Experimental | 1 (0.11%) |
| Nutrition and Dietetics | 4 (0.45%) |
| Obstetrics & Gynaecology | 12 (1.34%) |
| Oncology | 36 (4.03%) |
| Paediatrics | 17 (1.90%) |
| Peripheral Vascular Disease | 17 (1.90%) |
| Pharmacology & Pharmacy | 3 (0.34%) |
| Primary Health Care | 3 (0.34%) |
| Psychiatry | 12 (1.34%) |
| Public, Environmental and Occupational | 6 (0.67%) |
| Radiology, Nuclear Medicine and Medical | 21 (2.35%) |
| Rehabilitation | 1 (0.11%) |
| Rheumatology | 32 (3.58%) |
| Sports Sciences | 1 (0.11%) |
| Substance Abuse | 2 (0.22%) |
| Surgery | 110 (12.30%) |
| Urology and Nephrology | 46 (5.15%) |
| Cardiac and Cardiovascular Systems AND Peripheral Vascular Disease | 20 (2.24%) |
| Cardiac and Cardiovascular Systems AND Radiology, Nuclear Medicine and Medical Imaging | 8 (0.89%) |
| Critical Care Medicine AND Respiratory System | 19 (2.13%) |
| Peripheral Vascular Disease AND Haematology | 19 (2.13%) |
| Respiratory System AND Surgery | 9 (1.01%) |
